# Physical activity and functional limitations in older adults: a systematic review related to Canada's Physical Activity Guidelines

**DOI:** 10.1186/1479-5868-7-38

**Published:** 2010-05-11

**Authors:** Donald H Paterson, Darren ER Warburton

**Affiliations:** 1 School of Kinesiology, University of Western Ontario, London, Ontario, Canada; 2 Canadian Centre for Activity and Aging, University of Western Ontario, London, Ontario, Canada; 3 Cardiovascular Physiology Rehabilitation Laboratory, University of British Columbia, Vancouver, British Columbia, Canada; 4 Experimental Medicine Programme, University of British Columbia, Vancouver, British Columbia, Canada

## Abstract

**Background:**

The purpose was to conduct systematic reviews of the relationship between physical activity of healthy community-dwelling older (>65 years) adults and outcomes of functional limitations, disability, or loss of independence.

**Methods:**

Prospective cohort studies with an outcome related to functional independence or to cognitive function were searched, as well as exercise training interventions that reported a functional outcome. Electronic database search strategies were used to identify citations which were screened (title and abstract) for inclusion. Included articles were reviewed to complete standardized data extraction tables, and assess study quality. An established system of assessing the level and grade of evidence for recommendations was employed.

**Results:**

Sixty-six studies met inclusion criteria for the relationship between physical activity and functional independence, and 34 were included with a cognitive function outcome. Greater physical activity of an aerobic nature (categorized by a variety of methods) was associated with higher functional status (expressed by a host of outcome measures) in older age. For functional independence, moderate (and high) levels of physical activity appeared effective in conferring a reduced risk (odds ratio ~0.5) of functional limitations or disability. Limitation in higher level performance outcomes was reduced (odds ratio ~0.5) with vigorous (or high) activity with an apparent dose-response of moderate through to high activity. Exercise training interventions (including aerobic and resistance) of older adults showed improvement in physiological and functional measures, and suggestion of longer-term reduction in incidence of mobility disability. A relatively high level of physical activity was related to better cognitive function and reduced risk of developing dementia; however, there were mixed results of the effects of exercise interventions on cognitive function indices.

**Conclusions:**

There is a consistency of findings across studies and a range of outcome measures related to functional independence; regular aerobic activity and short-term exercise programmes confer a reduced risk of functional limitations and disability in older age. Although a precise characterization of a minimal or effective physical activity dose to maintain functional independence is difficult, it appears moderate to higher levels of activity are effective and there may be a threshold of at least moderate activity for significant outcomes.

## Introduction

This review is focused on the relationship between physical activity and functional outcomes for older adults (>65 years of age, but <85 years of age) in the general "community-dwelling" population. A significant body of research has also examined the relationship between physical activity and outcomes of premature all-cause mortality and morbidity (of various chronic diseases). This evidence has been detailed in the companion paper reviewing physical activity for "adults" [1] and has been reviewed previously (see [2,3]). This research has demonstrated compelling support for the health benefits of physical activity across the adult lifespan.

Previous studies of morbidity and mortality in adults have provided important information for "older" individuals. In fact, studies with a group mean age of 45-50 years often included participants who were at least 65 years of age, and a follow-up period of 5 years or more (see [2]). The findings of these studies are weighted strongly by the outcome incidence or prevalence being much greater in the older groups, and thus the information is very applicable to the older population. Indeed some studies have focused on older adults; and some have sub-divided their population by age facilitating interpretations for different age groups. In gestalt overview these latter groups of studies suggest a somewhat lower requisite "intensity" of exercise (even in relative terms) to delay mortality or reduce the incidence of disease for older adults compared to middle-aged groups [2]. A number of these studies have specifically examined walking and whether the "amount" of walking relates to outcomes. Indeed these data show that walking is a physical activity that leads to decreases in all-cause mortality and in morbidity. The amount of walking is often equivalent to approximately 1000 to 1500 kcal/week (4200 to 6300 kJ/week), but sometimes less (minimally 500 kcal/week; 2100 kJ/week); and the intensity within the walking domain may be important for health benefits (see [4]), but again, minimally, even normal walking speeds have been related to better outcomes. Further research is clearly warranted to evaluate the minimal requisite intensity of physical activity or exercise for (morbidity and mortality) health benefits in the older adults.

It is essential to highlight that many older individuals consider the capacity to carry out activities of daily living (i.e., functional independence) to be of greater concern than prevention of disease [5-7]. Moreover, the health-related quality of life and life expectancy of individuals who live in a dependent state is greatly reduced. The associated nursing home or long-term care health-care costs of are high. Research (as reviewed in this paper) has increasingly examined the role that habitual physical activity plays in the maintenance of functional independence. However, questions remain regarding the minimal, and dose-response characteristics of the intensity and amount of physical activity or exercise required for the maintenance of functional independence in older adults.

Accordingly, the present paper analyses the relationship between physical activity and outcomes related to functional limitations, disability and loss of independence in older adults. The primary purpose of this systematic review is to examine the role of physical activity in the maintenance of functional independence in the elderly. Furthermore, it is our intention to focus on the functional (physical) and cognitive determinants of independence in healthy (asymptomatic) individuals and derive a recommended types of physical activity and volume and intensity dose-response relationships required to achieve these health benefits.

The present "aging population" will result in substantial increase in the numbers and proportion of older adults. Aging is characterized by loss of function and prevalence of chronic diseases and older adults are among the most sedentary (physically inactive) segment of society. In many respects the increased life expectancy now appears to be exceeding our ability to maintain function and functional independence. A large proportion of older adults may live perilously close to important thresholds of physically ability that may render them dependent. The reduced quality of life and the social and economic (health-care) consequences are staggering. In terms of public health the benefits that may be derived with a more physically active older population may be essential in the maintenance of our health-care system.

## Methods

In analysis of physical activity, specifically for older adults, it was decided that an important aspect was maintenance of functional abilities and functional independence (i.e., "performance-related fitness") with functional outcomes to supplement the information on "health-related fitness" of all-cause mortality and morbidity outcomes, reviewed in the adult paper by Warburton et al. [1] and by Paterson et al. [2]. Functional outcomes included assessments of functional status decline, impairment or functional limitations, or disability, including self-report questionnaire assessments or measured physical performance tests. Thus prospective cohort studies of the relationship between physical activity and functional outcomes were reviewed. Determining the nature of the physical activity that was related to outcome measures required close inspection of the criteria set out in each study, as there was no consistent categorization of physical activity groupings or of the components of physical activity that might relate to the dose-response. In some studies the physical activity was quantified by volume (as a total energy expenditure, or as a frequency and duration of activities) and other studies also attempted to account for the relative intensity of the activities (light, moderate, vigorous) and types of activity (walking, exercising, sports play, recreation, household chores). Thus, the level of physical activity was determined from analysis of types of activities that were reported for each activity level in each study; for example, in a number of studies there were only two activity groups but to be in the higher group there had to be report of vigorous activities (sports) or walking of about 1 hour per day. This analysis was used to categorize the physical activities or physical activity groups that were related to the outcome as those of: vigorous activities and/or high volume of systematic activity (walking for exercise); moderately-vigorous groups as the activities included vigorous activity (not included in moderate group) or walking for exercise, but at a lower volume than for the vigorous high-volume group; moderate levels of activity from participation in normal walking or gardening with a volume of 3-5 days/week and 30 min per day; participation mainly in light activities of daily living with only occasional walking or gardening.

Additionally it was recognized that as well as reviewing studies of the relationship of physical activity with outcomes in the long-term, or the effects of life-long physical activity (as characterized in prospective cohort studies with a relatively long-term follow-up), it was also essential to analyse the short-term outcomes of the more immediate effects consequent to physical activity interventions over a few weeks to months. Thus, the more immediate effects of exercise training programmes on physiological outcomes related to increased cardiorespiratory fitness or strength aspects which were reviewed in Paterson et al. [2] were supplemented in this review with results of short-term exercise interventions that reported functional outcomes. Thus, aerobic and strength exercise training programmes with functional outcomes were reviewed.

### Criteria for considering studies for this review

The review was restricted to published, original, scientific journal manuscripts written in English. Studies evaluating the relationship between the "intervention" of any physical activity (or cardiorespiratory or strength assessment) and outcomes of variables related to functional independence and of cognitive function were included. Population samples included asymptomatic "community-dwelling" older adults between 65 and 85 years of age.

The review was restricted to participants with "minimal" initial impairment or functional inability; thus, studies of rehabilitation, subject groups initially very old (e.g., > 85 years of age), those considered to be "frail", individuals in nursing home environments, and those in long-term care were excluded. Studies of samples with specific disease or conditions (e.g., diabetes, heart disease, prior stroke) were also excluded, with the exception of studies including participants with arthritis as this condition affects a large portion of older adults.

As a functional outcome, the extensive literature regarding "falls" as a major outcome was not included, since a falls outcome differs from an outcome of disability or functional limitation, and studies have suggested that falls can be prevented through specific modification to the physical activity prescription (i.e., by including balance activities). Additionally, outcomes of anxiety and depression are not reviewed as these are specific clinical (or sub-clinical) conditions and their treatment may allow modification of the "general" physical activity "prescription" to achieve an outcome related to the specific clinical condition. Similarly, studies examining persons with pre-existing dementia and/or Alzheimer's disease were excluded from the analyses.

### Search strategy

Literature searches were conducted in the following electronic bibliographical databases:

• MEDLINE (1966-March 2008, OVID Interface);

• EMBASE (1980-March 2008, OVID Interface),

• CINAHL (1982-March 2008, OVID Interface);

• PsycINFO (1840-March 2008, Scholars Portal Interface);

• Cochrane Library (-March 2008),

• SPORTDiscus (-March 2008).

The Medical Subject Headings (MeSH) were kept broad. See Tables s1 and s2 (see additional file 1) for examples of the complete MEDLINE search strategy and keywords used. The electronic search strategies were created and carried out by researchers experienced with systematic reviews of the literature. Searches were limited to the English language, human subjects, and participants over age 65 years. The citations and applicable electronic versions of the article (where available) were downloaded to an online research management system (RefWorks, Bethesda, Maryland, USA). Duplicate citations were removed.

### Screening

Two reviewers (research staff) independently screened the title and abstract of the citations to identify potential articles for inclusion. The reviewers were not blinded to the authors or journals. For those articles that appeared relevant, the full text study report was obtained and data was extracted using a common template. Selected articles were retrieved electronically or manually via the Canadian interlibrary system. Disagreements regarding inclusion were resolved through discussion with a third reviewer. All studies that were excluded during the citation and full-article screening processes were recorded (this list of excluded studies is available upon request). Reference lists of key studies and reviews in the field were also cross-referenced in order to identify further literature and references from personal files were added. (It is noted that this cross-referencing yielded many studies not found in the electronic search, particularly for prospective cohort studies of the relationship between physical activity and functional outcomes. The search strategy problems appear to relate mainly to two factors: the study sample ages were often given for the study baseline (as < 65 years) but with the follow-up time the data actually met the inclusion age (>65 years) (and these references became included from the cross-reference or personal files search); the terms related to functional outcomes are not standardized and often the functional outcomes were not in the title or abstract.)

### Data Extraction

Two reviewers (research staff) completed standardized data extraction forms. One person performed the data extraction for each paper assigned to them and the extraction was verified by another reviewer. Information was extracted regarding the study design, the country where the study was conducted, the participant characteristics, the sample size, the objectives of the study, the methodologies employed, the major outcomes of functional decline or limitation, disability or dependence, and the results and conclusions of the studies. The reviewers were not blinded to the journal or the author names when extracting information from the articles. Subsequently, one author (and one research staff) extracted further detail or clarifications as needed in assembling the Tables, and completed the tabular data for references obtained from cross-referencing subsequent to the electronic search.

### Level of Evidence

The approach used to establish the level and grade of evidence was consistent with that used during creation of the "Canadian clinical practice guidelines on the management and prevention of obesity in adults and children" [8]. The level of evidence provides information regarding the strength of the evidence in favour of physical activity/exercise in the primary prevention of functional limitations, disability or dependence. This evaluation process is based on a pre-defined and objective criteria (see Table s3 in additional file 1). Thus, grade and level of evidence were assessed for both general recommendations regarding physical activity for older adults, and also for more specific guidelines regarding the appropriate dose of physical activity and the strength of the data supporting the recommendation. A physical activity guideline that receives the highest grading would indicate that the benefits clearly outweigh the risk and receive a strong support. However in the present review "risks" of physical activity were not assessed. Studies reviewed in this paper did not report or assess risks such as acute cardiovascular events associated with increased physical activity (see American Heart Association Scientific Statement by Thompson et al. [9]). The studies reviewed also did not report on the incidence of injury in more physically active individuals, or with increases in physical activity.

### Quality Assessment

The Downs and Black [10] scale was selected to assess the quality of each study as it is appropriate to evaluate non-randomised investigations, and it contained the highest number of relevant items for the needs of this review. However, as not all items were relevant to the various study types included in this review, a modified version of the checklist was employed for each of prospective, RCT (randomised control trials), and non-RCT study types. Thus, the quality of each study was also established similar to the method of Gorber et al. [11] to include the most relevant components of the scoring tool. Therefore, a modified version of the Downs and Black checklist was used with the final checklist consisting of 12 items with a maximum score of 12 points for the studies of a prospective cohort design (with functional outcomes or cognitive outcomes). For studies of exercise training interventions the scale was modified to 22 items or an additional one item for the RCT exercise training interventions and total score of 23 or 24, respectively. Higher scores reflected a superior quality of investigation.

### Integration of Findings

Due to the heterogeneity across study populations, methods used, and outcomes assessed in drawing conclusions and recommendations from the review we conducted a narrative synthesis of the results.

## Results

### Functional Independence

#### Search Results: Physical Activity and Functional Independence

**Figure 1:**
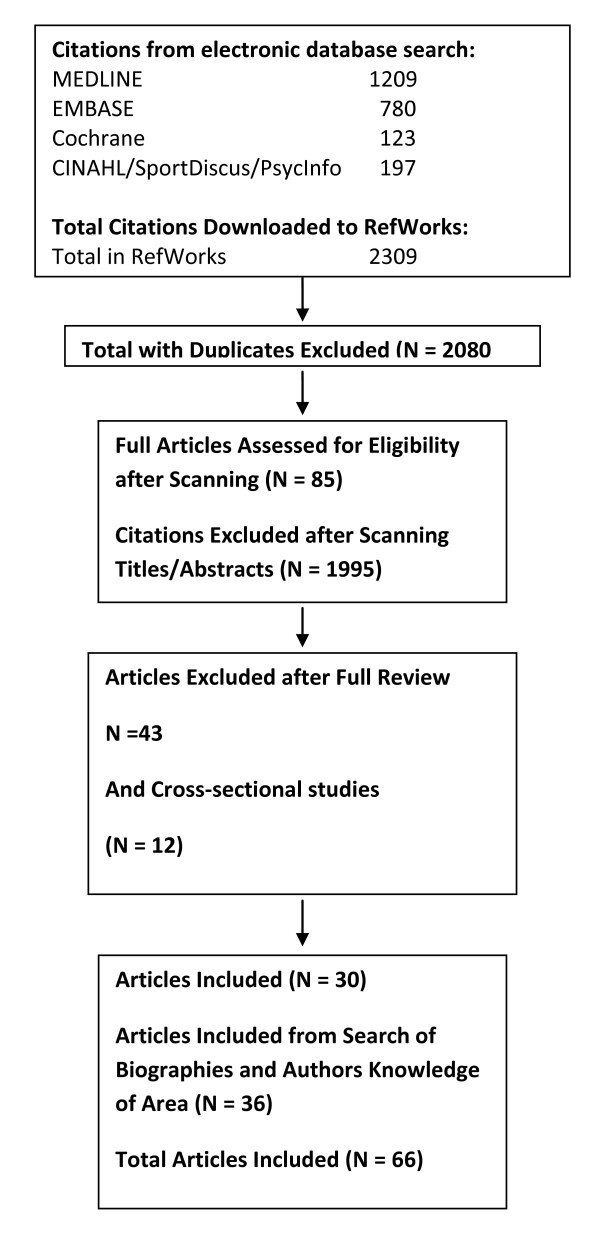
**Results of the literature search for functional limitations**.

A total of 2,309 citations were identified during the electronic database search (Figure 1). Of these citations, 1,209 were identified in MEDLINE, 780 in EMBASE, 123 in Cochrane, and 197 in the CINAHL/SportDiscus/PsychInfo search. A total of 229 duplicates were found, leaving a total of 2,080 unique citations. A total of 1,735 articles were excluded after scanning, leaving a total of 345 articles. From these articles 260 were excluded after further (abstract) review leaving 85 articles. The reasons for exclusion included: participant group did not meet the inclusion criteria for age, absence of disease (i.e., study was of a clinical population) or for life-style (were nursing home or long-term care residents or of a "frail" sample or >85 years of age versus community-dwelling older adults); functional measure not reported; physical activity level or exercise capacity not reported; or the citation was a review, dissertation, thesis, or abstract. With full review 24 further papers did not meet inclusion criteria, leaving 61 articles. A further 19 (now n = 42) were omitted as the subject sample was actually a frail, not community-living group (n = 15), with others omitted when detailed reading showed no acceptable functional outcome measure (these had included falls or anxiety and depression outcomes). Lastly (as explained later) it was decided not to include studies of cross-sectional design regarding the relationships between physical activity and functional outcome, and this eliminated a further 12 studies. Thus the search revealed 30 studies fully reviewed for inclusion in the systematic review and summarized on the extraction Tables. Additional literature was tracked from reference citations and author files. These searches provided an additional 36 studies (and 11 supplementary reports). Therefore, a total of 66 unique studies were included in the systematic review of the literature regarding the relationship between physical activity and functional independence.

Data from longitudinal or prospective studies with an outcome of functional limitation were considered most influential in our analyses. Cross-sectional or retrospective studies were considered; however, these studies were often limited as they did not adequately discriminate whether the factors associated with functional limitations antedated the functional decline or were a consequence of the functional decline. Thus, for cross-sectional or retrospective analyses of the relationships between physical activity and functional outcomes we have not performed an exhaustive systematic review but have cited some studies that were influential in the field. Exercise training studies that included an outcome of function were also examined. These included exercise training interventions of either aerobic or aerobic supplemented with resistance training, or programmes of resistance training alone. These studies allow for the examination of the more immediate effects of increased physical activity, but usually only assessed proxy measures of function that might be related to subsequent limitations or disability, whereas, the longitudinal studies generally indicated the relationships of physical activity status with function over a more prolonged period of time.

#### Prospective cohort studies of physical activity and functional limitations in older adults

##### Description of Studies (population, intervention, outcome)

The present review captured articles analysing associations between physical activity or cardiorespiratory fitness or muscle (strength) fitness with outcomes of impairment or functional limitation (or functional status decline) or disability in older adults using a longitudinal, prospective cohort design. Table s4 (see additional file 2) provides summary details of 35 prospective cohort studies (plus 7 "supplemental" reports from the same data base with different outcome measures or with a longer follow-up). Table s4 (see additional file 2) captures studies from 1995 to 2008; earlier studies have been frequently reviewed (see [12,13]). The 35 studies included 83,740 participants with study sizes ranging from 141 to 10,209 participants. About half of the studies (n = 19) were of larger sample sizes with subject numbers from ~1000 to 10,000 (median 3075) with most of the remaining studies with sample sizes usually of 200 to 400 (but with up to 800 subjects, median = 387). Ethnicity was generally not reported explicitly, although two studies reported on African Americans ("Blacks") and Caucasians ("Whites"), one was of Japanese Americans and one of Mexican Americans. Data were obtained from studies from a variety of countries and regions including the USA (21), Finland (3), Denmark (2), UK (2), Canada (1), Taiwan (1), Israel (1), and a combination of countries (Finland, Italy and the Netherlands, n = 2; Europe, n = 1; and USA with England, n = 1).

Age groups studied included samples: of "younger" age, starting the study in their 6^th ^decade with follow-up generally at a mean age of 65 to 70 years (11 studies); "older" groups initially aged 60 to 70 years and followed-up in their mid-seventies (13 studies); and, "oldest" groups aged 70 years and up with many followed into their late-seventies and older (5 studies); and the other studies surveyed a very broad age range. Most studies included both men and women, but a few were of men only (n = 7) or women only (n = 1).

The "intervention" of interest was physical activity. Physical activity reports were almost all from self-report questionnaires (with one study also having an objective assessment of physical activity, and two studies also measuring cardiorespiratory fitness). About one-half of the studies used "walking as exercise" and various other activities described as "vigorous" as those "counting" toward being physically active (mostly in samples of younger baseline age). Thus, it was possible from studies to characterize the variety of aerobic activities as being of light, moderate or vigorous intensity. About one-third of the studies used some assessment of total score for a breadth of activities, with a few focused mainly on assessing frequency and distance of walking as the main activity (usually in older groups), but in general a distinction between frequency and duration was not clear and thus only total "volume" of activity could be characterized. In six [14-19] reports the "exercise group" were members of a jogging club (over the long-term) or long-term adherents to exercise programmes, compared with a reference group. In general physical activity level was categorized into groups with the studies using two groups (n = 9) or three groups (often tertiles, n = 9) or more groups or a continuous variable (n = 8). In a small group of studies physical activity assessment was made on more than one occasion, thus allowing groups to be formed to assess change in activity level (e.g. became active, or became inactive, or always active or inactive) over time [14,20,21]. For three studies the report was on the association of strength with functional outcomes [22-24].

Outcomes of impairment or functional limitations, or functional status decline, or disability were all considered. Across the studies a very wide range of measures (from disability to self-report difficulties in tasks like walking 400 m or taking a flight of stairs, and to performance tests involving walking speed, chair rises or carrying a load) were used to assess these functional independence outcomes. The outcome measures were mostly by self-report questionnaires. About one-half (n = 19) of the studies used functional status questionnaires of abilities (or level of difficulty) in activities of daily living (ADLs) and instrumental ADLs (i.e. IADL) or the Health Assessment Questionnaire for Disabilities (HAQ-D1). Other categorizations of similar low level functional capacity or dependence included self-report of functional status and "quality of life" questionnaires (including the Medical Outcomes "Short-form 36" scale, SF36). In a few studies the report of a mobility limitation was also used as the outcome. Eleven of the studies reviewed used functional limitations or reported performance abilities at a much higher level usually in the younger samples. These assessed physical performances such as ability in stair climbing or walking a distance, or tasks involving both the upper and lower extremity (e.g., Huang et al.[25]), and five studies reported on both ADL-type measures as well as higher level performances. Only a very few studies used a measured physical performance (a physical performance test battery, and/or short distance gait speed). "Performance" measures used as the outcome included walking speed or chair rises, with a common proxy of mobility disability relating to various tests of walking such as the 400 m walk.

The period of follow-up ranged from 2 years up to 35 years with many in the 5 to 10 year range (median follow-up 7 years), and included one-time follow-up (n = 22), but also follow-up at multiple time points (two to four time points, or in some cases annual evaluations), (n = 13). In analyses of the relationship of physical activity and functional outcomes in all papers there were statistical adjustments for confounding variables.

##### Results, Data

Greater physical activity (categorized by various methods) predicted higher functional status (expressed in a variety of ways) in older age. To facilitate analysis of these studies they have been grouped into those with an outcome related to disability, and those with an outcome related to a higher level of functions such as functional limitation.

**Figure 2:**
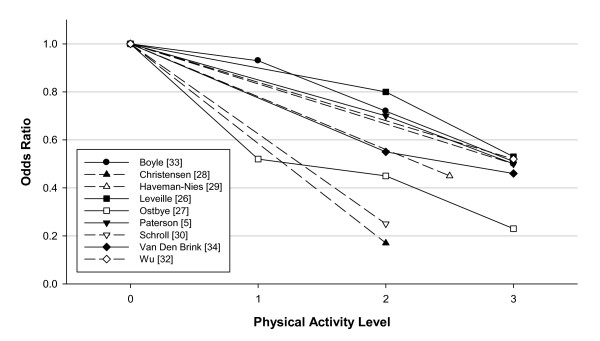
**Prospective cohort studies of the odds ratio of functional limitations or disability in ADLs and IADLs or quality of life disability indexes in relation to physical activity level**. The odds ratio (OR) for each level (1,2,3) of physical activity are compared with the lowest physical activity group assigned as the referent (physical activity group = 0, OR = 1). The odds ratios are those reported for analyses statistically adjusted for confounder variables. The OR is an approximation as the actual data may have been separated into men versus women, or other groupings and thus in assembling the data these values have been combined. The level of physical activity was determined from analysis of types of activities that were reported for each activity level in each study; for example, in a number of studies there were only two activity groups but to be in the higher group there had to be report of vigorous activities or walking of 1 hour per day. Thus the physical activity groups were determined to require: 3 - vigorous activities and/or high volume of systematic activity (walking for exercise); 2 - moderate level of activity from participation in normal walking or gardening with a volume of 3-5 days/week and 30 min per day; 1 - participation mainly in light activities of daily living with only occasional walking or gardening; and some physical activity groups were scored as 2.5 as they included vigorous activity (not included in group 2) or exercise walking but at a lower volume than group 3. Dashed lines indicate study data where only two activity groups were categorized, whereas solid lines join the data points for studies in which more than two activity groups were formed.

In overview, with regard to an outcome of "disability" in ADLs or IADLs, or a level of disability, studies have consistently shown a reduced risk in the more physically active. A meta-analysis of the data was considered, however, heterogeneity in both the categorization of physical activity levels and in the functional outcome measures precluded pooling the study results. Rather, Figure 2 depicts that with higher levels of physical activity there is a reduction in risk for various outcomes related to functional limitations or disability with an "average" odds ratio (OR) of ~0.5. There is also the suggestion that a "moderate" physical activity level is also effective in preventing functional limitations and disability. Although two studies [26,27] showed a significant trend across activity levels only one study [27] suggested a significant effect for a light or low physical activity group. Thus, it appears that moderate to high levels of physical activity are effective, but based on current literature there is limited justification for recommending the pursuit of merely a low level of physical activity. Many of the studies emphasized in their physical activity assessment the amount of walking, but wherein the duration or total walking (or the relative intensity) to qualify in the higher versus lower activity groups most often implied an "intention to exercise" versus accumulating daily activities of various sorts. Thus, although from the present data and in particular the variety of ways in which physical activity was categorized, a specification of a minimal amount (or intensity) of physical activity needed for a reduction in risk of functional outcomes is not feasible, Figure 2 does suggest a 50% reduction in risk of functional limitation/disability is possible with physical activity categorized as moderate to higher levels in total amount and at least of moderate sustained intensity.

It is instructive to examine some of the individual studies to determine the physical activity categorizations that resulted in reduced risk of functional disability. Christensen et al. [28] reported being active versus sedentary at age 70 years had an OR for disability at age 75 years of 0.17. Haveman-Nies et al. [29] found for the intermediate and high-active versus low-active the risk of functional dependence in ADLs was 0.53 in men and 0.38 in women. Schroll et al. [30] summarized studies in a Danish population; in older age the OR for mobility dependence was ~0.25 in physically active (approximately 20 min/day or 2-3 hours/week) versus sedentary. Unger et al. [31] showed greater frequency of walking was related to lower levels of impairment and functional decline. In Wu et al. [32] those who participated in "routine exercise" had an OR of 0.52 for subsequent ADL disability.

From five studies (see Figure 2) it is possible to analyse dose-response relationships between different physical activity levels and a disability outcome. Boyle et al. [33] noted a 7% reduction in disability risk for each hour of walking (and/or other exercise), or expressed another way a 40-50% reduction in risk with 1 hour of such activities daily (7 to 8 hours per week). Leveille et al. [26], for the probability of disability versus being free from ADL disability prior to death, expressed a 0.53 odds ratio in those of higher levels of physical activity, but not for intermediate activity levels, versus the low activity group; nevertheless, the statistical trend across categories of physical activity was significant. Østbye et al. [27] reported the OR for ADL disability or dependence was more linearly related to activity level from light with OR ~0.5, through moderate with OR ~0.4, to vigorous OR of ~0.2, with the statistical test of trend across physical activity groups again significant. From Van Den Brink et al. [34] total physical activity in walking cycling and gardening reduced risk of disability with OR in the range of 0.6 for the middle-tertile and 0.4 for the highest-tertile compared to lowest, with duration of these activities (up to 100 min per day) being the important criteria. Paterson et al. [5] reported the OR for those living independently becoming dependent in follow-up in relation to their cardiorespiratory fitness and demonstrated a ~30% reduction in those of moderate and ~50% reduction in those of higher fitness versus the low fit group; however in this study the reported physical activity levels among the older groups were not related to subsequent dependence.

There were two studies that had negative findings in terms of supporting the relationship of greater physical activity with functional limitations or disability. Hirvensalo et al. [35] noted that for those with intact mobility the risk of dependency did not differ between active and sedentary. Wannamethee et al. [21] found that the inverse relationship between physical activity and mobility limitation showed a trend, but was not significant, when the odds ratio of moderately vigorous activity related to mobility limitation was adjusted for the presence of chronic disease.

**Figure 3:**
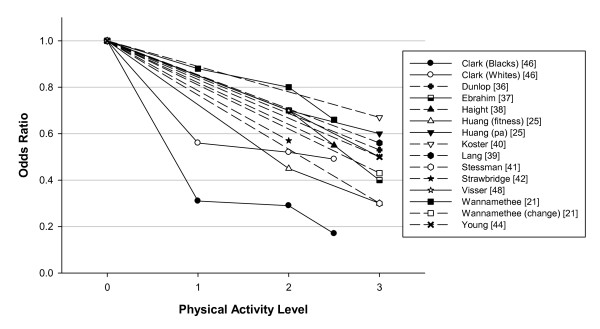
**Prospective cohort studies of odds ratio of lower functional performance or functional limitations in "higher" level functions (such as walking a distance or climbing stairs) in relation to physical activity level**. The odds ratio (OR) for each level (1,2,3) of physical activity are compared with the lowest physical activity group assigned as the referent (physical activity group = 0, OR = 1). The odds ratios are those reported for analyses statistically adjusted for confounder variables. The OR is an approximation as the actual data may have been separated into men versus women, or other groupings and thus in assembling the data these values have been combined. The level of physical activity was determined from analysis of types of activities that were reported for each activity level in each study; for example, in a number of studies there were only two activity groups but to be in the higher group there had to be report of vigorous activities or walking of 1 hour per day. Thus the physical activity groups were determined to require: 3 - vigorous activities and/or high volume of systematic activity (walking for exercise); 2 - moderate level of activity from participation in normal walking or gardening with a volume of 3-5 days/week and 30 min per day; 1 - participation mainly in light activities of daily living with only occasional walking or gardening; and some physical activity groups were scored as 2.5 as they included vigorous activity (not included in group 2) or exercise walking but at a lower volume than group 3. Dashed lines indicate study data where only two activity groups were categorized, whereas solid lines join the data points for studies in which more than two activity groups were formed.

In study samples at the "higher" function level the relative risk or odds of functional decline or limitation was significantly reduced in those more physically active, usually defined by regular, vigorous activities. Again a meta-analysis was not possible due to heterogeneity in the physical activity categorizations and in the outcome measures. Nevertheless, Figure 3 depicts in these studies the risk of a functional limitation outcome was again ~50% reduced in the high active group, and also a reduced odds ratio was evident in the moderately physically active group. Although a few studies (see below) showed a dose-response relationship only two studies actually had a light physical activity group to compare to a sedentary group and thus to date there is not enough data or consistency to support a recommendation of light activity. Thus, in overview in preventing decline or limitations in "higher" levels of performance the comparisons have often revealed that the physical activity is "exercise", or "vigorous", but there is some evidence for "moderate" activity as well (e.g., a "middle" moderate-activity group versus sedentary group).

Again it is instructive to attempt to discern the levels of physical activity that were protective. The studies of Dunlop et al. [36], Ebrahim et al. [37] (and follow-up report by Wannamethee et al. [21], Haight et al. [38] and Lang et al. [39] each reported that "vigorous" exercise (3 times/week of greater than 30 min) or as one stated "exercise at high aerobic levels" limited functional decline and functional limitation in "high level" physical functioning tasks with odds ratios consistently close to 0.5. Huang et al. [25] also assessed higher level physical functions and reported an OR for prevalence of functional limitation of ~0.4 for those of moderate and high fitness versus lower fitness, and an OR of ~0.7 for moderate and high versus low physical activity. Koster et al. [40] reported the protective effect of physical activity (particularly for the high versus low activity groups) on incident mobility limitation across different adiposity levels and in overview the OR was ~0.6. Stessman et al. [41] found an OR of 0.2 to 0.4 for continued "ease" of performance of ADLs and IADLs in the group "exercising" at least 4 times/week. Strawbridge et al. [42] reported an OR of 0.57 for various descriptors of loss of function in those who reported "often walking for exercise" versus those who did not report walking for exercise. Wang et al. [43] included performance-based physical function measures and noted that those who exercised 3 times/week or more had better functional outcomes and decreased rates of functional decline. Young et al. [44] found those who were high versus low active on total activity level maintained optimal function for basic ADL with an OR of 0.43, and for physical endurance type tasks an OR of 0.59 versus the low active subjects (and in those with a chronic disease at least moderate physical activity appeared sufficient to maintain physical functioning).

The study of Wannamethee et al. [21] is of importance as it examined the effect of change in physical activity level in older age. Wannamethee et al. [21] noted in the moderately vigorous physically active group the OR of a mobility limitation was 0.77, compared to no vigorous activity, and maintaining or taking up physical activity was associated with less mobility limitation such that becoming active yielded an OR of 0.43 for mobility limitation versus remaining inactive.

Seven of the studies shed light on the dose-response relationship (with odds ratios available from 5 of these studies and plotted on Figure 3). Brach et al. [20,45] noted that being consistently active (defined as 30 min/day moderate physical activity, on most days, 1000 kcal/week; 4200 kJ/week) was associated with better physical function. Clark et al. [46] found that walking 4 to 7 days/week reduced the onset of disability by 50 to 80% (OR 0.5 to 0.2). In both Brach et al. [20] and Clark et al. [46] there were trends across lower levels of physical activity (e.g., walking 1 mile 2 times/week) or with some of the functional outcomes. Ferrucci et al. [47] expressed results of an increased active life expectancy of about 4 years with a 1 to 2 year compression of morbidity in the more active compared to the lower quartile of activity, with incremental benefits in the moderate (2 quartiles) to upper (top quartile) activity groupings. Ebrahim et al. [37] reported an OR for locomotor disability of 0.7 in the occasional or light active and 0.4 in the moderate to moderately-vigorous group, compared to the sedentary group, although from these data Wannamethee et al. [21] reported the trend (after adjustment for chronic disease) was marginally non-significant. Huang et al. [25] reported significant linear trends across both activity and fitness categories. From Visser et al. [48] in those meeting 30 min of moderate activity on most days the hazard ratio (HR) of mobility functioning was ~0.5 versus the inactive, and in those who were "life-style active" but did not meet the "exercise group criteria" the risk compared to the inactive was intermediate at ~0.7; also noted was the importance of walking and expending 400 kcal/week (1680 kJ/week) in walking versus the inactive which yielded a HR of ~0.6.

The longitudinal data also include six studies [14-19] wherein analyses are done for older adults who were committed "joggers" or long-term exercisers. In these studies the jogging groups or those exercising at high levels in middle-age and later clearly postponed a disability or functional limitations and prolonged disability-free life. However, it does not seem feasible to extrapolate these findings in an exceptionally active group to determine useful interventions for the general older population.

Three studies related hand grip strength to subsequent disability. With greater hand grip strength there was a decrease in functional limitations and disability in ADLs [22-24]. These studies show a relationship with strength but do not provide the evidence that regular, strength-related activities are associated with reduced risk of functional limitations. Buchman et al. [49] reported that both physical activity and leg strength were independent predictors of mobility decline in older persons (age 80 years). Schroll et al. [30] reported that an increase in leg muscle strength over a 5-year period in women aged 75 years was related to mortality (but not to functional outcomes) and the strength factor was not significant for men. Nevertheless a dose- response relationship was reported for declining muscle mass in relation to functional limitations in both men and women.

##### Interpretation

Overall it can be concluded from prospective studies that regular physical activity (in "aerobic" activities) in middle-aged and older adults confers a reduced risk of functional limitations and disability in older age. The reduction in risk for the effect of physical activity on a variety of outcomes related to function most often appears to be in the range of 30 to 50%.

A modified scale [10] for the evaluation of quality of the prospective studies was employed (with the following items omitted: items 4, 5 and 8 in the reporting scale; items 12, 13 in the external validity section; 14, 15, and 19 in the section on bias; items 21-26 relating to confounding; and item 27 addressing power). The final checklist was made up of 12 items with a maximum score of 12 points (with higher points indicating superior quality) rather than the original 32 points. The studies examined were of good quality scoring 8 to 12 (median 9) on the modified Downs and Black scale (Table s5, see additional file 3).

The strength of the recommendations is related to a consistency in the findings across studies. This strength of evidence is underscored by the fact that there is a consistency of the effect of physical activity across a very wide range of outcome measures from disability in ADL or scores on quality of life or the SF36, to self-report difficulties in tasks like walking 400 m or taking a flight of stairs, and to performance tests involving walking speed, chair rises or carrying a load. Furthermore the effect of physical activity has been demonstrated with short-term follow-up of a couple of years as well as for long-term follow-up. In the former there is some concern that the physical activity groups formed initially may have differed in undetected ways in "health status" and the physical activity effect was exaggerated; however, in the long-term follow-up it is likely that the effect of physical activity is underestimated in that in the initially designated active group it is likely that many became much less active over the long-term follow-up and thus should have been in the inactive group. Nevertheless, an additional strength of the data comes from studies that assessed physical activity at different time points and have shown that being consistently active was associated with a reduced risk of functional limitations, a perhaps of even greater importance that becoming physically active (from inactive in older age) similarly reduced risk of functional limitations. A further reassurance is the evidence of a dose-response relationship of physical activity level with the various outcomes of function.

The included studies have some limitations. A clear weakness in the data available to date is in the limitations of the physical activity assessments. Most studies have used just two categories of physical activity, sedentary versus active, but there is a large variation in how these categorizations were derived from the active being defined as those who participated in a grouping of vigorous activities, to defining the active as the middle-tertile or even middle two quartiles of the physical activity "scores" of the sample. It is not possible to discern for most categorizations when a "high" or "moderate" physical activity score was related to intensity of activities undertaken or a score from total volume of activities. Thus, whereas there appears to be a dose-response relationship with physical activity and functional outcomes the expression of this "dose" in terms of a recommendation is not precise. Particularly, whereas there is data to show that "higher" levels of physical activity are beneficial there is not the data to support that "lower" physical activity levels are effective and thus there is need to establish whether there is a minimal threshold of physical activity for benefit. In this aspect we reviewed only published literature and there is the possible publication bias of rejection of studies that did not show significant results, which would be more likely if studies had compared light activity groups to sedentary rather than comparisons of heavy or at least mid-range activity groups to the inactive group. This bias in publication (that non-significant comparisons for light activity groups that may have been found would not get published) underscores the caution in recommending activities of less than moderate intensity and volume.

Given the above analyses, there appears to be a dose-response effect that more activity has a greater effect but precise categorization of a minimal or effective or substantive dose is not clear cut. Critical evaluation and analysis of each of the studies was required to attempt to discern, or speculate, regarding any of the physical activity details of type and mode, and duration or intensity of the activity in relation to outcomes. The studies with outcomes related to ADLs and disability, generally in older age groups (baseline age >70-75 years), often assessed activity level by participation in walking (>1 mile on each session, or "for exercise"), gardening and exercising. Thus, for individuals of these age groups, those classified as physically active were doing some moderately-vigorous activities and one would conclude that the requisite physical activity level was high with conclusions such as walking 1 hour/day, frequently walking > 1 mile, walking for exercise 20 min/day 3 times/week, walking and gardening 100 min/day, optimally; nevertheless others did find dose-response trends suggestive that the middle-tertile of physical activity groups also had reduced risk of ADL disability. In older groups (>70-75 years of age) frequently walking 1 mile or more appears effective in preventing disability. Thus, for older groups (>70-75 years of age) the prevention of functional losses and limitations is met through physical activity levels equivalent to recommendations of walking or other activities 5 times/week for 30 min (or 150 min/week) or more vigorous exercise walking of minimally 60 min/week (20 min, 3 times/week), although the dose-response data suggest that some lower levels of physical activity may also have influence in this "older" group.

With regard to studies where the outcome was functional decline or functional limitation or ability to perform or performance on "higher" level tasks, in six studies [21,36-39,41] in sample groups usually younger (60 to 70 years of age at baseline) the active group were defined by a rather vigorous or a "high" level of activity and in another group of studies with sample ages usually in their 70s the protective activity was at least moderate (and usually >4 times/week) [16,20,42,44-46,48]. Nevertheless in a number of studies there was a dose-response relationship of physical activity level (usually inactive, to moderate, to high) with the outcome [20,25,40,45-48]. Thus, moderate to higher levels of activity of at least 4 times/week or 180 min/week are effective; although there may be an inverse dose-response relationship of physical activity level with functional limitations and disability there remains the possibility that there is a minimal threshold of at least a "moderate" activity level for a significant effect on outcomes. For younger groups (generally age 60-70 years) the activities proven effective were vigorous or at a high level, although again there was a noted dose-response between moderate and heavy in a couple of these studies [20,25,45].

Beyond the aspects of intensity or duration, or total energy expenditure, as Keysor [50] point out, it remains unknown as to what aspects of physical activity (or exercise) behaviours are important in terms of disablement outcomes, or for that matter functional outcomes. Thus as well as conventional physical activities or exercise, multi-factorial activities that include balance, endurance, trunk rotation, transfers, weight shift transitions and strengthening may be effective as they relate directly to specific functions or disabilities, but for the present only recommendations of aerobic activities are supported by the data. In this regard the physical activity of walking seems ideal in direct relation to daily functions of older individuals and prevention of mobility disability. Nevertheless, a host of aerobic activities are suitable including other recreational activities (e.g., cycling), sport participation (of an aerobic nature, e.g., squash, soccer, basketball, ice-hockey), or household chores of an aerobic nature (e.g., mowing the lawn).

##### Recommendations

The prospective cohort studies provide evidence that a 50% reduction in risk of functional limitations and disability is possible in more physically active older individuals. The evidence of this relationship is Level 2, Grade A. It appears that this degree of protective effect requires physical activity of moderate to higher levels (or moderate to high cardiorespiratory levels), again supported by Level 2, Grade A evidence. The dose descriptors equate moderate to higher levels of physical activity as about 30 to 60 min/day or totalling about 150 to 180 min/week (and approximating 1000 kcal/week; 4200 kJ/week) with intensity descriptors of moderately vigorous to vigorous or "walking for exercise" (Level 2, Grade A). Although there is a dose-response trend across at least moderate to high activity levels, a recommendation of lower level (light) physical activity lacks evidence and may be ineffective (i.e., a recommendation of light activity would be at Level 3 or 4, and Grade B). At present, based on prospective cohort studies, only recommendations of aerobic physical activities are supported by the data, as there is little data regarding the relationship between strength-related activities and reduced risk of functional limitations (although there is evidence that these may be recommended in persons with clinical conditions, functional limitations, disability or frailty of old age). Thus, from prospective studies a recommendation of strength-related activities would be Level 3 or 4 and Grade B.

#### Cross-sectional and retrospective cohort studies of physical activity and functional limitations in older adults

Information from cross-sectional observational studies linking physical activity levels to functional measures was reviewed but a complete systematic review and tabulation of the papers was not done as these studies show relationships of physical activity to functions, but it is usually unclear as to whether a functional limitation preceded or was consequent to a lower physical activity level. An important study relating physical activity and functional outcomes is that of Morey et al. [51]. In this study of 161 men and women of mean age 72 years it was shown that both lower cardiorespiratory fitness and muscle strength were associated with functional deficits (from self-reported functional abilities and a performance test) and thus lack of "fitness" was a critical modifier on the path to disability. Among the cross-sectional studies four that were reviewed had large subject samples (>2000 subjects). Brach et al. [45] for subjects over age 70 years found that exercisers compared to life-style active and inactive scored higher on the performance test battery and a 400 m walk. Brown et al. [52,53] found that subjects age >65 years with physical activity levels of 3-4 days/week or 5-6 days/week reported fewer "unhealthy days" than the inactive group (although 7 days/week and extensive vigorous exercise were negative in that "too much" predicted more unhealthy days). Simoes et al. [54] in subjects >60 years of age found a dose-response relationship of physical activity to level of ADL and IADL disability with the active group 45 to 60% less likely to report a disability. Sulander et al. [55] examined a number of health behaviours and noted physical activity associated with poorer ADL functions in subjects aged 65 to 79 years.

Other studies reported relationships of moderate physical activity with SF36 scores [56]; relatively high physical activity significantly related to lower ADL or IADL disability [57]; and a better functional status index in those who "exercised" even a minimal amount [58]. Others related fitness measures to functional performance with VO_2_max related to functional limitations [59]; aerobic exercise capacity explaining 25% of the variance of physical function on the SF36 questionnaire [60]; VO_2_max, but not strength, being related to SF36 scores [61]; and VO_2_max relating to ability to perform functional tests and ADL activities [62]. Additionally, cross-sectional analyses have related strength measures to various functional outcomes [59,62-65]. Overall, the cross-sectional information relates physical activity or aerobic or strength fitness to "better" functional measures, but the studies essentially do not add to the determination of recommended physical activity levels.

#### Exercise training studies in older adults with functional outcomes: Aerobic programmes and "combined" programmes

##### Description of Studies (population, intervention, outcome)

This systematic review also examined the influence of exercise training programmes on functional outcomes. Exercise training intervention studies, of either aerobic or aerobic supplemented with resistance training, are summarized in Table s6 (see additional file 4). A total of 14 articles (14 unique data sets, and 3 supplementary reports) met the criteria for inclusion in the evaluation of the effects of aerobic training programmes on indicators of functional status in older adults. This included 1,938 unique participants with study sizes ranging from 13 to 582 (median n = 114). Five studies were of female samples only, with the remaining including both men and women. The median training duration of the studies (excluding a study that used a 10-year telephone based follow-up [66]) was 24 weeks. Ethnicity was generally not reported explicitly. However, data were obtained from studies from a variety of countries and regions including the USA (8), Canada (2), Japan (2), Turkey (1), and Greece (1). The articles were published between 1996 and 2009.

##### Results, Data

From exercise training programmes the functional outcomes include changes in physical performance functions such as walking speed/distance, or self-report functional abilities such as physical function assessed by the SF36. To date these studies have used proximal outcomes taken at the end of the training programme; but, the effects on distal outcomes, whether impairments and functional limitations, or disability are not as apparent (Table s6, see additional file 4). Nonetheless, the literature was consistent in supporting the reduction in the risk for functional impairments and limitations in older persons after aerobic or combined exercise training.

The pilot data reported by Pahor et al. [67] is perhaps most important to date. A combined exercise programme for ~400 men and women of mean age 77 years reported data up to 12 months. The physical activity programme resulted in higher physical performance scores; and this study used the 400 m walk as an indicator for mobility disability finding that there was a lower incidence of the inability to complete the walk in the physical activity group versus an educational control group, with an odds ratio of 0.71 approaching significance in this pilot data. Thus, this study has examined the proximal outcomes of a 12 month programme, but with a measure that is an indicator of disability rather than just functional measures.

##### Interpretation

The quality of the studies reviewed is summarized in Table s7 (see additional file 5). For RCT studies, a modified scale was employed (with the following items omitted: item 13 in the external validity section; 14 in the section on bias; item 24 relating to confounding; and item 27 addressing power). The final checklist was made up of 23 items with a total score of 24. For non-RCT studies, a modified scale was employed (with the following items omitted: item 13 in the external validity section; 14 in the section on bias; items 23 and 24 relating to confounding; and item 27 addressing power). The final checklist was made up of 22 items with a total score of 23. The median score of the 10 RCT studies was 21 of a possible 24, thus the studies were of good quality. For the four non-RCT studies the median was 17.5 of a possible 23.

It is not possible to clearly define the minimal volume and intensity of exercise training required to elicit the improvements in functional status owing to the variability in the methodologies employed and the lack of descriptive information regarding the volume of activity in many studies. Indeed the majority of the studies employed moderate intensity aerobic exercise for 3 days/week and generally 30 to 45 min/session.

Thus, in overview, training interventions of aerobic exercise and walking programmes are effective in improving functional abilities. Additionally there are a large number of studies measuring an improved physiological outcome (VO_2_max) with training, but not a functional outcome (see [2]). The exercise training studies do demonstrate that a short-term exercise programme in older adults is effective, and thus add to the information from prospective follow-up studies wherein a physically active lifestyle is presumed to have been a characteristic of an individual over the long-term and thus implicating that lifelong activity was needed. There is some expressed concern that a physiological improvement or an improved score on functional abilities may not translate to prevention of physiological limitations or future disability; however, the link between physiological and functional limitations has been established in the cross-sectional studies. And the prospective cohort study of Paterson et al. [5] showed a relationship of VO_2_max with subsequent dependent living.

##### Recommendations

Thus, the prospective studies provided evidence regarding a long-term lifestyle of physical activity, whereas these exercise training interventions add that a short-term physical activity intervention (exercise programme of moderate intensity, 3 times/week) is also effective (and adds to prospective study conclusions) in reducing functional impairment or limitation with Level 2, Grade A evidence. It is also notable that functional outcomes were more greatly affected using combined aerobic and muscle resistance exercise programmes [68,69]. Thus resistance training of older adults appears to complement aerobic physical activity benefits, and could be a recommended adjunct to a physical activity programme, but at Level 3, Grade B.

#### Exercise training studies in older adults with functional outcomes: Resistance training programmes

##### Description of Studies (population, intervention, outcome)

Exercise training intervention studies of programmes of resistance training (or muscle function training) are summarized in Table s8 (see additional file 6). A total of 17 articles met the criteria for inclusion in the evaluation of the effects of resistance training programmes on indicators of functional status in older adults (Table s8, see additional file 6). This included 845 unique participants with study sizes ranging from 10 to 124 (median n = 40), and 11 studies included both males and females with six of females only. The average training duration of the studies (excluding a study that used a 7.7 year follow-up [70] was approximately 24 weeks (ranging from 6 to 72 weeks). Ethnicity was generally not reported explicitly. However, data were obtained from studies from a variety of countries and regions including the USA (9), UK (3), Canada (1), Chile (1), Italy (1), Austria (1), and South Africa (1). The articles were published over a 12 year period ranging from 1995 to 2007.

##### Results, Data

Similar to the aerobic training literature, the effects of resistance training programmes on disability are not as apparent as the more immediate measures of effects on functional impairments and limitations. In general the subject groups were of mean age between 70 and 80 years, and most studies of 6 to 24 weeks showed changes in strength or muscle power in various muscle groups. With regard to functional outcomes, there was some compelling evidence that moderate intensity resistance training was effective in improving functional status in older adults. Five studies [71-75] showed that with training programmes ranging from 6 weeks to one year, and resistance training programmes that ranged from moderate intensity (50% of 1 RM (repetition maximum) or moderate weights allowing 12-15 repetitions), there were improvements on a number of functional abilities [71] or specific functions such as a chair rise test [74] or stair climb [75]. Nevertheless four studies [68,76] (reported on Table s4, see additional file 2); [73,77] each of greater than 16 weeks and relatively heavy resistance training programmes showed adequate strength gains but marginal or no improvement in various functional performances. Other studies listed in Table s8 (see additional file 6) also did not clearly demonstrate substantial functional changes.

##### Interpretation

As reviewed in Table s9 (see additional file 7) the resistance training studies were on average of fair to good quality with RCT studies scoring a median of 17/24 and non-RCT 15/23. (As per the aerobic table, for RCT studies, a modified scale was employed with the following items omitted: item 13 in the external validity section; 14 in the section on bias; item 24 relating to confounding; and item 27 addressing power. The final checklist was made up of 23 items with a total score of 24. For non-RCT studies, a modified scale was employed with the following items omitted: item 13 in the external validity section; 14 in the section on bias; items 23 and 24 relating to confounding; and item 27 addressing power. The final checklist was made up of 22 items with a total score of 23.)

However, in summary, as in a detailed in a review by Latham et al. [78], although progressive resistance interventions in older people yield increases in strength and can have a positive effect on some functional limitations, the evidence of improved functional performances is controversial, and the effect on substantive outcomes of disability or aspects of health are not clear. Resistance training alone (i.e., without the aerobic training recommendations) does not seem to be a supportable recommendation for functional outcomes, or for morbidity or mortality in older adults (although a greater muscle mass may be associated with reduced mortality reportedly secondary to a reduced risk of type 2 diabetes).

##### Recommendations

Thus, progressive resistance strength programmes may yield improvements in some selected functional tasks but to date it cannot be concluded that these will result in reduced functional limitations or disability. If a recommendation of resistance strength programmes based on the evidence from these training interventions were to be made it would be at Level 3, Grade B or C.

#### Mechanisms of the relationship of physical activity and functional limitations

There are plausible mechanisms to explain a relationship between physical activity and lower risk of functional limitations. Randomised control trials of exercise programmes show improvements in physiologic capacity related to aerobic fitness, muscle strength and power and also improved functional abilities. Lang et al. [39] suggested a number of factors: i) physical activity may relate specifically to physical function; for example muscle strength may have a mediating role between physical activity and disability; ii) physical activity is protective against the metabolic syndrome thus reducing incidence of conditions whose consequences include reduced physical function; iii) exercise and physical activity are associated with lower inflammatory markers in older adults and may reduce the damaging effects of inflammation, including those associated with excess adipose tissue; iv) physical activity provides psychological benefits; v) physical activity may maintain body weight and strength (and mitigate against age-related loss of lean body mass).

Boyle et al. [33] also suggested mechanisms that may underlie the complex association between physical activity and disability. They included the effects of: improved aerobic capacity, muscle strength, and flexibility; protection against development and progression of disabling conditions (diseases such as cardiovascular, respiratory, osteoporosis, as well as nerve growth factors relating to cognitive function and protection against ischemic and neurotoxic damage); and, favourable psychological effects.

As noted earlier, a variety of recreational, sport or household activities may be appropriate for aerobic exercise. And as a caveat to recommended physical activities regarding sex difference - in older women exercise training yields minimal change in heart stroke volume (central adaptation, see [79]); thus specificity of exercise programming to the muscle groups used in mobility of daily activities is important. Walking is of course specific, and an activity such as cycling would probably have carry-over to aerobic adaptations in muscle groups used in mobility of daily activities, whereas swimming (arm exercise) lacks the specificity and would not be a recommended activity for older women wishing to gain aerobic fitness for accomplishing daily functions.

### Cognitive Function

#### Search Results: Physical Activity and Cognitive Function

**Figure 4:**
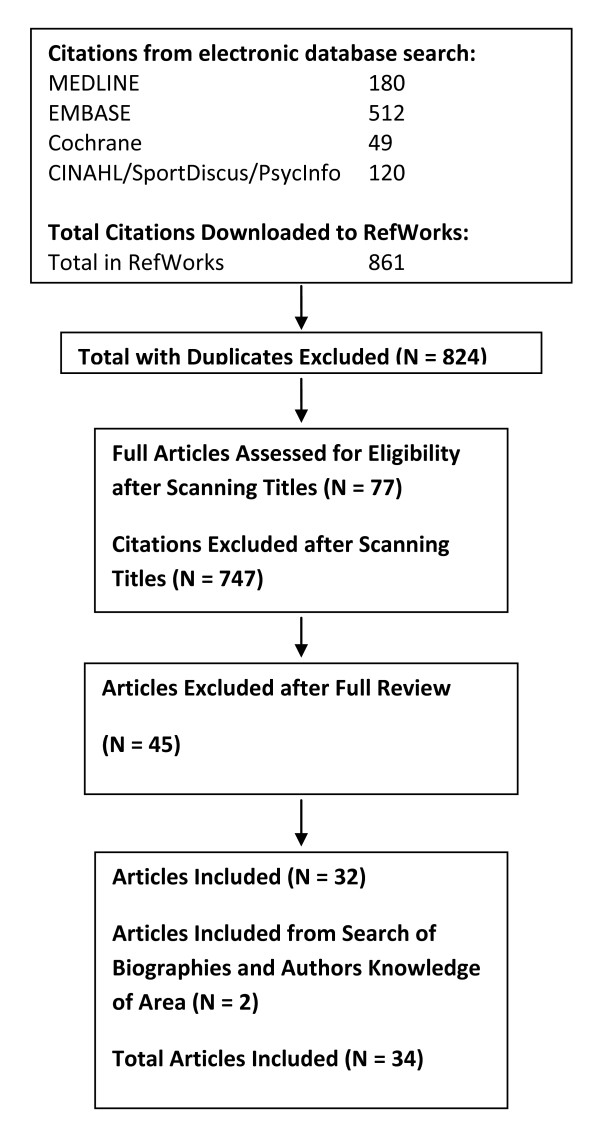
**Results of the literature search for cognitive function**.

A total of 861 citations were identified during the electronic database search (Figure 4). Of these citations, 180 were identified in MEDLINE, 512 in EMBASE, 49 in Cochrane, and 120 in the CINAHL/SportDiscus/PsychInfo search. A total of 37 duplicates were found, leaving a total of 824 unique citations. A total of 747 articles were excluded after scanning, leaving a total of 77 articles for full review. From these articles 45 were excluded after full review leaving 32 articles for inclusion in the systematic review. The reasons for exclusion included: participant group did not meet the inclusion criteria for age, absence of disease (i.e., study was of a clinical population) or for life-style (community-dwelling); functional measure not reported; physical activity level or exercise capacity not reported; the citation was a review, dissertation, thesis, or abstract.

A total of 32 articles met the criteria for inclusion in the systematic review of the literature regarding the relationship between physical activity and cognitive function in older adults (Table s10, see additional file 8). Additional literature was tracked from reference citations and author files. These searches provided an additional 2 citations. Therefore, a total of 34 articles were included in the systematic review of the literature regarding the relationship between physical activity and cognitive function.

##### Description of Studies (population, intervention, outcome)

The systematic review captured articles analysing associations between physical activity or cardiorespiratory or muscle fitness and a cognitive function outcome and included prospective cohort studies, cross-sectional studies, and exercise training intervention studies. These studies included 19,988 participants with study sizes ranging from 14 to 4,615 participants. Ethnicity was generally not reported explicitly. However, data was obtained from studies from a variety of countries and regions including the USA (15), Australia (6), Canada (1), Turkey (1), France (1), Brazil (1), UK (2), Sweden (1), Japan (1), Greece (1), Italy (1), Nigeria (1), Netherlands (1), and a combination of countries (Finland, Italy and the Netherlands). The articles were published over an 18 year period ranging from 1989 to 2007.

The "intervention" of interest was physical activity. Physical activity reports were almost all from self-report questionnaires, and a few studies used cardiorespiratory fitness measures, or strength measures, to relate to cognitive function. The physical activity descriptors used total amount of activity, categories of frequency of participation, or intensity of activities to categorize groups.

There was considerable variability in the measures employed to evaluate cognitive function, which made the interpretation of the findings difficult. In fact, there was no consistent battery of tests to evaluate cognitive function. Measures may have included cognitive speed (e.g. simple reaction time, choice reaction time), visual memory (e.g. Wechsler Memory Scales visual reproduction, Benton visual retention test), verbal memory (e.g. Randt memory test, Weschler memory scale), motor function (finger tapping), working memory (e.g. digit span tests), perception (face recognition), executive functions (e.g. verbal fluency, problem solving, word comparison), cognitive inhibition (e.g. Stroop test), visual attention (e.g. letter search, visual search), and auditory attention (e.g. Digit span forward).

##### Results, Data

The majority of the articles (24 (71%)) demonstrated a positive relationship between physical activity/fitness and indices of cognitive function.

Nine studies provided prospective "follow-up" cohort data with 7 (78%) of these demonstrating a positive relationship between the physical activity level and cognitive function outcomes. The two negative studies did not find a relationship to cognitive performance or impairment related the amount of walking or sports [80] and a continuous 0-70 point activity scale [81]. The positive studies had physical activity descriptors of physical activity or not [82], walking greater than 2 miles per day (versus less than 1 mile [83]), engaging in a number of activities [84], or various activities more versus less than 3 times per week [85], or for less than 30 up to greater than 120 min/day [86], and high intensity versus low or moderate intensity [87], or grouped by fitness tertile [88]. The positive outcomes ranged from better cognitive function, to less likely to develop dementia or Alzheimer's disease, or a delayed onset of dementia. In overview from these studies it would appear that positive cognitive outcomes are generally associated with a "higher" amount or intensity of physical activity.

The literature that employed an exercise intervention (n = 12) provided some controversy in the findings. A slight majority of these studies (7 (58%)) demonstrated a positive effect on at least one measure of cognitive function. These studies employed moderate intensity aerobic physical activity interventions; however, it is difficult to quantify the actual volume of exercise used in each intervention. The positive studies generally showed small changes in usually just one or two cognitive measures. One study showed some beneficial cognitive functions scores with either moderate or higher intensity resistance training over 24 weeks [89]. Nevertheless in the negative studies the exercise intervention appeared to be very good and the finding of no significant change in outcome cognitive function measures extended across a number of variables. Thus, each of the negative studies appeared to be well-conducted [90-93]. Other studies in the cognitive function category were of a cross-sectional nature and again mixed results were found as to whether there was a relationship of physical activity level, or fitness level, with cognitive function measures. Therefore, there is mixed evidence to support or refute the benefits of habitual physical activity and/or exercise training on cognitive function in older individuals.

##### Interpretation

The quality of investigations was evaluated for the various studies (Table s11, see additional file 9). The Downs and Black [10] scale was varied according to the different types of study designs (as described earlier). Of the prospective/longitudinal designs, the studies were of generally good quality ranging from 8 to 11 (median 10.0 out of a possible 12). The randomised control trials were varied in quality ranging from 15 to 19 (out of possible score of 24) with a median score of 17.5. The non-randomised control intervention trials and the non-prospective cohort trials were generally of lower quality (median 15.0 out of 23) with a range of 14 to 17. It is important to note that there is insufficient data to conclude that a change in aerobic or musculoskeletal fitness is required to elicit positive changes in cognitive function [94,95]. Some evidence does however demonstrate positive changes in cognitive function with improvements in both aerobic [96] and musculoskeletal [89,97] fitness [95]. Moreover, and more convincing, there is consistent evidence that habitual physical activity reduces the subsequent risk for dementia and Alzheimer's disease in healthy older individuals [82,84,85,98].

##### Recommendation

It would appear that habitual (long-term) physical activity is associated with a decreased risk of dementia and Alzheimer's disease, with the evidence Level 2 and grade A. It would also appear exercise training can result in an improvement in cognitive function in healthy older adults. However, these conclusions must be tempered owing to the balance of negative and positive studies and considerable variability in the battery of tests used to measure cognitive function. Thus, it appears that there is a relationship between physical activity (probably of a relatively "high" level of physical activity) and better cognitive function outcomes; however, to date it cannot be concluded that an exercise training programme will necessarily maintain or improve cognitive function or in the longer-term alter the course of dementia. To make a recommendation for a "standard" aerobic or resistance training programme to affect cognitive function of older adults would at the moment be at Level 3, Grade B.

### Other factors to be considered related to physical activity recommendations

What other factors should be considered in recommendations of physical activity for older adults?

Stretching and flexibility exercises, primarily for shoulder and hip joints, may have some benefit in relation to function and may be recommended in rehabilitation where joint range of motion has been compromised by disuse or injury, but in general a recommendation of flexibility exercises is not supported by the scientific literature. Thus, although greater flexibility may facilitate some aspects of daily life, and stretching activities are incorporated into most physical activity programmes there are no known health benefits of greater flexibility and stretching exercises have not been demonstrated to reduce risk of activity-related injuries or reduce post-exercise muscle soreness. In fact pre-performance stretching exercises have been shown to reduce scores on some performance-related tasks (see [99]). Observation of many older adult activity classes and programmes gives evidence of an inordinate emphasis on flexibility and at the expense of aerobic and strength programming; thus in guidelines or recommendations it may be prudent to discourage spending an excessive amount of time in flexibility exercises and suggest stretching exercises be done at the conclusion of an activity session.

On the other hand a general whole body warm-up by gradually increasing the physical activity intensity up to that of the exercise session target level is advisable, and a gradual cool-down back toward baseline is a prudent safety factor to avoid "pooling of blood in the periphery" and potentially depriving the heart and brain of needed blood flow.

Comments on starting a programme of increased physical activity are also warranted. Often at the start of a programme it is not possible or feels too demanding to meet the recommended guidelines. Although scientists have not established a standard for how to gradually increase physical activity over time it is prudent (and a safe approach) to begin at a lower intensity and duration of a physical activity session (either in relation to aerobic or resistance exercises) and then incrementally progress to meet the duration goal and subsequently the intensity goal. Nevertheless research has shown that older adults do adapt physiologically to a given imposed exercise stress over a similar time frame to that observed in younger individuals [100]. Further exercise training programmes of community-dwelling older adults have been safe and effective with progression to 70% VO_2_max (vigorous exercise) within one or two weeks (and similar rapid progression in resistance training). If one starts too low and has a minimal "overload" the adaptation will be small, and if progression is too slow one loses the motivation of seeing and feeling, the "improvements" and gain in fitness and function; reward of improvement likely helps maintains adherence to the activity programme.

### Integration of Data from Different Study Designs and Different "Health" Outcomes

Studies of the relationship between long-term physical activity and morbidity and mortality outcomes were reviewed in the "adult" paper [1] and the previous review specific to older adults [2]. With regard to achieving a reduced risk of various morbidities, or of all-cause mortality, the recommended physical activity was of a moderate (brisk walking was a descriptor) with a total volume of 150 to 200 min/week to moderately-vigorous intensity (with >4.5 METS recommended, but scaled relatively lower in older adults) and volume of 90 min/week, and energy equivalent approaching 1000 kcal/week. Higher intensity was seen to engender additional benefit. There was little evidence to support the concept of accumulating or "counting-up" light, moderate short-duration activities like walking (here and there during the day), or short duration non-aerobic activities (taking stairs), or small muscle group activities (like raking leaves, or painting a wall). Evidence does support 10-min segments of "exercise training" being additive [101].

From the point of view of maintaining cardiorespiratory fitness above important functional thresholds (performance-related fitness) and postponing functional loss, the earlier review [2] determined that this could be achieved with a moderate exercise programme of 150 min/week or vigorous exercise of 90 min/week, but that light intensity activity or an accumulated volume of activity throughout the day was not effective in maintaining or increasing cardiorespiratory fitness of older adults.

The present systematic review focused specifically on functional independence outcomes. It is concluded that reduced risk of functional limitations and disability in older age can be achieved with moderate and moderately-vigorous physical activity, but not light activity, with a volume of 150 to 180 min/week or approximately 1000 kcal/week (4200 J/week) energy expenditure, and taking up physical activity or undertaking an exercise programme, usually of moderate intensity in the range of 150 min/week to moderately-vigorous aerobic exercise of 30 min/session and 3 times/week, is effective. There was a dose-response with greater volume in either the moderate or vigorous domain yielding further reduction in relative risk of functional limitations or disability.

Exercise intervention studies have also shown that resistance training of 2 times/week of the major muscle groups is recommended to preserve muscle mass and maintain strength and power for daily activities, and resistance training also receives some support from data related to morbidity and mortality outcomes. Physical activity appears also to have a role in prevention of dementia, and exercise interventions appear to improve some aspects of cognitive function, however statement of the details of the requisite dose of activity to achieve these benefits is to date premature. Thus, information is lacking to determine the dose of physical activity required for cognitive benefits or to modify the recommended activity levels based on the other outcome variables.

There is some concern that "significant" effects of physical activity were derived from studies in which the comparison was between the most sedentary groups and a moderate to moderately high active group and once a recommended physical activity level was established subsequent studies compared those below to those above the recommended level. Thus these cut points for active versus inactive have produced a "line in the sand" and a self-fulfilling prophecy that further studies would concur. Nevertheless, as reviewed, there are studies that have examined lighter volume or intensity of activities and not found a "meaningful" reduction in risk of the outcome variable and in studies examining a number of physical activity groups and noting a "significant" trend the concept of a dose-response has generally been that more volume in the moderate or moderately vigorous domains has had a greater effect. Thus, there is not the data to support a recommendation below the present commonly cited intensity levels, and it is reassuring to note that if the recommendation is "higher" than some minimum this greater dose is associated with a greater response - it is beneficial (there is a "response") to do more above a minimum or moderate level. It might also be noted that establishment of any minimum at present would be derived from comparison with a "least-active" group, whereas the recommendations are for physical activity above an individual's present baseline. Perhaps from a public health perspective it is prudent to note that there is a dose-response such that the least active or the average or even the more active segments of the population will all benefit from increasing their own physical activity levels.

How are moderate and vigorous interpreted in guiding physical activities for older adults? The following examples of what have been described as moderate and vigorous walking paces for older adults may help clarify the moderate and vigorous recommendations and the recommendations of total volume or energy expenditure per week:

• Moderate intensity walk of 3.0 mph = 3.3 METS = 11.6 ml/kg.min = (at 60 or 80 kg) ~700 - 900 ml/min VO_2 _(or 3.5 - 4.5 kcal/min) = ~46-58% VO_2_max (for older adult VO_2_max in the range of 20-25 ml/kg.min); and 180 min/wk = 630 - 810 kcal/wk = 594 MET.min.

• Vigorous intensity walk of 4.0 mph = 4.2 METS = 14.7 ml/kg.min = (at 60 or 80 kg) ~ 880 -1180 ml/minVO_2 _(or 4.4 - 5.9 kcal/min) = ~59-74% VO_2_max (for older adult VO_2_max in the range of 20-25 ml/kg.min); and 150 min/wk = 660 - 885 kcal/wk = 630 MET.min.

It should be noted that there is good evidence that the physiological and performance responses of older adults at a given "relative" intensity are similar to those of young adults. It has been demonstrated that performance time at a given intensity relative to VO_2_max, or to other relative intensity markers is similar in older adults to that observed in younger samples [102]. Thus, recommending exercise at a percentage of VO_2_max, or relative to other markers of exercise intensity in older adults is relatively similar to the recommendation for younger adults. For example, Overend et al. [103] documented similar achievement of quasi-steady state physiological responses with 24 minutes of exercise at critical power (highest sustainable exercise rate) in older compared to younger individuals, and in fact the older adults performed at approximately 90% of their VO_2_max compared to 85% of VO_2_max in young. Additionally, the "anaerobic" threshold (or estimated lactate threshold or gas exchange threshold) usually occurs at a higher percentage of VO_2_max for older versus younger adults (due to a greater age-related rate of loss in VO_2_max versus the threshold measure, e.g. Paterson et al., [104]) and thus relative to this marker of the intensity for sustained steady state exercise the recommended relative intensity could even be somewhat higher in older versus younger individuals. It should also be noted, however, that whereas physical activity recommendations in younger adults may halve the volume of vigorous activity versus moderate activity, in older adults there is a "narrow scale" between moderate and vigorous such that a vigorous intensity is much less than twice the moderate dose and one-half of the volume of vigorous does not yield a similar volume to that of moderate.

## Conclusions

### Dose of Physical Activity - Recommendations for Older Adults

The present systematic review emphasized the relationship between physical activity and functional independence and cognitive function outcomes in older adults. The data support the physical activity recommendations derived from analysis of the relationship between physical activity and morbidity and mortality outcomes. These data support the following recommendation:

Physical activity (above baseline "normal" daily activity levels) at an intensity of moderate to moderately vigorous aerobic (endurance) activity (3.3 to 4.2 METS; 3-4 mph walk; >50%VO_2_max), with a total weekly volume of 150 - 180 min/wk (3 hours at moderate pace or 2.5 hours of a more vigorous "brisk" walking, or other types of aerobic activities, with each physical activity session of greater than 10 minutes) and, a gain of 0.5 MET (~2 ml/kg.min) in cardiorespiratory fitness.

This physical activity would translate to a >30% decrease in the relative risk of morbidity and mortality, and loss of independence, and further benefit would accrue with greater physical activity and greater fitness gains (~60% reduction in risks).

Additionally, intervention studies of aerobic exercise training programmes for older adults support this intensity and amount of exercise as being effective in preventing functional limitations and potentially delaying mobility disability in older age. Exercise training interventions that supplemented the aerobic exercise by including twice per week "resistance" exercises of major muscle groups support a recommendation that there may be additional benefit in including resistance exercise (as an adjunct to the aerobic physical activity) to counter the age-related loss of muscle mass, and maintain the strength and power requirements needed in daily activities and to prevent falls.

These short-term intervention studies of exercise training have also suggested that the short-term response of improved fitness may translate into longer-term adherence to increased physical activity and thus it is appropriate for older adults to take up exercise to "Get Fit for Active Living" [105], and for physically active older adults to maintain their activity and fitness levels to postpone functional losses.

## Competing interests

DP and DW declare no competing interests.

## Authors' contributions

DP was the principal author of the text. DP also took responsibility for staff involved in cross-reference searching and adding these papers to the data extraction tables, and quality assessment of the studies (prospective, aerobic, resistance). DP and staff produced and edited the final manuscript.

DW took responsibility for the staff involved in the electronic search and preparation of the initial data extraction tables, cross-referencing for the cognitive section, and the quality assessment of the papers in the cognitive section. DW provided the initial authorship of the search methods and of the cognitive function section. DW assisted in reading and editing of the final manuscript.

## Supplementary Material

Additional file 1 **Supplemental tables 1-3**. Table s1: Results of literature MEDLINE search regarding the relationship between physical activity/exercise and functional limitations in the elderly. Table s2: Results of literature MEDLINE search regarding the relationship between physical activity/exercise and cognitive function in the elderly. Table s3: levels and grade of evidence scaling criteria applied to the recommendations. Click here for file

Additional file 2 **Supplemental table 4**. Table s4: Prospective (longitudinal) cohort studies examining the relationship between physical activity and functional limitations in older adults [106-110]. Click here for file

Additional file 3 **Supplemental table 5**. Table s5: Prospective studies assessed with the modified Downs and Black Quality Assessment Tool. Click here for file

Additional file 4 **Supplemental table 6**. Table s6: Aerobic or combined exercise training studies examining the relationship between physical activity and functional limitations in older adults [111-121]. Click here for file

Additional file 5 **Supplemental table 7**. Table s7: Aerobic or combined exercise studies assessed with the modified Downs and Black Quality Assessment Tool. Click here for file

Additional file 6 **Supplemental table 8**. Table s8: Resistance/strength or functional training studies examining the relationship between physical activity and functional limitations in older adults [122-130]. Click here for file

Additional file 7 **Supplemental table 9**. Table s9: Strength studies assessed with the modified Downs and Black Quality Assessment Tool. Click here for file

Additional file 8 **Supplemental table 10**. Table s10: Studies examining the relationship between physical activity and cognitive function in older adults [131-148]. Click here for file

Additional file 9 **Supplemental table 11**. Table s11: Cognitive studies assessed with the modified Downs and Black Quality Assessment Tool. Click here for file
