# Supplementary material for: Physical activity and functional limitations in older adults: a systematic review related to Canada's Physical Activity Guidelines
Source: Int J Behav Nutr Phys Act. 2010 May 11;7:38. doi: 10.1186/1479-5868-7-38 (PMC2882898; doi:10.1186/1479-5868-7-38)
Supplement: Additional file 1 — Supplemental tables 1-3. Table s1: Results of literature MEDLINE search regarding the relationship between physical activity/exercise and functional limitations in the elderly. Table s2: Results of literature MEDLINE search regarding the relationship between physical activity/exercise and cognitive function in the elderly. Table s3: levels and grade of evidence scaling criteria applied to the recommendations. [file 1479-5868-7-38-S1.DOC]

**Table s1. Results of literature MEDLINE search regarding the relationship between physical activity/exercise and functional limitations in the elderly.**

**Functional Limitations**

| **#** | **Searches (March 1, 2008)** | **Results** |
| --- | --- | --- |
| 1 | exp Physical Fitness/ | 15558 |
| 2 | Motor Activity/ | 50433 |
| 3 | exp Physical Endurance/ | 15584 |
| 4 | exp Exercise/ | 58543 |
| 5 | exp Exertion/ | 90055 |
| 6 | exp Sports/ | 73177 |
| 7 | exp exercise therapy/ | 17641 |
| 8 | exp exercise tolerance/ | 4265 |
| 9 | exp health behaviour/ | 60212 |
| 10 | leisure time physical activity.mp | 1025 |
| 11 | occupational physical activity.mp | 193 |
| 12 | exp Muscle Strength/ | 6067 |
| 13 | musc$ power.mp | 987 |
| 14 | exp Back/ | 13106 |
| 15 | muscle stretching exercises/ | 114 |
| 16 | exp Range of motion, articular/ | 18745 |
| 17 | yoga/ | 816 |
| 18 | muscle contraction/ | 76831 |
| 19 | isometric contraction/ | 10106 |
| 20 | isotonic contraction/ | 529 |
| 21 | musculoskeletal equilibrium/ | 8400 |
| 22 | 1 or 2 or 3 or 4 or 5 or 6 or 7 or 8 or 9 or 10 or 11 or 12 or 13 or 14 or 15 or 16 or 17 or 18 or 19 or 20 or 21 | 390266 |
| 23 | dose response.mp | 323761 |
| 24 | intensity.mp | 145959 |
| 25 | volume.mp | 302589 |
| 26 | exp Energy Metabolism/ | 208167 |
| 27 | exp oxygen consumption/ | 84093 |
| 28 | exp time factors/ | 773501 |
| 29 | 23 or 24 or 25 or 26 or 27 or 28 | 1671790 |
| 30 | exp “Activities of Daily Living”/ | 34691 |
| 31 | Health Status | 36954 |
| 32 | exp Geriatric Assessment/ | 11393 |
| 33 | Disabled Persons/ | 24886 |
| 34 | Hypokinesia/ | 657 |
| 35 | exp disability evaluation/ | 25364 |
| 36 | Mobility limitation/ | 410 |
| 37 | “Quality of Life”/ | 66330 |
| 38 | 30 or 31 or 32 or 33 or 34 or 35 or 36 or 37 | 172112 |
| 39 | 22 and 29 and 38 | 3071 |
| 40 | Limit 39 to (English language and humans and “all aged (65 and over)” | 1209 |

**Table s2. Results of literature MEDLINE search regarding the relationship between physical activity/exercise and cognitive function in the elderly.**

**Cognitive Function**

| **#** | **Searches (March 1, 2008)** | **Results** |
| --- | --- | --- |
| 1 | exp Physical Fitness/ | 15558 |
| 2 | Motor Activity/ | 50433 |
| 3 | exp Physical Endurance/ | 15584 |
| 4 | exp Exercise/ | 58543 |
| 5 | exp Exertion/ | 90055 |
| 6 | exp Sports/ | 73177 |
| 7 | exp exercise therapy/ | 17641 |
| 8 | exp exercise tolerance/ | 4265 |
| 9 | exp health behaviour/ | 60212 |
| 10 | leisure time physical activity.mp | 1025 |
| 11 | occupational physical activity.mp | 193 |
| 12 | exp Muscle Strength/ | 6067 |
| 13 | musc$ power.mp | 987 |
| 14 | exp Back/ | 13106 |
| 15 | muscle stretching exercises/ | 114 |
| 16 | exp Range of motion, articular/ | 18745 |
| 17 | yoga/ | 816 |
| 18 | muscle contraction/ | 76831 |
| 19 | isometric contraction/ | 10106 |
| 20 | isotonic contraction/ | 529 |
| 21 | musculoskeletal equilibrium/ | 8400 |
| 22 | 1 or 2 or 3 or 4 or 5 or 6 or 7 or 8 or 9 or 10 or 11 or 12 or 13 or 14 or 15 or 16 or 17 or 18 or 19 or 20 or 21 | 390266 |
| 23 | dose response.mp | 323761 |
| 24 | intensity.mp | 145959 |
| 25 | volume.mp | 302589 |
| 26 | exp Energy Metabolism/ | 208167 |
| 27 | exp oxygen consumption/ | 84093 |
| 28 | exp time factors/ | 773501 |
| 29 | 23 or 24 or 25 or 26 or 27 or 28 | 1671790 |
| 30 | exp Cognition/ or exp Cognition Disorders/ | 101611 |
| 31 | exp Dementia/ or exp Alzheimer Disease/ | 80660 |
| 32 | exp Dementia, vascular/ | 3999 |
| 33 | 30 or 31 or 32 | 167580 |
| 34 | 22 and 29 and 33 | 764 |
| 35 | Limit 34 to (English language and humans and “all aged (65 and over)” | 180 |

**Table s3. The levels and grade of evidence scaling criteria applied to the recommendations.**

| **Level of Evidence** | **Criteria** |
| --- | --- |
| Level 1 | Randomized control trials without important limitations |
| Level 2 | - Randomized control trials with important limitations - Observational studies (non-randomized clinical trials or cohort studies) with overwhelming evidence |
| Level 3 | Other observational studies (prospective cohort studies, case-control studies, case series) |
| Level 4 | Inadequate or no data in population of interest  Anecdotal evidence or clinical experience |

| **Grade of Evidence** | **Criteria** |
| --- | --- |
| Grade A | Strong recommendation (action can apply to most individuals in most circumstances)   - Benefits clearly outweigh risks (or vice-versa) - Evidence is at Level 1, 2, or 3 |
| Grade B | Weak recommendation (action may differ depending on individual’s characteristics or other circumstances)   - Unclear if benefits outweigh risks - Evidence is at Level 1, 2, or 3 |
| Grade C | Consensus recommendation (alternative actions may be equally reasonable)   - Unclear if benefits outweigh risks - Evidence is at Level 3 or 4 |
