# Supplementary material for: Physical activity and functional limitations in older adults: a systematic review related to Canada's Physical Activity Guidelines
Source: Int J Behav Nutr Phys Act. 2010 May 11;7:38. doi: 10.1186/1479-5868-7-38 (PMC2882898; doi:10.1186/1479-5868-7-38)
Supplement: Additional file 2 — Supplemental table 4. Table s4: Prospective (longitudinal) cohort studies examining the relationship between physical activity and functional limitations in older adults [106-110]. [file 1479-5868-7-38-S2.DOC]

| **Publication**  **Country** | **Objective** | **Population** | **Methods** | **Outcomes** | **Comments and Conclusions** |
| --- | --- | --- | --- | --- | --- |
| Al Snih et al., 2004 [22]  USA | To examine the association between hand grip strength and 7 y incidence of ADL disability | - N=2493 - Sex: men and women - Age: >65 y; mean 73 y - Mexican American in EPESE data base | Follow-up at 2, 5, and 7 y  Measurements:  ADL disability  Physical activity: Hand grip strength  Cox proportional hazards | Linear relationship of hand grip strength at baseline and risk of incident ADL disability – a stepwise decrease in risk with greater hand grip strength  Risk of new ADL limitation in lowest quartile compared to highest of hand grip strength:  Men: HR 1.90, CI 1.14-3.14  Women HR 2.28, CI 1.59-3.27 | Hand grip strength is an independent predictor of ADL disability |
| Bäckmand et al., 2006[19]  Finland | To determine the role of physical activity in the physical and psychological functioning of daily living in a cohort of former elite male athletes | - N=1164 - Sex: men Age: 59-70 y - 664 athletes (64.6 y) - 500 controls   (62.0 y)   - Former Finnish national athletes | Follow-up 10 y  Measurements: Physical functioning of daily living (heavy house cleaning, dressing and undressing, writing, shopping or banking, travelling by public transport)  Physical activity: Questionnaire on recreational physical activity; 5 MET index quintiles  Logistic Regression | Low levels of physical activity in 1985 (OR 4.91, CI 2.02-11.9) increased the risk of poor physical function in daily living in 1995.  Increased levels of physical activity (OR 0.89, CI 0.83-0.95) between 1985-1995 protected against poor physical functioning  The previously athletic group did not reveal any protective effect on physical function | An increase in physical activity supports physical daily functionality |
| Berk et al., 2006[14]  USA | To assess the effect of changes in exercise (and other risk factors) on disability or health-related quality of life in seniors who were originally inactive and increased exercise or who exercised and decreased activity | - n=549 - Sex: 73% men, 27% women - Age: Baseline mean 58 y and follow-up 74 y - From comparative study of vigorous exercisers (runners) and population controls - “specialized” sample | Follow-up 16 y  Measurements:  Assessment of Disability: HAQ-D1; Health Assessment Questionnaire (HAQ) 21 questions scored as no difficulty, some, much, and inability to perform  Vigorous exercise of greater than 60 min/wk (High) or less than (Low) initial and in follow-up periods  Sedentary (low–low)  Exercise increasers (low–high)  Exercise decreasers (high–low)  Exercisers (high-high)  ANOVA (covariance) on adjusted change in HAQ | Increase in HAQ scores:  Sedentary 0.37 units; (Indicates difficulty in 3 domains of daily function, or inability in one)  Decreasers 0.28 units;  Increasers 0.17 units;  (Indicates “good” end of study scores)  Exercisers 0.11 units  Final disability score associated with current more than initial exercise group; Change in HAQ for Increasers versus Sedentary significantly more favourable with multivariate statistical adjustment | Beneficial effect of exercise (at high levels), even when begun later in life, on postponement of disability  Re comments on physical activity: starting, increasing, or maintaining vigorous exercise particularly walking, cycling, or aerobics |
| Boyle et al., 2007 [33]  USA | To examine association between physical activity and risk of incident disability (ADL and IADL) in elderly, community-dwelling | - N=1020 - Sex: Men and Women - Age: mean 80.5 y - Sample free of dementia; analysis for those with no disability at baseline | Average follow-up of 2.6 y (annual evaluations)  Measurements: ADL and IADL with scale of no help, help, unable to do  Assessed gait speed to control for baseline gait  Physical activity: walk for exercise, gardening, calisthenics or exercise, cycle, swim in previous two weeks; analyzed as hours per week  Cox proportional hazards models adjusted for many factors | Of the 26% who became ADL disabled in follow-up the relative risk of disability was 7% less for every additional hour of physical activity (HR 0.93, CI 0.88-0.98); thus, 16% less disability for 2.33 hr/wk (50^th^%ile) and 41% less for 7 hr/wk (90^th^%ile)  Of the 55% who became IADL impaired in follow-up the relative risk of disability was 7% less for every additional hour of physical activity (HR 0.93, CI 0.89-0.98); thus 21% less for 2.8 hr/wk activity and 47% less for 7.5 hr/wk activity  Also risk of mortality was 24% less with 2.3 hr/wk and 57% less with 7 hr/wk of physical activity | In older persons without dementia, physical activity is associated with maintenance of functional status, and reduced risk of impairment in ADL and IADL |
| * Buchman et al., 2007 [49] |  |  | Outcome was mobility decline from 8 foot walk and 360 degree turn  Leg strength also measured | Each additional hour of physical activity was associated with an ~3% decrease in rate of mobility decline  A higher level of leg strength was associated with a slower rate of mobility decline | Both physical activity and leg strength were independent predictors of mobility decline in older persons |
| Brach et al., 2003 [20]  USA | To determine the relationship of physical activity to functional status over a long-term | - N=229 - Sex: women - Age: mean 74 y at follow-up (i.e. initially ~60 y) - Initial cohort involved in a randomized walking intervention, and followed-up 14 y - A very high functioning group (75^th^ to 90^th^ %ile of function) | Follow-up 14 y, with physical activity at initial, 10 and 14 y  Measurements:  Functional status questionnaire (FSQ) of ADL and IADL  Physical Performance Test (PPT) of 7 items of daily activities and gait speed over 4 m  Physical activity questionnaire of walking for exercise and sports; and total activity averaged over year; and used objective measures of monitors  Grouped into met or did not meet Surgeon General recommendation; and by above and below median for objective activity counts; and from the 3 test times – always active, never active or inconsistently active  Statistics of between group comparisons; multivariate linear regression models of past physical activity and gait speed | Initial and 10 year physical activity (by questionnaire or objective) was a significant independent predictor of gait speed at the 14 y follow-up  The greater consistency of physical activity the better the functional status (PPT) and ADL and gait speed; tests for trends across physical activity levels were significant | Physical activity was related to functional status 14 years later measured by various methods of self-report ADL function, physical performance or gait speed |
| * Brach et al., 2004[45]  USA | To examine the cross-sectional and longitudinal associations among obesity, physical activity, and physical function over a 17-year time period in community-dwelling older women | - N= 171 - Sex: Women - Age: mean baseline 60 y to follow-up mean 74 y - Initially were in a walking intervention trial | Follow-up 14 y  Measurements:  FSQ re ADL, and gait speed on 4 m walk at 14 y  Initial and 3 follow-up of BMI, and Physical Activity of blocks walked and sport leisure – calculated total activity per week over past year (active 1000 kcal/wk; 30 min moderate on most days);  ANOVA between groups  Hierarchical regression | Obesity and physical activity measures were both related to physical function status. Always active and never overweight group had least difficulty with ADL and walked faster and never active and always overweight had greatest  ADL difficulty and slowest gait speed  Both weight and physical activity were independent predictors of FSQ and gait speed but when both entered into regression physical activity and not obesity was an independent predictor of physical function both FSQ and gait speed | Physical activity appears to be as important or more important than body weight in prediction future physical function |
| Bruce et al., 2008 [15]  USA | To examine the relationship of regular exercise and body weight to disability among a cohort of healthy older adults | - N=805 - Sex: Men and women - Age: 50-72 y; mean 65 y - Assessed yearly - Recruited from national runners association and control sample | Follow-up 13 y  HAQ-DI – disability index of 8 categories scaled from 0 to 3  Physical activity: minutes per week of vigorous exercise grouped as Active > 60 min (mean 250-300 min/wk) and inactive < 60 min (mean ~25 min/wk)  Self-reported BMI  4 groups: normal-weight active and inactive and overweight active and inactive  Multivariate analysis and generalized estimating equations | Physically inactive had significantly more disability than active regardless of weight group (i.e., in both weight groups) | Regular physical activity postpones disability  Preventive approach to reduce functional impairment associated with overweight through physical activity |
| Christensen et al., 2006 [28]  Denmark | To analyze the impact of physical inactivity from middle age to early old age on functional ability at age 75 | - N=387 - Sex: men and women - Age: Baseline age 50 y; follow-up at 75 y | Follow-up at 10, 20 and 25 y  Measurement:  Functional ability at age 75 from “tiredness” with six mobility activities grouped as good function vs. poorer function  Physical activity as cumulated activity from age 50 to 60 to 70 and at the 3 separate time points from self-report grouped to mainly sedentary, slightly active, moderate activity and vigorous activity; and in this analysis grouped into mainly sedentary or mainly active  Logistic regression analysis | Strong association between physical inactivity at age 70 and disability at age 75, but, no effect of cumulated physical inactivity from age 50-60-70 on disability at age 75 when adjusting for functional ability at age 70  Thus, physical inactivity at age 70 related to disability at age 75 OR 5.07, CI 1.08-23.89; inverse 0.17, CI 0.04-0.93 | Physical inactivity is a risk factor for disability among old people  Old should be encouraged to take up and maintain physical training throughout the aging process |
| Clark, 1996 [46]  USA | To examine the association between regular physical activity and increase of lower body disability in older, community-dwelling Blacks and Whites | - N=3841; 413 Blacks and 3428 Whites - Sex: Men and women - Age: >70 y - Longitudinal Study on Aging | Follow-up at 2, 4 and 6 y  Measurements: Lower body disability – walking ¼ mile, 10 stairs; standing 2 hr, crouching/kneeling, carrying 25 lbs  Physical activity: Walking 1 mile or more – 2X/wk, 2-3/wk, 4-7/wk  And also regular exercise routine  Discrete time hazard models | Each of the walking categories vs. never walking 1 mile was significant in predicting increased lower body disability in Whites but only walking 4-7 times/wk reached significance in Blacks; the effect of regular exercise was not significant for either race  For onset of specific lower body disabilities the risk in Blacks ranged (depending on the disability measure) from 0.17 to 0.54 and in Whites from 0.49 to 0.93 (with some non-significant)  For walking ¼ mile the odds ratios were expressed vs. never walking for 1 d/wk, 2-3 d/wk or 4-7 d/wk  For Blacks 0.31, 0.29, 0.17; For Whites 0.56, 0.52, 0.49; Thus showing little gradient with walking frequency | Walking 4 to 7 days/wk reduced the risk of disability onset by 50-80% on all five disability items within the Black sample and by 50% on two items within the White sample |
| Dunlop et al., 2005 [36]  USA | To evaluate the prevalence of functional limitations in older adults with arthritis and the frequency of functional decline over 2 years | - n=5,715 - Sex: Men and women - Age: >65 y; mean ~75 y - Characteristics: Older adults with arthritis from a national probability sample - ~20% had limitations at baseline | Follow-up 2 y  Measurements:  Questionnaires  Function defined based on ability to perform basic ADL tasks and IADL; progression – none, mild, moderate, severe  Vigorous activity: over past 12 mo. participated 3+X/wk in vigorous sports, heavy housework or job of physical labour  Multiple logistic regression adjusted odd ratios (OR) | Function declined in 13.6% of group over 2-y  Most prevalent risk factor was lack of regular physical activity (64%)  Risk of functional decline, progression to more severe level of functional limitation OR 1.9, CI 1.5-2.4. Inverse OR 0.53, CI 0.42-0.67  If all had engaged in regular vigorous physical activity could reduce the functional decline by 32% | Lack of regular vigorous physical activity is a potential modifiable risk factor that could reduce functional decline in ADL and IADL in older adults with arthritis |
| Ebrahim, et al., 2000 [37]  UK | To examine the relationship between lifestyle and other risk factors in men aged 40-59 y at baseline and locomotor disability 12-14 years later, and disability | - N=5717 - Sex: men - Age: 40-59 y, mean ~50 y with mean at follow-up ~63 y (52-73 y) | Follow-up 12-14 y  Measurements: Self-reported locomotor disability re getting outdoors, walking 400 m, climbing stairs, and others  Physical activity of regular walking or cycling, recreational activity and sporting activity and frequency to give activity score; six groups from non- to vigorous and 2 groups with physically active being moderately active or more  Logistic regression | Compared to inactive the adjusted odds of locomotor disability for occasional active and for light OR 0.7, CI 0.5-0.8; for moderate and moderately-vigorous OR 0.4, CI 0.3-0.5 | Physical inactivity was a strong predictor of locomotor disability later in life independent of the presence of diagnosed disease |
| * Wanna-methee et al., 2005 [21]  UK | To examine predictors of the onset (and recovery) from mobility limitation and the association between lifestyle changes in later life and mobility status | N=5075  Sex: Men   - Age: as above with follow-up 4 more years (age 56-77 y) | Follow-up ~ 16 y  Measurements: Self-reported disability in getting outdoors, walking 400 m and climbing stairs  Physical activity assessed as above and also categorized by change between 12 and 16 y follow-up; stable inactive, became inactive, became active and remained active  Logistic regression | Physical activity of moderately vigorous or vigorous reduced the odds associated with onset of mobility limitation OR 0.66, CI 0.50-0.87, but was marginally non-significant after adjustment for chronic disease  Taking up physical activity in later life was associated with a reduced onset of mobility limitation, with mobility limitation in those who became active having OR 0.35, CI 0.22-0.56  In those with mobility limitation in the first follow- up, light or moderate levels of physical activity were associated with increased odds of recovery | Taking up physical activity even in later life was associated with reduced onset of mobility limitation in older age, and physical activity may promote recovery from mobility limitation |
| Ferrucci et al., 1999 [47]  USA | To determine the effects of smoking and physical activity on active life expectancy and disabled life expectancy | - N=8604 - Sex: Men and women - Age: > 65 y; mean ~ 74 y - EPESE study data   No disability at baseline | Follow-up 6 y, annually  Measurements: Self-report ability for ADL (need help or inability to perform = disability)  Physical Activity in walking gardening and vigorous exercise with composite score categorized as low (bottom quartile) moderate (two middle quartiles) and high (upper quartile  Also smokers classed as “ever” or “never”  Markov models fit to the data | In both men and women each increment of physical activity was associated with a lower risk of dying, a substantially lower risk of new disability, and a higher probability of functional recovery  In never smokers, compared with low active, in moderate and high active increase in active life expectancy was 3.3 and 5.1 years in men and 3.5 and 5.7 years in women; there was little difference across physical activity groups in disabled life expectancy  In ever smokers, compared with low active, in moderate and high active increase in active life expectancy was 1.0 and 3.4 years in men and 1.5 and 4.2 years in women; moderate and high showed a greater active life expectancy  There was an effect of compression of morbidity, i.e., physical activity reduces the duration of disabled life by approximately 1 to 2 y | Physical activity in the older population prolongs active life, decreases the percentage of remaining life that is spent in disability and for a given age at death is associated with a shorter period of disability |
| Giampaoli et al., 1999 [23]  Finland, Italy, Netherlands | To verify if hand grip performance in older men is a predictor of disability | - N=141 - Sex: Men - Age: 71-91 y; mean ~ 76 y - No disability in ADL, IADL, or mobility at baseline | Follow-up 4 y  Measurement: Disability in ADL, IADL or mobility  Physical activity: Hand grip strength  Logistic regression analysis | Reduced hand grip strength predicted disability in men 77 y and older | Poor hand grip strength is a predictor of disability in older people |
| Haight et al., 2005 [38]  USA | To examine the effects of leisure time physical activity (LTPA) and body composition on transitions in physical functioning in the elderly; population based cohort in 1993-1994 | - n=1,655 (males 708, females 947) - Sex: Men and women - Age: >55 y (median 70 y); with 6-y follow-up - Characteristics: Healthy and community dwelling elderly | Four surveys over 6 years  Self-reported physical functional limitation based on 10 questions  Measurements of LTPA: Avg. weekly participation over past 12mo. in 22 activities;  Continuous variable (Mets.min/day) Categories: 1. None 2. Insufficient 3. >22.3 - <35 (minimum recommended for brisk walking) 4. >35 – exercised at high aerobic levels  Body composition – bioelectrical impedance (lean-mass:fat-mass ratio)  MSMs) marginal structural models, stochastic | New physical limitations occurred in 16% of women and 9% of men  Physical activity decreased new physical limitation (namely moving/lifting, but not for walking) by:  Men 36.8% (CI 0.0-92.2%); functional limitation OR 0.63, CI 0.00-0.92  Women 52.7% (CI 13.5-91.9%); and delayed onset of functional limitation; functional limitation OR 0.47, CI 0.14-0.92 | High aerobic levels (> 35 MET.min/day exceeding recommendations) of physical activity appeared to reduce the risk of future functional limitation; “conditional” on the level of functioning established early in the disablement process by lean-mass:fat-mass ratio  Also, a lower risk of functional decline in those who increased pa over time |
| * Tager et al., 2004 [106]  USA |  |  |  | Functional limitation with a 0.5 unit increment in relative muscle mass was OR 0.56, CI 0.46-0.67 in females and OR 0.77, CI 0.65-0.92 in male | Leisure time physical activity exerts its beneficial effect through reductions in fat mass relative to lean body mass |
| Haveman-Nies et al., 2003 [29]  (also reviewed in Haveman-Nies et al., 2003 [107], Age and Ageing)  Europe | To investigate the effect of healthy lifestyle behaviours (including physical activity) on self-rated health and self-care ability i.e., functional independence in older adults | N=480  Sex: men and women  Age: 70-75 y | Follow-up 10 y  Measurements: ADL and degree of difficulty with functional independence defined as no difficulty or difficulty in performing only 1 self-care activity  Physical activity by questionnaire two groups: low tertile inactive group vs. intermediate and high activity tertiles  Logistic regression | Inactivity increased the risk of dependence in men OR 1.9, 90% CI 0.9-3.9 and women OR 2.6, CI 1.4-4.9 (Inverse OR men 0.53, 90% CI 0.26-1.11; women OR 0.38, CI 0.20-0.71) | Being physically active delayed onset of functional dependence in older participants aged 70-75 y |
| Hirvansalo et al., 2000 [35]  Finland | To study physical activity and the interaction of physical activity and mobility impairment as predictors of dependence (and mortality) | N=1109  Sex: Men and women  Age: 65-84 y  Evergreen Project subjects  independently living at baseline | Follow-up 8 y  Grouped into: Mobile-active, mobile-sedentary, Impaired- active, Impaired- sedentary | Odds for dependency (with adjustment for confounders including physical exercise earlier in life):  Impaired-sedentary men OR 5.21, CI 1.44-18.70 women OR 2.92, CI 1.52-5.60 compared to Mobile-active groups;  However risk of dependence did not differ significantly between Mobile-active and mobile sedentary; men OR 1.10, CI 0.27-4.55 or inverse OR 0.91 (0.22-3.70) and women OR 0.85, CI 0.45-1.59 or inverse OR 1.17, CI 0.63-2.22;  And also did not differ for impaired active groups | Among people with impaired mobility physical activity was associated with lower risks whereas the risk did not differ according to activity level among those with intact mobility  Mobility impaired people may be able to prevent further disability by physical exercise |
| Huang et al., 1998 [25]  USA | To examine the association between physical fitness, physical activity, and the prevalence of functional limitation | N=4670; N=577 age 60+ y  Sex: Men and Women  Age:40 y at baseline (but analysis also done on the 60+ y group of n=577) | Follow-up mean, 5.5 y  Measurements:  Initial medical assessment and functional status by mail survey follow-up; functional status was for daily and household activities of moderate intensity and of strenuous intensity  Fitness: treadmill test; low (bottom20%; moderate next 40%; high, top 40%)  Physical activity – in past 3 months particularly walking/running, work-outs; sedentary – no regular activity; moderate; some participation, less than 10 miles per week; active, equal to more than 10 mi per week  Logistic regression models | An inverse gradient (significant linear trend) of prevalence of functional limitation with fitness and activity categories was found for men and women in every age group  for men 60+ y for those of low moderate and high fitness prevalence of functional limitation was 24%, 9% 7%; and for women 46%, 26% 18%;  and for activity of sedentary, moderate and active in men 14%, 15% 9%; and women 31%,29%,22%  Across entire age range, logistic models: a lower prevalence of functional limitation than low fit (controlling for other factors) for men high (OR 0.3, CI 0.2-0.6) and moderately (OR 0.4, CI0.2-0.6) fit, and for women high (OR 0.3, CI 0.2-0.5) and moderately fit (OR 0.5, CI 0.3-0.7); similar association for physical activity men (OR 0.7 and 0.5) and women (0.7 and 0.7) and physical limitation was significant in men but not women | The results support a protective effect of physical fitness and physical activity on functional limitation among older adults |
| Koster et al., 2008 [40]  USA | To examine the joint associations of physical activity and adiposity measures with incident mobility limitation in Black and White older adults | N=2982  Sex: Men and women  Age: 70-79 y  Health, Aging and Body Composition (Health ABC) study  Initially well-functioning | Follow-up with semi-annual assessments over 6.5 y  Measurements: Mobility limitation of reported difficulty walking ¼ mile or 10 stairs  Physical activity from questionnaire of past 7 days all activities and duration and intensity coded to derive summed overall activity score; low (bottom quartile), medium (middle two quartiles), high (highest quartile)  Adiposity from BMI, total % body fat, waist circumference  Cox proportional hazards | Low physical activity was associated with greater risk of mobility limitation in all groups (men and women, Black and White) with consistent significant differences comparing the high to low activity with HR ~1.5 or slightly higher; inverse HR 0.67.  For the combined effects of any of the adiposity measures and physical activity in each adiposity group incidence rates of mobility limitation were higher in the low physical activity groups and incident mobility limitation was predicted more strongly with high adiposity and low physical activity than when analyzed separately; in general the HR was >2 (inverse 0.5) for low physical activity across different adiposity groups | High adiposity and low physical activity both predicted the onset of mobility limitation in older persons |
| * Koster et al., 2007 [108] | To examine the association between incident mobility limitation and lifestyle factors including physical activity in obese and non-obese older adults | As above |  | In non-obese risk for incident mobility limitation in low and medium physical activity groups (compared with high) HR 1.78, CI 1.45-2.18 and HR 1.29 CI 1.07-1.54 (Inverse: HR 0.56, CI 0.46-0.69 and HR 0.78, CI 0.65-0.93)  In obese risk for incident mobility limitation only significant in low physical activity groups (compared with high) HR 1.44, CI 1.08-1.92 (inverse: HR 0.69, CI 0.52-0.93) | Underscores the importance of a healthy lifestyle, particularly physical activity for maintaining function, although a healthy lifestyle did not overcome the effect of obesity in older adults |
| Lang et al., 2007 [39]  USA and England | To assess association between physical activity and subsequent physical functioning in middle-aged adults across a range of BMI categories | N=8,702 (US) and 1507 (England)  Sex: Men and women  Age: 50 to 69 y; mean age 60 y and 58 y with 6-y follow-up  Characteristics: free of disability at baseline | 6 yr follow-up  Measurements:  Self-reported BMI and physical activity (past 12 months) US: participated in vigorous physical activity or exercise 3 times/wk or more England: heavy housework, gardening, manual labour, brisk walking, sports – summary of days per week for 30 min or longer – category 3 times/wk or more.  Physical Performance Battery, modified: balance, chair stands (timed), grip strength; and self-report mobility impairment (difficulty walking several blocks, or flights of stairs)  Logistic regression modeling | In all weight categories higher levels of physical activity were associated with lower risks of mobility impairment  At BMI 20-25 relative risk of incident mobility difficulty for “vigorous group” vs. less active OR 0.56, CI 0.40-0.78; for BMI > 30 OR 0.59, CI 0.45-0.76 | Excess body weight is a risk factor for impaired physical function in middle-aged and older, however...  Physical activity (of >30 min of vigorous 3 times/wk) in middle-aged is protective of impaired physical functioning 6-y later in subjects of all weight categories |
| Leveille et al., 1999 [26]  USA | To estimate the prevalence of having no disability in the year prior to death in very old age and to examine the factors (including physical activity) associated with this outcome | - N = 1097 (from EPESE) - Sex: men and women - Age: >65 y, mean ~ 80 y; who died after age 85 y in women and age 80 y in men with no prior disability within 15 months | Average follow-up time 6 y  Measurements: Disability by questionnaire of needing help or unable to perform any of six ADL tasks  Self-rated health and chronic conditions by self-report  Physical activity: Low, moderate, or high based on summed frequency of walking, gardening and vigorous exercise  Multivariate logistic regression modeling | Physical activity was related to probability of survival to age 80 or 85 y  Not being disabled before death: men, most active were 58% vs. least active 43%; women 41% and 22%  Combined probability of survival to old age and dying without disability from age 65 y:  Men, most active 37% vs. least active 15%; women, most active 29%, least active 10%  Higher levels of physical activity nearly doubled the likelihood of being free from ADL disability prior to death OR 1.86, CI 1.24-2.79 (intermediate level of physical activity not significant compared to low activity) Inverse OR 0.53, CI 0.36-0.80 | Physical activity was a key factor predicting non-disability before death with a twofold likelihood of dying without disability in the most physically active versus sedentary.  Evidence that disability prior to death may be prevented by moderate (the high group mainly reported walking and gardening, not vigorous exercise) physical activity |
| Østbye et al., 2002 [27]  USA | To analyze the effect of modifiable risk factors (including exercise) on ill-health, including disability and impaired mobility | - N= 7845 - Sex: men and women - Age: aged 50-60 y (Health and Retirement Study HRS) - [And 5037 aged 60-70 y (Asset and Health Dynamics among the Oldest Old survey AHEAD) – not in physical activity analysis] | Follow-up at intervals for 5 to 6 y  Measurements: Disability in work or ADL, and in AHEAD also used IADL; Impaired mobility – walking up one flight of stairs, walk several blocks  Physical activity – number of times per week participate in light physical activity and in vigorous physical exercise; overall classed as sedentary, light, moderate, vigorous  No “exercise” measure in AHEAD  Multivariate logistic regression and models | Exercise at baseline showed a protective benefit against subsequent dependence in ADL in a straight-line fashion; compared to sedentary, light exercise group half as likely to be dependent in one ADL, OR 0.53, CI 0.43-0.66 and for moderate exercise OR 0.44, CI 0.36-0.52 and vigorous exercise OR 0.22, CI 0.22-0.36  And the physical activity level showed a protective benefit against Disability: vs. sedentary, light exercise OR 0.51, CI 0.40-0.64, moderate OR 0.46, CI 0.37-0.55 and heavy OR 0.25, CI 0.19-0.33  And linear protective benefit in stair ability and ability to walk blocks | There was a strong dose-response relationship between amount of exercise and all the outcomes considered and heavy exercise had a stronger effect than moderate exercise |
| Paterson et al., 2004 [5]  Canada | To describe those factors that were determinants of becoming dependent at the 8 y follow-up | - n=373 (at follow-up n=279) - Sex: Men and women - Age: 55-86 y, mean ~ 70 y - Characteristics: Ambulatory and independent | Baseline and 8 y follow-up  Measurements:  8 y follow-up 20 min telephone interview  Dependence – if they resided in a chronic facility, nursing home, senior home with care facility, required a wheelchair, or walker, or home-care >1 time/wk  Body size, presence of chronic disease, VO_2max_, strength, flexibility, walking pace, leisure time physical activity (Minnesota Leisure-Time Physical-Activity (LTPA) questionnaire)  Modeling and multivariate analysis, multiple logistic regression | At follow-up, of the 279 participants 43 were identified as being dependent  Baseline age, presence of disease, and VO_2max_ increased odds of becoming dependent  Lower VO_2max_ increased the odds of dependence, OR 0.86 CI 0.74-0.99 for a difference of 1 ml/kg.min in VO_2max_ i.e., 14% lower occurrence of dependence per unit higher VO_2max_ | Lower cardio-respiratory fitness was a significant determinant of becoming dependent in an 8-y follow-up; above – having average cardiorespiratory fitness in older age related to a 50% greater reduction in risk of becoming dependent over an 8-y period compared to low fit group  Among these older adults physical activity score was not related to subsequent dependence/independence |
| Rantanen et al., 1999 [24]  USA | To determine whether hand grip strength measured during midlife predicts old age functional limitations and disability in initially health men | - N=3218 - Sex: Men - Age: 45-68 y (mean 54 y) with 25 y follow-up to age 71-93 y - Japanese-American, in Honolulu Asia Aging study | Follow-up 25 y  Measurements:  Functional limitation of walking speed below 0.4 m/s, or inability to rise from chair  And Self-reported disability in tasks like lifting, walking and stairs as well as ADL  Physical activity: Hand grip strength grouped by tertiles  Logistic regression models | A clear gradient of increasing risk for all functional limitations and disability outcomes according to weaker baseline hand grip strength tertiles  e.g., for walking speed below 0.4 m/s risk compared to highest grip strength group, in lowest third OR 2.87, CI 1.76-4.67; or inverse OR 0.35, CI 0.21-0.57  and self-care disability usually 2 times greater (i.e., OR 2+ or inverse OR 0.5) in lowest versus highest grip tertile | Hand grip strength predicts functional limitations and disability 25 years later in an initially healthy cohort of men aged 45 to 68  Good muscle strength in midlife may protect people for old age disability by providing a greater safety margin above the threshold of disability |
| Schroll et al., 1997 [30]  Denmark | To highlight easily measured factors that are relevant to prevention and postponement of disability  To describe 5 yr outcome regarding death and functional disability at age 75-80 y as well as individual changes in muscle strength, physical performance in simple function tests and self-reported physical activity and relate risk markers to 5 year outcome | - n=307 survivors (baseline n=405) - Sex: men and women - Age: baseline75 y; follow-up 80 y - Characteristics: Data from the Nordic Research on Aging (NORA) study | Baseline and 5 y follow-up  Measures: Questionnaires/scales, Decline, stability or improvement in mobility function measures of “tiredness” and dependency.  Multiple logistic regression, chi-square | 24% died, 12% did not participate in follow-up  Stability was related to ability to mount stairs, walking speed, mood and physical activity  Mortality was independently related to PA (RR 0.45) increase and muscle strength in women (RR 0.65/N/kg increase)  Follow-up dependency in men was related to low PA at baseline (RR=4.142) and in women (RR=4.32); (Inverse ~ 0.25)  34% of population decreased their PA at follow-up | Mobility dependence was related to low physical activity at baseline  A dose response relationship of declining muscle mass to functional limitations was observed in men and women at the age of 75 y as well as the age of 80 y |
| * Schroll review of papers from this study 2003 [109] | The importance of physical activity for functional ability in old age | N at age 50 y = 802; at age 60 y = 666; at age 70 y =537; at age 80 y= 213; at age 85 y (home visit data only) = 243 from “1914 cohort data”  Sex: Men and Women  Age: Baseline 50 y with follow-up to age 80 y and 85 y  The majority of the population was physically active over the entire life course | Follow-up of 10, 20, 30 and 35 y  Measurements: ADL in 16 activities including 6 related to mobility and rated as to tiredness and help needed  Physical activity: During the last year rated as sedentary to light (less than 2 hr/wk; light (walking cycling gardening) 2-4 hr/wk; light more than 4 hr/wk (brisk, fast, heavy, sports; highly vigorous more than 4 h/wk  Cardiorespiratory fitness measures at 50-60 and 70-80 | Functional abilities in old age were closely associated with previous physical activity i.e., in those who declined approximately 24% were physically active whereas in those who remained stable or improved ~76% were active  Compared to physically active risk of mobility dependence at age 80 was 4.1 in sedentary men and 4.3 in sedentary women; Inverse ~ 0.25 | In relation to cardiovascular diseases and mortality these data point toward a threshold level of approximately 20min/day or 2-3 hr/wk; and in old age this level of physical activity may secure an independent life |
| Stessman et al., 2002 [41]  Israel | To determine the prevalence of independence and ease to perform ADL and IADL and to measure the effect of regular exercise at age 70 y on dependence and ease of performance 7 years later | - n=287 - Sex: Men and women - Age: baseline 70 y, follow-up 77 y - Characteristics: Healthy and sedentary | Follow-up 7 y  Home based interviews and examinations  Questionnaires on ADL and IADL (scale – with ease, some difficulty, considerable difficulty), and voluntary exercise  Physical activity: exercise at least 4 times/wk  Logistic regression | Independence in ADL remained high as did self-reliance in IADL in men  Ease of performance in ADL was independently related to exercise at age 70 y  Females OR 8.5, CI 2.0-36.2; Males OR 4.3, CI 1.1-17.1); Together OR 4.3, CI 1.8-10.6; Inverse OR 0.23 (0.09-0.56)  Ease of performance in IADL correlated to exercise for males OR 3.7, CI 1.1-12.2, but not for women OR 2.0, CI 0.6-6.3 and together OR 2.3, CI 1.1-5.1; Inverse OR 0.43 (0.20-0.91) | Subjects remained active and independent at age 77 y  Exercise at least 4x/w at age 70 y preserved ease of performance (prevalence of independent function in ADL, IADL) at age 77 y |
| Strawbridge et al., 1996 [42]  USA | To determine 6-y predictors of successful aging | - N=356 - Sex: men and women - Age: 65-95 y; mean 77 y | Follow-up 6 y  Measurements: Successful aging – self-reported, having no difficult on any of 13 activity/mobility measures and little or no difficulty on 5 physical performance tasks  Physical activity: reported often walk for exercise  Multiple logistic regression | Physical activity, namely often walks for exercise, was a predictor of successful aging after adjusting for other variables with an OR 0.57, CI 0.35-1.00; inverse of 1.77 CI 1.00-3.12 | Walking (often) for exercise was prospectively associated with successful aging |
| Takkinen et al., 2001 [110]  Finland | To examine longitudinally the predictive value of physical activity for a sense of meaning in life and for self-related health and functioning | - n=198 - Sex: Men and women (72%) - Age: ~75 y with follow up at age 83 y - Characteristics: Participants of the Evergreen Project (1997,1998) | Follow-up: 8 y  Measures: Questionnaires/scales: self-reported health and functional capacity; 5-point Likert-type scale  Physical activity in 6 categories and duration per session  Correlation, GLS, Structural Equation Modeling | Physical activity had a positive effect on meaning in life and self-related health and functioning | Physical activity had a positive effect on both meaning in life and self-related health and functioning  Physical activity also had an indirect effect on self-related health and functioning through meaning in life |
| Unger et al., 1997 [31]  USA | To investigate the impact of social interactions and physical activity on the trajectory of decline in physical functioning of older adults over a 6-year period | - N=5014 - Sex: Men and women - Age: 70+ y; baseline mean 76 y - Part of Longitudinal Study of Aging (LSOA) | Follow-up 6 y  Measurements: Physical functioning – ability to perform ADL, IADL (analyzed as number of impairments)  Physical activity; five self-reported items, including frequency of walking – standardized to a physical activity scale  Individual growth curve analysis | Physical activity was associated with lower levels of impairment and slower functional decline over the 6-y period  Most of the individual physical activity items (compared to peers, compared to previous, frequency of walking one mile) were associated with lower initial levels of disability and slower functional decline | Study found physical activity and social interactions exert independent protective effects on maintenance of functional independence |
| Van Den Brink et al., 2005 [34]  Netherlands Finland, Italy | To investigate the relationship between duration and intensity of physical activity and disability 10 years later | - N=560 - Sex: Men - Age: 70-89 y, mean ~75 y - Disability at baseline excluded - Active group - even lowest group had 40 min per day in walking, cycling, gardening | Follow-up 10 y  Measurements: Disability severity (none, mild, severe) in ADL, IADL and mobility  Physical activity in walking, cycling, gardening; duration and a mean intensity index (code) summing all activities  Logistic regression model | Highest tertile of total physical activity had lower risk of disability vs. lowest tertile; OR 0.46, CI 0.26-0.84; and also middle tertile vs. lowest OR 0.55, CI 0.30 -0.99  Physical activity duration OR 0.42, CI 0.23-0.78; whereas pa intensity not associated with disability | A physically active lifestyle was inversely related to disability, and to prevent disability duration of physical activity seems to be more important than intensity  Duration of 100 min per day was recommended with greater duration having ceiling effect |
| Visser et al., 2005 [48]  USA | To investigate the association between different types of physical activity behaviour and incident mobility limitation | - n=3,075 initially (n=2987 participants) - Sex: Men and women - Age: 70-79 y - Characteristics: Black and White elders enrolled in the Health Aging and Body Composition (Health ABC) study - Only well-functioning   (no initial 400 m walking or 10 stair step limitation) | Baseline and 4.5 year follow-up  Measurements:  Incidence of mobility limitation in 400 m walk or climb 10 steps  Questionnaire on PA previous 7 days, kcal/week; aerobic, weight training; medium and high intensity; walking for exercise  Physical Activity Groups: **Exercisers:** >1,000 kcal/wk of exercise activity (20-30 min moderate on most days)  **Life-style active:** <1,000 kcal/wk with >2,719 kcal/wk of total physical activity  **Inactive:** <1,000 kcal/wk with <2,719 kcal/wk of total physical activity  To assess the effect of walking with or without other exercise activity: Frequent walkers 400 kcal/wk; Occasional walkers <400 kcal/wk; Never walkers  -Thigh muscle area and attenuation; Lean tissue mass; Isokinetic knee extensor strength  ANOVA, Log rank, Cox proportional hazard models | Over 4.5 y, 34.3% of males and 47.4% females developed mobility limitations  Inactive had twice the risk of incident mobility limitation than exercisers (HR 2.08, CI 1.60-2.70 for males and HR 1.98, CI 1.51-2.60 for females); Inverse OR males 0.48, CI 0.30-0.63 females 0.51, CI 0.38-0.66  Life-style active had intermediate risk (HR men 1.47, CI 1.17-1.85; HR women 1.44, CI 1.12-1.84); Inverse OR males 0.70, CI 0.54-0.85 females 0.73, CI 0.57-0.93  For the life-style active and the inactive absence of walking conferred an additional risk of mobility limitation (did not walk HR men 1.72, CI 1.40-2.12; HR women 1.54, CI 1.28-1.84 versus > 400kcal per week walkers; Inverse OR men 0.58, CI 0.47-0.71; OR women 0.65, CI 0.54-0.78 | Exercise and an active lifestyle including walking protect against mobility loss  Physical inactivity doubles the risk of future mobility limitation in those meeting Surgeon General recommendation.  Older persons who had a relatively active lifestyle or who walked regularly had a lower risk than those who were inactive  Walk 1 hr 15 min briskly or 1 hr 40 min moderate to expend 400 kcal/week lowers risk of incident mobility limitation in those who do not participate in moderate to high intensity activities |
| Wang et al., 2002 [43]  USA | To identify factors associated with functional change and investigate inter-actions among selected potential risk factors  Initially ~20% difficulty in ADL or IADL  Unusually active - 70% of sample did regular exercise | - n=2,581 - Sex: Men and women - Age: >65; mean ~ 75 - Characteristics: Participants selected from Group Health Cooperative | Follow-up 3.4 y  Measurements:  ADL; IADL  PPF (performance-based physical function) 4 tests (walking speed, rise from a chair, grip strength, standing balance)  Physical activity questionnaire classed as regular exerciser with 3X/wk or more of 15 activities: walking, hiking cycling aerobic, exercise, etc.  Linear regression, General Estimating Equation | Exercise of 3X/wk or more (and moderate alcohol use) were associated with better functional outcomes and decreased rates of functional decline (less ADL, IADL difficulty, better PPF)  Interactions were seen between exercise and coronary heart disease (CHD), (worse functional outcome with CHD was mitigated among exercisers) | The study identified not only risk factors associated with functional decline but also interactions among these factors |
| Wang et al., 2002 [16]  USA | Identify the benefits of aerobic exercise, running, on disability and mortality in elderly persons and to examine whether morbidity can be compressed by regular exercise | - N=370 runners’ club and 249 controls - Age 59 y at baseline - Sex: Men and women | Follow-up, 13 y  Measurements: Health Assessment Questionnaire disability score; degree of difficulty in 8 daily activities  Runners averaged about 4 hr/wk plus 1 hr vigorous exercise; controls about 90 min of vigorous exercise/wk; also grouped as ever runners vs. never runners  Linear mixed models to compute postponement in disability | Significant difference in progression of disability (HAQ scores) seen between study groups  Reaching a disability score of 0.075 was postponed by 8.7 y (CI 5.5 -13.7 y) in runners vs. controls | Running and other aerobic exercise in older protect against disability and early mortality and are associated with prolongation of a disability-free life |
| *  Chakravarty et al., 2008 [17]  USA | To compare disability and mortality outcomes between cohorts of runners and control subjects initially aged 50 to 72 y  (same sample as Wang et al., 2002) | - N=284 runners; 156 controls - Sex: men and women - Age: baseline 57 y; follow-up 78 y   Controls 1-2 h/wk vigorous exercise; runners 5 hr/wk | 21 y follow-up  Measurements:  HAQ-DI scores  Physical activity: runners versus controls | Runners had a significantly lower risk of HAQ-DI score of 0.5: HR 0.62, CI 0.46-0.84  Runners had survival benefit HR 0.61, CI 0.45-0.82 | Vigorous exercise (running) at middle and older ages is associated with reduced disability in later life and a notable survival advantage |
| Wanna-methee et al., 2005 [21]  UK | Examine association between lifestyle changes in later life (including physical activity) and mobility status (limitation) | - N=5075 - Sex: men   Age 40 to 59 y with follow up at 52 to 73 y (mean 62 y) and 56 to 77 y (mean 66 y) | 12 to 16 y follow-up  Measurements: Mobility limitation – reported difficulty in going out, taking stairs, walking 400 yards (also examined recovery from mobility limitation)  Physical activity score in walking or cycling, recreational activity and vigorous sports – 4 groups stable inactive, became inactive, became active, remained active    Logistic regression | Inverse relationship between physical activity and mobility limitation showed a trend after adjustment for chronic disease (OR of moderately vigorous to none 0.77, CI 0.58-1.03).  Maintaining or taking up physical activity was associated with less mobility limitation; became active vs. stable OR 0.43, CI 0.26-0.71 and becoming inactive showed highest odds of having mobility limitation  Recovery from mobility limitation was increased with physical activity of light and moderate degree Moderate compared to none OR 2.57 (CI 1.31-5.02) Men who remained active were more likely to recover from mobility limitation, OR 3.26 (CI 1.92-5.52) | Maintaining physical activity in later live may improve recovery from mobility limitation  Promotion of light or moderate physical activity program (walking, gardening) |
| Ward et al., 1995 [18]  USA | To examine the development of physical disability in persons older than age 50 y who regularly participated in vigorous exercise | - N=746:Runners =454; Non- runners =292 - Age 50 to 85 y: Runners 58.0 y (83% men); Non-runners 62.3 y (54% men) - Sex: Men and women - Runners were members of runners association | Follow-up 5 to 7 y  Yearly self-report follow-up  Participants classified as runners or non-runners at study entry  Physical disability measured with HAQ-D1  Questionnaire measured prior physical activity levels, current exercise activity, Intensity=min of vigorous exercise per week | Runners averaged 5 hr of vigorous exercise/wk vs. non-runners who averaged 1.5 hr at baseline.  71% of runners were still active runners at end (average decrease of 47 min)  7% of non-runners began running during follow-up time  49% of runners reported some physical disability (average index 0.2) during the study period vs. 77% in non-runners (average index 0.26)  Identified risk factors for disability among runners were presence of arthritis symptoms, older age, greater BMI, strenuous work related activity, and use of greater number of medications | The level of physical disability was generally low. Physical disability, once present, was persistent among both runners and non-runners  Presence of arthritis was greatest risk factor for disability in both groups |
| Wu et al., 1999 [32]  Taiwan | To estimate the incidence of chronic ADL disability and its predictors including leisure-time physical activity | - N=1321 - Sex: men and women - Age: 65+ y; mean ~70 y - Not ADL disabled at baseline | Follow-up 3 y, with annual evaluations  Measurements: Self-report on ADL  Physical Activity: non-active recreational activities and “exercises” (folk dancing, hiking jogging walking at least 2X/wk)  Cox Proportional hazard regression models | Risk of chronic ADL disability was inversely associated with routine exercise, RR=0.52, CI 0.39-0.68 (this association with ADL disability was not found for non-active recreation) | Lack of routine exercise is a significant predictor of ADL disability in older adults |
| Young et al., 1995 [44]  USA | To examine the association of self-reported physical activity with performance based and self-reported physical functioning measured 3 to 5 years later | - n=3640 - Sex: Men - Age: >70 y - Characteristics: Japanese-American older men participation in The Honolulu Heart Program with and without chronic disease | Baseline (1988) and 3-5 year post  Measurements: Timed walks, Balance, Strength, Flexibility, Performance scores, ADL self-reported physical function  Physical activity levels: Self-reported physical activity to estimate METs: Low, Medium, High  Multiple logistic regression, ANCOVA | For healthy subjects those who were highly active initially were more likely to have optimal function for basic ADL OR 2.3, CI=1.1-4.9; Inverse OR 0.43, CI 0.20-0.91  home management skills (OR 1.5, CI=1.1-2.1; Inverse OR 0.67, CI 0.48-0.91)) and physical endurance type tasks OR 1.7, CI 1.2-2.4, Inverse 0.59, CI 0.42-0.83 than low active subjects | Engaging in physical activity is predictive of a high level of physical functioning in older men with and without chronic disease.  In subjects with chronic disease participation in at least moderate PA may be sufficient to maintain optimal physical functioning |

ADL – Activities of Daily Living; ANOVA (ANCOVA) – Analysis of variance (covariance); BMI – Body Mass Index; CI – 95% confidence interval; EPESE - Established Populations for the Epidemiologic Study of the Elderly; FSQ – Functional Status Questionnaire (of ADL and IADL); HAQ-DI – Health Assessment Questionnaire – Disability Index; HR – Hazard ratio; IADL – Instrumental Activities of Daily Living; OR – Odds Ratio; LTPA – Leisure Time Physical Activity; MET – Resting metabolic equivalent; PPT – Physical Performance Test; RR – Relative Risk; %ile – percentile.

* Indicates second report from the same study/data base with different variables, analysis or period of follow-up. These were considered as supplementary reports not counted as “separate” studies.
