# Supplementary material for: Physical activity and functional limitations in older adults: a systematic review related to Canada's Physical Activity Guidelines
Source: Int J Behav Nutr Phys Act. 2010 May 11;7:38. doi: 10.1186/1479-5868-7-38 (PMC2882898; doi:10.1186/1479-5868-7-38)
Supplement: Additional file 3 — Supplemental table 5. Table s5: Prospective studies assessed with the modified Downs and Black Quality Assessment Tool. [file 1479-5868-7-38-S3.DOC]

| **Publication** | **Reporting (7)** | **External Validity (1)** | **Internal Validity (4)** | **Total (12)** |
| --- | --- | --- | --- | --- |
| Al Snih et al., 2004 | 6 | 0 | 4 | 10 |
| Bäckmand et al., 2006 | 6 | 0 | 3 | 9 |
| Berk et al., 2006 | 6 | 0 | 4 | 10 |
| Boyle et al., 2007 | 6 | 0 | 3 | 9 |
| Buchman et al., 2007 | 5 | 0 | 4 | 9 |
| Brach et al., 2003 | 7 | 0 | 4 | 11 |
| Brach et al., 2003 | 4 | 0 | 4 | 8 |
| Bruce et al., 2008 | 5 | 0 | 4 | 9 |
| Christensen et al., 2006 | 6 | 1 | 4 | 11 |
| Clark, 1996 | 4 | 1 | 3 | 8 |
| Dunlop et al., 2005 | 6 | 0 | 2 | 8 |
| Ebrahim, et al., 2000 | 5 | 0 | 3 | 8 |
| Ferrucci et al., 1999 | 6 | 0 | 3 | 9 |
| Giampaoli et al., 1999 | 6 | 0 | 3 | 9 |
| Haight et al., 2005 | 6 | 0 | 4 | 10 |
| Tager et al., 2004 | 6 | 0 | 4 | 10 |
| Haveman-Nies et al., 2003  (also reviewed in Haveman-Nies et al., 2003, Age and Ageing) | 5 | 0 | 4 | 9 |
| Hirvansalo et al., 2000 | 7 | 1 | 3 | 11 |
| Huang et al., 1998 | 5 | 0 | 4 | 9 |
| Koster et al., 2008 | 5 | 0 | 4 | 9 |
| Koster et al., 2007 | 5 | 0 | 4 | 9 |
| Lang et al., 2007 | 6 | 1 | 3 | 10 |
| Leveille et al., 1999 | 5 | 0 | 4 | 9 |
| Ǿstbye et al., 2002 | 6 | 1 | 3 | 10 |
| Paterson et al., 2004 | 7 | 1 | 4 | 12 |
| Rantanen et al., 1999 | 6 | 0 | 4 | 10 |
| Schroll et al., 1997 | 6 | 1 | 3 | 10 |
| Stessman et al., 2002 | 5 | 0 | 3 | 8 |
| Strawbridge et al., 1996 | 6 | 0 | 3 | 9 |
| Takkinen et al., 2001 | 7 | 0 | 3 | 10 |
| Unger et al., 1997 | 5 | 1 | 4 | 10 |
| Van Den Brink et al., 2005 | 6 | 0 | 4 | 10 |
| Visser et al., 2005 | 6 | 1 | 4 | 11 |
| Wang, BWE et al., 2002 | 5 | 1 | 3 | 9 |
| Wang, L et al., 2002 | 7 | 0 | 4 | 11 |
| Chakravarty et al., 2008 | 5 | 0 | 4 | 9 |
| Wannamethee et al., 2005 | 7 | 0 | 4 | 11 |
| Ward et al., 1995 | 6 | 1 | 3 | 10 |
| Wu et al., 1999 | 6 | 0 | 3 | 9 |
| Young et al., 1995 | 5 | 0 | 3 | 8 |
