# Supplementary material for: Physical activity and functional limitations in older adults: a systematic review related to Canada's Physical Activity Guidelines
Source: Int J Behav Nutr Phys Act. 2010 May 11;7:38. doi: 10.1186/1479-5868-7-38 (PMC2882898; doi:10.1186/1479-5868-7-38)
Supplement: Additional file 4 — Supplemental table 6. Table s6: Aerobic or combined exercise training studies examining the relationship between physical activity and functional limitations in older adults [111-121]. [file 1479-5868-7-38-S4.DOC]

| **Publication**  **Country** | **Objective** | **Population** | **Methods** | **Outcomes** | **Comments and Conclusions** |
| --- | --- | --- | --- | --- | --- |
| Bowen et al., 2006 [111]  Canada  RCT | To determine the short (3 month) and long term (12 month) effects of an intensive exercise program on quality of life (QOL) and functional status | - n=157 - Sex: Female - Age: 50-75 y (mean 61 y) - Exercise Group: n=87 - Control Group: n=86 - Characteristics: Sedentary (<60 min of moderate/vigorous exercise per week, <25.0 ml/kg/min VO_2max_) middle-aged postmenopausal women | Baseline, 3 and 12 months  **Exercise group (EG)**:  45 min of at least moderate intensity aerobic exercise 5 times/week for 12 months. Starting at 40% VO_2max_ for 16 min per session, increasing to 60-75% VO_2max_ for 45 min sessions by week 8 and then maintained for the rest of the study  Exercise included: Treadmill walking, aerobics, stationary cycling, light weights  **Control group (CG)**  Measurements:  -SF-36 Health Survey  -Brief Symptom Inventory (BSI)  -MOS Social Support Survey  -Scales  -VO_2max_  Checklist  GEE, linear regression | EG showed changes in general health at 3 and 12 months and better scores at both time points than the CG  EG showed an improved change (within group p=0.13) in physical functioning (SF36) at 12 months vs. CG who showed decreased (within group p=0.02) functioning at 12 months (between groups p=0.01) | Moderate to intensive exercise had some positive and no negative effects on improving functioning  The positive effects of exercise programs could support long-term adherence to the exercise regime |
| Buchner et al., 1997 [68]  USA  RCT | To determine the effect of strength and endurance training on gait, balance, physical health status, fall risk, and health services use in older adults | - n=105 - Sex: Male and Female - Age: 68-65 y (mean 75 y) - Endurance training (ET): n=25, 52% female, mean age 75 y - Strength Training(ST): n=25, 52% female, mean age 74 y - ET&ST: n=35, 52% females, mean age 75 y - CG: n=30, 50% female, mean age 75 y - Characteristics: part of the FICSIT study; unable to do 8-step tandem gait without errors; below 50^th^ percentile in knee extensor strength | Baseline, 6 months, 9 months (exercise groups unsupervised exercise outcome measures)  4 Groups:  3 days/week, 1hr  10-15 min warm-up; 5-10 min cool down.  Endurance Training (ET)  30-35 minutes of cycle exercise (75% heart rate reserve)  Strength Training (ST)  2 sets, 10 reps, 50-60%1RM, 75%1RM, upper and lower body exercises  Endurance & Strength (ET&ST)  20 min cycling, 1 set strength training at 75%1RM  Control (CG)  Instructed to maintain normal activities  Measurements:  VO_2_max  Balance  Gait speed  Stair climbing speed  Sickness Impact Profile  SF-36  **Analysis**:  Repeated measures ANOVA  Paired t-tests | Aerobic capacity increased in the ET (p<0.05) and ET&ST (p<0.01) groups at 9 months only.  ST showed significant increases in isokinetic strength at 6 months in all muscle groups except ankle. Knee extension strength increased in the ET group.  No effect of exercise on gait, balance, or physical health status | Exercise may have beneficial effects on fall rates and health care use in some subgroups of older adults.  In community-living adults with mainly mild impairments in gait, balance, and physical health status, short-term exercise may not have a restorative effect. |
| Cress et al., 1996 [111]  USA  Non-RCT | To evaluate the changes of muscle  ultrastructure, muscle strength, and whole body functional performance as a result of a functionally directed exercise program (stair climbing). | - n= 13 - Sex: Female - Age: 65 to 83 y - Characteristics: Healthy | Baseline, 50 weeks  Exercise Group (EG):  Weighted stair climbing (10% of body weight) provided resistance to the legs. Each subject ascended and descended the 24 stairs eight times per session. Endurance dance (30 minutes, 75% HRR) upper body resistive exercises (elastic tubing) (20 minutes combined with stair walking).  3 times/week, total 60 minutes  **Control Group**:  Measurements:  Vastus lateralis biopsy  Isokinetic thigh strength  Maximal attainable stair riser height  **Analysis:**  Paired t test. Pearson correlation, ANOVA | Changes in myofibrillar area accounted for 48% of variance in muscle strength changes.  Change in muscle contractile protein was underlying basis for change in thigh strength which was significantly (F = 10.23; *p* = .003) different for the lowest to the highest riser height (28.0 + 1.0,  43.6 + 4.9; 47.0 + 9.6, respectively),  with strength accounting for 65%  (eta2 = .6509) of the performance | The adaptations of the  vastus lateralis as indicated on the  ultrastructural and fiber level were  reflected in increased strength,  which, in turn, was translated into  improved whole body performance as measured by stair climbing, that is important for independent living |
| Cress et al., 1999 [112]  USA  RCT | To evaluate exercise for significant and meaningful improvements in physical functions not detected by commonly used measures of physical function | - n=49 - Sex: Male and female - Age: >70 y, mean 76 y - Exercise Group: n=23, mean age 75.6 y - Control Group: n=26, mean age 76.0 y - Characteristics: Healthy independent older adult living in retirement communities or apartments | Pre, post 6 months  2 groups:  **Exercise (EG)**  3 times/wk for 60 min (10 min warm-up and cool down and 50 min training)  Strength Training: 75%-80% of 1RM Endurance training: 75%-80% HRR  Stair climber, semi-recumbent single leg press, stair climber, kayak machine  **Control (CG)**  Measurements:  Questionnaire CS-PFP  VO_2max_  Gait, balance reaction time  Strength (elbow, knee flexion)  ROM  SF-36  Sickness Impact Profile (SIP)  6 min walk  Analysis:  ANOVA, ANCOVA | EG showed an increase in VO_2max_ (11%), strength (33%) CS-PFP total (14%) and CS-PFP domains (endurance 20%, upper body strength 13%, lower body strength 14%). p<0.02  Participants in EG with lower CS-PFP scores at base-line showed greater functional changes  No differences were found between groups for SIP, SF-36 or 6 min walk | Strength and endurance training cause an increase in muscle strength and aerobic capacity  Independent older adults gain meaningful benefits from several months of exercise training. The importance of physical activity may relate not just to its role in preventing decline but also to its role in enhancing physical functioning |
| Davidson et al., 2009 [69]  Canada  RCT | To investigate the independent and combined effects of resistance and aerobic exercise on insulin resistance and functional limitations in older men and women | - n=117 - Sex: Male (n=57) and Female (n=79) - Age: 60-80 y - CG: n=28 (11M,17F) - RE: n=36 15M, 21F) - AE: n=37 (17M,20F) - CE: n=35 (14M,21F) - Characteristics: Abdominally obese, sedentary | Baseline, 6 months  4 Groups:  **Control (CG)**  **Resistance Exercise (RE)**  3 times/week, 60 min weekly, 1 set to volitional fatigue (weight increased after 15 good form reps) of chest press, shoulder raise and flexion, leg extension and flexion, triceps extension, biceps curl, abdominal crunches, modified push-ups.  **Aerobic Exercise (AE)**  5x/week, 30min of moderate intensity (60-75% VO_2peak_) treadmill walking  Combined Exercise (CE)  3x/week, RE and AE protocols, 150 min/week.  Control Group (CG)  Measurements:  VO_2peak_  Chair rise  2-minute step  8-ft- up and go  Seated arm curl  **Analysis**:  ANCOVA | 91% adherence  Functional limitations improved significantly in all exercise groups for all tests versus controls.  Improvement within the CE was greater than that in the AE (0.52 [0.10] vs. –0.01 [0.10] standard units, *z* score [p=0.003)]), but not RE. Improvement in the RE was not different from the AE. | Combination of resistance and aerobic exercise was the optimal exercise strategy for simultaneous reduction in insulin resistance and functional limitation in previously sedentary, abdominally obese older adults |
| Fisher and Li, 2004 [114]  USA  RCT (Neighbour-hoods) | To evaluate the effects of a neighbourhood walking program on quality of life in older adults | - n=582 - Sex: Male (n=182) and female (n=400) - Age: >65 y - Ethnicity: 82% White - Characteristics: Sedentary, low income community dwelling seniors   Neighbourhoods (n=56) | Baseline, 3 and 6 months  Two groups:  **Intervention group**  6 months 3 times/week, leader-led walking for ~1hour.  Warm-up and cool down with 30-40 min of leisurely but purposeful walking  **Control group**  Information only  Quality of life measures:  Short Form –12 (SF-12)  (8 items including physical functioning)  Satisfaction with life scale (SWLS)  Walking activity=frequency of neighbourhood walking  Multilevel longitudinal analysis. Multilevel latent curve analysis | Significant improvements in SF-12 physical in intervention (mean slope [M=1.00] was significant [p<0.001]) vs. Controls  Training score 66 to 72  Control score 66 to 65  Significant increase in walking activity in intervention neighbourhoods (p<0.05) versus control neighbourhoods | A neighbourhood based walking program is beneficial for promoting quality of life in seniors |
| Kalapotharakos et al., 2006 [115]  Greece  RCT | To determine the effect of a 12 week aerobic exercise program on functional and neuro-motor performance in inactive healthy older adults | - n=22 - Sex: Male and female - Age: 60-75 y mean age ~61y - Characteristics: Healthy and sedentary | Pre and post 12 weeks  Two groups: **High intensity aerobic exercise (AE);** 3 times/week for 12 wks on non-consecutive days Progressive increase; 5 min warm-up, 5 min cool down, 40% of HRmax – 40% HRmax, 20 min - 40 min  **Control group (CG)**  Measurements: 1RM knee extensors and flexors, 6 min walk distance, chair-rising time, whole body reaction time  ANOVA with RM | AE had an improvement in: 1RM knee extensors (12%); Flexors (19%); 6 min walk (17%); Chair rising time (8%)  No differences were observed in CG | Short term progressively increased high intensity aerobic exercise improved the physical and neuro-motor performance in inactive healthy older adults  Suggesting that participation in progressively increased high intensity aerobic exercise program may improve mobility and ability to carry out ADL in older adults |
| Kawanabe et al., 2007 [116]  Japan  Non-RCT | To determine the beneficial effect of whole-body vibration exercise in addition to muscle strengthening, balance, and walking exercise on the walking ability in the elderly | - n=67 - Sex: Male and female - Age: 59-86 y (mean 72 y) - Characteristics: Healthy and low active elders | Baseline and 2 month follow-up  2 groups:  **Whole-body vibration (WBV)** plus routine exercise  Performed on a Galileo machine at 12-20 Hz for 4 min, 1 time/wk. Plus routine exercise, balance, muscle strength training and walking 2 times/week for 30 min  **Routine exercise group (RE)**  Balance standing on one leg and tandem gait, muscle strength training of the calf, quadriceps, hamstrings and gluteus medius and walking 2 times/wk for 30 min  Measurements:  10-m walking time  Step length  Maximum standing time on 1 leg  t-test, correlations, ANOVA | WBV improved the 10-m walking time (by -14.9%), step length and maximum standing time on one leg  Right side  Step- length and standing time improved by 6.5% and 65%  Left side  Step- length and standing time improved by 6.5% and 88.4%  There were no changes in the RE group | The study showed a beneficial effect of whole-body vibration exercise in addition to muscle strengthening, balance, and walking exercise in improving the walking ability in the elderly |
| King et al., 2000 [117]  USA  RCT | To compare effects of two physical activity programs on measured and perceived physical functioning and QOL | - n=103 (36 male; 67 females) - Sex: Male and female - Age: >65 y - Ethnicity: 95%Caucasian - Characteristics: Healthy and sedentary | 12 months of activity Classes and home-based  Pre and post measures  2 groups (sub-divided into males and females):  **Fit and firm (FF)**  5-10 min warm-up, 40-50 min moderate intensity endurance and strength training, 5-10 min cool-down  THR 60-75% HRR  **Stretching and flexibility (SF)**  5-10 min warm-up, 40 min stretching, 5-10 min relaxation exercises  Measurements:  -Function cap:  VO_2max_  Functional tasks  -Self related/perceived:  3 scales  ANOVA, ANCOVA, MANOVA | FF showed greater improvements in measured and self-rated endurance and strength compared to SF  FF showed greater improvements in submax HR (*F*4,91=3.59, *p*<.03)  No difference between groups in VO_2max_ and all groups improved VO_2max_ with the exception of women in SF | Physical activity regimens focusing on moderate intensity endurance and strengthening exercises or flexibility exercises result in improvement in functional and QOL outcomes |
| Nakamura et al., 2007 [118]  Japan  Non-RCT | To evaluate the effects of exercise frequency on functional fitness | - n=45 (exercise groups n=34; control group n=11) - Sex: Female - Age: Mean 67.8 y Characteristics: Older sedentary women | Pre and post 12 weeks  3 exercise groups:  Exercise, 10 min warm-up, 20 min walking, 30 min recreational activity, 20 min resistance training 10 min cool-down  **Group I**  90 min exercise 1 time/wk  **Group II**  90 min exercise 2 times/wk  **Group III**  90 min exercise 3 times/wk  **Control group**  Measurements: IADL,  Strength, endurance, balance, coordination and cardio-respiratory fitness (6 min walk)  ANOVA | Greater improvement in cardio-respiratory fitness, coordination, endurance and balance for group III vs. I and II  6-min walk: significant in 3 times/wk vs. other groups; ~4% distance increase | Older women who participate in an exercise program 3 times/week gain greater functional fitness than those who exercise less |
| Pahor et al., 2006 [67]  USA  RCT | To examine the effects of a physical activity intervention on Short Physical Performance Battery (SPPB) and other physical performance measures | - n=424 - Sex: Male and female - Age: 70-89 y mean 76.5 y - Characteristics: Healthy and sedentary | Lifestyle Intervention and Independence for Elders pilot (LIFE-P)  Baseline 6 and 12 months  2 groups:  **Physical activity (PA)**  Walking, strength, flexibility and balance training with behavioural skills  Weeks 1-8, 3 times/week  Weeks 9-24, 2 times/week  Weeks 25 up, 1 time/week  **Educational control (SA)**  Health education workshops  Weekly, 1-26 weeks  Monthly from week 27 up  Physical function measure:  Short Physical Performance Battery (SPB)  General linear model | At 6 and 12 months PA had higher scores in SPPB than SA  PA had lower incidence of major mobility disability (incapacity to complete a 400m walk) OR, 0.71, 95% CI=0.44-1.20 | A structured PA intervention improved the SPPB score and other measures of physical performance |
| (Supplemen-tary report)  Fielding et al., 2007 [119]  USA  RCT | To examine the role of adherence to physical activity in the magnitude of improvement in physical functioning | - n=327 - Sex: Male and Female - Age: 70-89 y; mean 76.5 y - PA: n=116 - SA: n=211 - Characteristics: Participants of the LIFE-P (healthy, sedentary) | 4 sites, baseline, 6 months, 12-18 months  **2 Groups:**  **Physical Intervention Group (PA)**  3 phases: adoption (1-8wks); transition (9-24wks); maintenance (25wks to end of trial)  Progression from centre-based activity to home-based activity of 5 + times/week walking program (moderate intensity [RPE 13] with goal of 150 min/wk) with strength (10min of lower-extremity exercise with ankle weights (RPE 14), balance, and stretching.  **Successful Aging Group (SA**)  Health education attention-control program for 1y  **Measurements:**  Adherence  Short Physical Performance Battery  **Analysis**:  Linear regression, Wilcoxon rank-sum test, mixed-effects general linear model | Significant difference in moderate activity between PA and SA at 6 and 12 months;  PA increased physical activity from baseline to months 6 (+ 102 min) and 12 (+63 min) (p<0.001).  PA participants who reported >150 min/wk of activity showed a significantly greater improvement in SPPB vs. those who reported <150min/wk (p<0.017) at 12 mo (but not at 6 mo) | Adherence to physical activity in the LIFE-P was associated with greater improvement in SPPB score. |
| Pereira et al., 1998 [66]  USA  RCT at baseline | To conduct a 10 year follow-up of physical activity (PA) and self-reported health status in participants of a randomized control trial (RCT) of walking intervention | - n=196 - Sex: Female - Age: Baseline, walkers 57.8 y; controls 57.2 y   Follow-up  walkers 70.9 y; controls 70.3 y   - Characteristics: Postmenopausal women at baseline | 10 year follow-up telephone interview  2 groups  **Intervention group**  Walking exercise  80% of women averaged >5 miles/week over the first 2 years, with 61% averaging >7 miles/week  **Control group**  ***Intervention is not reported in this study**  Follow-up questions on:  -Self-reported walking – to calculate weekly estimate (kcal/wk)  -Paffenbarger sport and exercise index  -Functional status  -Chronic diseases and conditions  t-test, Wilcoxon rank sum test, chi-square | At follow-up the intervention group (walkers) had significantly higher usual walking and total walking weekly energy expenditure vs. controls (1344 vs. 924 kcal/wk; 1008 vs. 302 kcal/wk *p=0.01* respectively) | The study demonstrated long-term exercise compliance in older women and suggested that health benefits may have ensued as a results of increased activity level |
| Puggard et al., 2003 [120]  Denmark  Non-RCT | To elucidate if regular physical training may influence the expected decline in physical functional ability in three age cohorts. | - n=128 - Sex: Female - Age: 3 age groups: 65 y (n=19); 75 y (n=54); 85 y (n=55). - Characteristics: Healthy | Baseline, 8 months  4 Groups:  65y, 75y, 85y, control group.  Training:  65 and 85: 1 time/week, 60 min  75: 2 times/week, 60 min  Walking (72%, 62%, 69% HR_max_ for 65 y, 75 y, 85 y respectively.)  Measurements:  PPT, VO_2max_, isometric strength (trunk, leg/hip muscles)  30m walking test  Analysis:  ANOVA, Wilcoxon Mann Whitney | No significant increases in VO_2max_. There was a significant decrease in VO_2max_ in 75 and 85y groups.  No significant increases in strength.  All three groups significantly decrease walking time (p<0.05).  The 85 y group increased PPT total score. | Regular training can improve physical ability of older women with regard to PPT, VO_2max_, and maximal walking speed. Suggesting both young-old and old-old women are able to benefit from regular tailored exercise training. |
| Toraman et al., 2004 [121]  Turkey  RCT | To evaluate the effects of a 9-week supervised multi-component exercise programme on functional fitness and body composition in independent older adults | - n=42 (CG=21; EG=21) - Sex: Male and female - Age: >60y - Characteristics: Healthy | Baseline, 9 weeks  2 Groups:  Exercise Group (EG):  Aerobic, resistance and flexibility exercises  -50% HRR for 20 min 3 times/week with 5 min and 5% increase every 2 weeks  -50% of predicted 1RM increasing to 80%  -static stretching major muscle group  Control Group:  Measurements:  Senior fitness test, 6 items  Up and go, chair stand, arm curl, 6 min walk, chair sit and reach, back scratch  Analysis:  t-test, ANOVA, ANCOVA | Significant time and group interaction effects for the arm curl (*p* = .002); chair stand (*p <* .001); 8-ft up-and-go (*p* = .002); 6-min walk (*p <* .001).  No significant effect the back scratch (*p* = .016); chair sit-and-reach (*p* = .918); BMI, ( *p* = .203); percentage body fat (*p* = .238); fat-free mass ( *p* = .278); and waist:hip ratio (*p* = .31 | A short-term mul­ti-component exercise program, older adults who had low physical activity levels or even medically stable chronic diseases can improve their functional fitness, primarily in terms of upper and lower extremity strength, 6-min walk, and agility or dynamic balance.  Toraman and Sahin (2004) also reported similar gains based on young-old (60-73 y) and old-old (74-86 y) age-groups  As reported by Toraman and Ayceman (2005) changes in lower extremity flexibility, up and go and 6 min walking in response to 6 weeks detraining are affected by age in a follow-up study of 21 “training” group subjects (decline in older group 74-86 y vs. younger group 60-73 y) |

ADL – Activities of Daily Living; ANOVA (ANCOVA) – Analysis of variance (covariance); IADL – Instrumental Activities of Daily Living; Hz- Hertz; HR - Heart rate; HRmax - Maximal heart rate; HRR - Heart rate reserve; kcal/wk - Kilocalories per week; m - metres; MANOVA - Multivariate analysis of variance; MET – Resting metabolic equivalent; PPT - Physical performance test; QOL - Quality of life; reps - repetitions; ROM - Range of motion; RM - Repetition maximum; RPE - Rating of perceived exertion; SPPB - Short physical performance battery; VO_2max_ (ml/kg/min) - Maximal oxygen uptake (millilitres per kilogram per minute); W - Watt.
