# Supplementary material for: Physical activity and functional limitations in older adults: a systematic review related to Canada's Physical Activity Guidelines
Source: Int J Behav Nutr Phys Act. 2010 May 11;7:38. doi: 10.1186/1479-5868-7-38 (PMC2882898; doi:10.1186/1479-5868-7-38)
Supplement: Additional file 5 — Supplemental table 7. Table s7: Aerobic or combined exercise studies assessed with the modified Downs and Black Quality Assessment Tool. [file 1479-5868-7-38-S5.DOC]

| **Publication**  **Country** | **Reporting**  **(11)** | **External Validity**  **(2)** | **Internal Validity**  **-Bias (6)** | **Internal Validity -Confounding (5)** | **TOTAL (24)** |
| --- | --- | --- | --- | --- | --- |
| Bowen et al., 2006  Canada | 6 | 0 | 5 | 5 | 16 |
| Buchner et al., 1997  USA | 10 | 0 | 6 | 5 | 21 |
| Cress et al., 1999  USA | 8 | 0 | 5 | 5 | 18 |
| Davidson et al., 2009  Canada | 11 | 0 | 6 | 5 | 22 |
| Fisher and Li, 2004  USA | 10 | 2 | 5 | 6 | 23 |
| Kalapotharakos et al., 2006  Greece | 8 | 0 | 5 | 4 | 17 |
| King et al., 2000  USA | 11 | 2 | 5 | 6 | 24 |
| Pahor et al., 2006  USA | 10 | 1 | 6 | 5 | 22 |
| (Supplementary report)  Fielding et al., 2007  USA | 9 | 1 | 6 | 5 | 21 |
| Pereira et al., 1998  USA | 10 | 1 | 5 | 5 | 21 |
| Toraman et al., 2004  Turkey | 10 | 2 | 5 | 5 | 20 |

| **Publication**  **Country** | **Reporting**  **(11)** | **External Validity**  **(2)** | **Internal Validity**  **-Bias (5)** | **Internal Validity -Confounding (5)** | **TOTAL (23)** |
| --- | --- | --- | --- | --- | --- |
| Cress et al., 1996  USA | 9 | 1 | 5 | 4 | 19 |
| Kawanabe et al., 2007  Japan | 6 | 0 | 5 | 4 | 11 |
| Nakamura et al., 2007  Japan | 9 | 0 | 5 | 4 | 18 |
| Puggard et al., 2003  Denmark | 7 | 1 | 5 | 4 | 17 |
