# Supplementary material for: Physical activity and functional limitations in older adults: a systematic review related to Canada's Physical Activity Guidelines
Source: Int J Behav Nutr Phys Act. 2010 May 11;7:38. doi: 10.1186/1479-5868-7-38 (PMC2882898; doi:10.1186/1479-5868-7-38)
Supplement: Additional file 6 — Supplemental table 8. Table s8: Resistance/strength or functional training studies examining the relationship between physical activity and functional limitations in older adults [122-130]. [file 1479-5868-7-38-S6.DOC]

| **Publication**  **Country** | **Objective** | **Population** | **Methods** | **Outcomes** | **Comments and Conclusions** |
| --- | --- | --- | --- | --- | --- |
| Ades et al., 1996 [122]  USA  RCT | To determine the effect of a resistance training program on walking endurance | - n=24 - Sex: Male and female - Age: >65 y (mean 70.4 y) - Characteristics: Healthy sedentary community dwelling elders | Baseline and post-12 week  2 Groups:  **Resistance training group (RTG)**  7 exercises at 3 sets of 8 reps with a rest period of 1-2 min between sets for 3 days/wk at 80% 1-RM  **Control group (CG)**  Did not alter their home activity habits  Measurements:  -VO_2peak_  -Body composition – underwater weighing  -Strength  -Sub-maximal endurance capacity (treadmill test at 80% peak until exhaustion or 45 min max)  ANOVA with repeated measures, regression | Sub-maximal walking endurance increased in the RTG from 25±4 min to 34±9 min (*p*=0.001)  No change was seen in the CG  There was a significant relationship between change in leg strength and change in walking endurance r=0.48 (*p*=0.02) | Resistance training for 3 months improves both leg strength and walking endurance |
| Ashmead and Bocksnick, 2002 [123]  Canada  Non-RCT | To improve the functional ability of the older adult by improving muscular fitness through a home-based intervention | - n=10 - Sex: Female only - Age: 57-78 y (mean 66.4 y) - Characteristics: Healthy female subjects | Pre and post 10 weeks  Instructional exercise videos produced specifically for the study  A 1 hour instructional seminar prior to commencement  Exercise:  3 times/wk for 25 min  3-5 min warm-up, 2 rounds of strength training circuit, 3-5 min cool-down  Measurements:  Max hand grip strength  6 muscular endurance tests  Questions on activities of daily living (ADL)  Dependent t-test | Improvements in max hand grip strength of the right hand (*p*<0.05)  Improvements in all 6 muscular endurance tests    Participants reported vast improvement **in functional abilities** of ADL | An age-specific home-based strength training and endurance program can improve fitness among older adults and improve functional abilities |
| Bean et al., 2004 [124]  USA  RCT | To evaluate a dynamic form of weighted vest exercise suitable for home use and designed to enhance muscle power, balance, and mobility | - n=18 - Sex: Female - Age: >70 y - Characteristics: Healthy, SPPB score between 4 and 10 | Baseline, 12 weeks  2 Groups  3 times/week, 30 min, 3 sets of 10 reps  **Increased velocity exercises specific to task (InVEST):**  Dynamic progressive resistance upper and lower body exercises using a weighted vest  **Control group**:  Slow-velocity, low-resistance exercises consisting of chair-based  exercises that involved upper and lower body muscle groups using body or limb weight for  resistance  Measurements:  Leg power and strength (1RM)  SPPB  SF 36  Analysis: t-tests | InVEST group showed significant improvements (p>0.05) in leg power across measurements obtained at 75% to 90% of the 1 RM. Both groups demonstrated significant improvements in chair stand and SPPB score from baseline,  and the InVEST group showed significant improvements in  gait speed and chair stand from baseline (P< 0.05). InVEST produced significantly greater changes in chair stand time vs. control (P>0.05). | InVEST training appears be an effective means of enhancing leg power and chair rise in this population and is worthy of further investigation as a means of enhancing balance and mobility |
| Bunout et al., 2001 [125]  Chile  RCT | To assess the impact of an 18 month nutritional supplementation and resistance training program on health functioning of elder adults | - n=98   (SE; n=31, SN; n=26, NE; n=16, NN; n=25)   - Sex: Male and female - Age: >70 y | Baseline, 6, 12 and 18 months  Four groups:  Supplemented and trained (SE)  Supplemented and not trained (SN)  Not supplemented but trained (NE)  Not supplemented or trained (NN)  Resistance exercise training:  1 hour twice a week  15 min walking followed by chair stands, modified squats, step-ups, therabands pull-ups and 15 min walking  Measurements:  Katz ADL score  ANOVA | Elders who were receiving supplementation and resistance training maintained functionality | An 18 month supplementation and resistance training program for the elderly avoided deterioration of activities of daily living |
| Brandon, 2000 [76]  USA  RCT | To evaluate the effects of a 4 month lower extremity strength-training program on functional mobility in older adults | n=85  Age: mean 72.3 y  Sex: Male and Female  ES: n=43 (mean age 72 y, 15M, 28F)  CS: n=42 (mean age 72.6y, 10M, 32F)  Characteristics: Healthy, no structured physical activity | Baseline, 2, 5, 7, 12, 16 weeks  **2 groups**:  Experimental (ES)  1hr, 3 days/week  3 sets (50%, 60%, 70% 1RM), 8-12 reps.  Knee flexors and extensors, plantar flexors  10 min stretching  Control (CS)  Measurements:  1RM (absolute and relative): knee flexors and extensors, plantar flexors;  Physical Performance Test (PPT) with addition of floor rise  Analysis:  2x2 factorial ANOVA, stepwise regression | 95% training and retest adherence  Both groups showed strength fitness greater than peers in age group at baseline  ES showed significant increase in absolute (lower extremity ratio increased 51.7%) and relative 1RM for all 3 muscle groups  Plantar flexors showed greatest improvement  Only chair rise and floor rise showed significant improvement in ES post-training (p<0.05). | Among reasonably fit older adults, increasing strength above baseline provides marginal improvements in mobility |
| Buchner et al., 1997 [68]  USA  See Table 6 | See Table 6 | See Table 6 | See Table 6 | See Table 6 | See Table 6 |
| Capodaglio et al, 2007 [71]  Italy  Non-RCT | To determine the impact of a 1 year mixed strength training programme on muscle function, functional ability, physical activity and lifestyle | - n=38 Sex: Male and female - Age: 70-83 y (Males mean 76.6 y, females mean 77.5 y) - Ethnicity: Italian - Characteristics: Healthy elderly community dwelling | Baseline, 1 year follow-up  2 groups:  **Training (T)**  60% of 1-RM on resistance machines  Initially 40%1-RM increasing to 60% 1-RM  Duration ~60 min including warm-up and 10 min cool-down with stretches  1 time/wk home exercise using Therabands  **Control (C)**  Did not engage in the physical activity program  Measurements:  Questionnaires  -Aerobic activities over 3 METs (AA3)  -Mean daily energy expenditure (MDEE)  -Muscle function, max strength of knee extensors (KE) and plantar flexors (PF)  -Functional abilities, reach, chair rise, bed rise, 6 min walk, stair climbing, get up and go, 1 leg standing  ANOVA 2 factor (gender, training), MANOVA, correlation | Gains in muscle function and functional ability in both training females and males with females improving more than males  Trained males increased physical activity time by 146% and trained females by 16%  MDEE increased by 10% in trained males | A long-term mixed programme can improve muscle functional ability in older females and functional abilities in males |
| Cavani et al., 2002 [72]  USA  Non-RCT | To determine the effect of 6 weeks of stretching and moderate-intensity resistance training on functional fitness | - n=37 - Sex: Male and female - Age: 60-79 (y EG mean 69 y, CG mean 70 y) - Characteristics: Healthy and either sedentary or moderately active | Pre post 6 weeks  2 groups:  **Exercise (EG)**  3 times/wk for ~45 min, 1 set 12-15 reps on resistance machines and 20 min stretching  **Control (CG)**  Asked not to change their physical activity routine or join an exercise program  Test battery (Rikli & Jones 1999) – 6 tests  t-test, two-way ANOVA with RM | The EG had improvements on all the tests except 6 min walk  The relative number of reps during arm-curl and chair stand, increased by 24%, 30%, respectively  Time for up and go decreased by 15% and distance for 6 min walk increased by 9% (non-significant)  Absolute changes in back scratch and sit and reach improved by 1.3 in and 2.28 in, respectively | Moderate-intensity resistance training in conjunction with stretching can improve functional fitness enabling them to more easily perform ADL |
| Earles, 2001 [77]  USA  RCT | To test the efficacy of medium- and high-velocity power training in older persons | n=40  Sex: Male and Female  Age: >70 y  PG: n=18, mean age 77 y 64% women  CG: n=22, mean age 78 y, 68% women  Characteristics: Healthy | Baseline, 12 week follow-up  **2 Groups**:  Control Group (CG)  Recommended 30 min walking 6 days/week  Power Training Group (PG)  3 days/week  Brief warm-up and stretching  3 sets of 10 repetitions (2 sets for leg press) of leg press, hip flexion, step-ups, chair rises, plantar flexion  Use of weight belts  Additional 45 min moderate walking per week  Measurements:  1RM leg press  Short Physical Performance Battery  Balance  Chair rise  8 Foot Walk  Single leg stance  6 min walk  **Analysis**:  Repeated measures ANOVA | PG increased maximum leg press (22%, p<0.004)  150% increase in leg press power at resistance of 70% body mass  Leg press strength increased 22% in PG and 12% in CG (p<0.001), but no group x time effect differences  No significant increases in functional outcomes for either PG or CG | Resistance training focusing on speed of movement improved leg power and maximal strength, but did not improve functional performance in healthy high-functioning older adults. |
| Kerschan et al.,1998 [70]  Austria  RCT | To investigate the functional impact of an unvarying long-term exercise program to be carried out at home | - n=124 - Sex: Female - Age: >60 y (mean 68.3 y) - Characteristics: Healthy non-disabled and sedentary | Follow-up 5-10 years (mean 7.7 years)  2 groups:  **Exercise (EG)**  Warm-up, stretching and strengthening exercise  Initially, 2 times/wk for >45 min  Followed by, 3 times/wk for 20 min  **Control (CG)**  Measurements:  Pain Disability Index (PDI)  Gait velocity  Strength  Postural sway  Number of fractures  t-test, chi-square, Kruskal-Wallis, logistic regression | Compliance of training group 36%  Gait velocity was slightly higher in EG than CG  No differences were found in PDI, muscle strength, body sway and fracture rate  The PDI was associated with-self chosen gait velocity | An unvarying home-based exercise program may support general agility but does not yield enough force to impact muscle strength and postural stability in healthy non-disabled postmenopausal women who start exercising at 60 y  This program did not yield a comprehensive improvement of functional outcome |
| Kolbe-Alexander and Charlton, 2006 [126]  South Africa  Non-RCT | To assess the effectiveness of a community-based, low-intensity exercise program | - n=91 - Sex: Female - Age: >60 y; mean 68 y - Characteristics: Sedentary elders from socio-economically and historically disadvantaged communities | Baseline, 10 and 20 wk  3 centres, 3 groups:  **Exercise (EX1, EX2)**  10 min warm-up, 35 min, seated low-intensity,10 min cool-down; 3 times/wk  **Control group (CTL)**  Met 2 times/wk for 20 wks for relaxation and art and crafts  Measurements:  ADL  Instrumental Activities of Daily Living (IADL)  Yale Physical Activity Survey (YPAS)  Blood pressure  6 min walk  Balance  Gait  Strength  ANOVA, ANCOVA, Chi-square | Exercise training improved dynamic balance in EX1 (19.8 s) and EX2 (16.3s )  Lower body strength improved in both exercise groups (sit to stand in 10 s)  BP in both exercise groups decreased from baseline to 20 wks  No effect on upper body strength, 2 m walk, 6 min walk, ADL, Health status | A community-based, low-intensity exercise program improved dynamic balance and lower body strength, but had no effect on walking measures or ADL |
| Miszko et al., 2003 [127]  USA  RCT | To determine whether power training was more effective than strength training for improving whole-body physical function and to examine the relationship between changes in anaerobic power and muscle strength and physical function | - n=39 (ST; n=17, PT; n=18, C; n=15) - Sex: Male and female - Age: Mean 72.5 y - Characteristics: Elders with below average leg extensor power (Males <210 W, females <140 W) | Pre and post,16 weeks at 3 times/wk  3 groups:  **Strength training (ST)**  3 sets of 6-8 reps of 8 exercises  5 min warm-up  weeks 1-8 50%-80% 1RM  weeks 9-16 80% 1RM  **Power training (PT)**  8 exercises  weeks 1-8 same as ST  weeks 9-16 3 sets of 6-8 reps at 40% of 1RM as fast as possible  **Control group (C)**  Maintained usual activity and attended 3 lectures  Measurements:  Continuous Scale Physical Functional Performance test (CSPFP)  Max strength (1RM)  Anaerobic power (Wingate)  ANCOVA, Pearson correlation coefficients | After controlling for base-line CSPFP was greater in PT vs. ST or C  (CI; PT=60.9-69.9, ST=54.5-62.8, C=54.1-61.8)  Anaerobic power was not correlated to change in CSPFP  No difference between groups for peak anaerobic power  Max strength was greater for ST vs. C post | Power training was more efficient than strength training in improving physical function |
| Nichols et al., 2005 [128]  USA  RCT | To examine the efficacy of a progressive resistance exercise program,  using equal concentric/eccentric or greater eccentric/concentric  workloads, for increasing strength and improving functional abilities of community-dwelling older adults. | - n=57 - Sex: Male and Female - Age: >60 y - Characteristics: Healthy, low activity status | Baseline and 14 weeks  3 Groups:  2 times/wk, upper and lower body  Equal concentric/eccentric (CE)  3 sets, 12 reps (4 sets for leg press)  Greater eccentric/concentric  (GE) workloads  3 sets, 10 reps, with negative phase weight increased  Control Group (CG)  Measurements:  1RM  Bag carry  Stair climb  Shelf Task 1RM  Static balance  Mixed model repeated-measures  ANOVA | No difference in strength gains between CE and GE.  Significant interaction occurred for the stair climb and balance with the exercise groups decreasing stair climb time by 11 % and increasing balance time by 26%. Relative improvements by weight trainers of 12**%** for the shelf task and 7**%** for the bag carry were not significant. | A resistance training program of moderate intensity twice a  week can increase muscular strength and improve selected functional abilities  of community-dwelling older adults |
| Ramsbottom et al., 2004 [129]  UK  RCT | To assess the effectiveness of a commu­nity-based exercise program on leg power, static balance, and functional mobility in healthy, normally sedentary people over the age of 70 years. | - n=16 (TR=10, CR=6) - Sex: Male and female - Age: >70 y - Characteristics: Healthy, sedentary | Baseline, 12, and 24 weeks  2 Groups: 2 times/wk  Training Group (TG): Progressive upper and lower body training using seated and non-seated exercises (body weight, dynabands, games)  Control Group (CG):  Measurements:  Leg power, static balance (postural sway), dynamic balance (functional reach), and functional mobility (6m timed walk)  Analysis:  2 Way Repeated ANOVA | TG, leg power increased 40%, from 108 to 141 W (*p* < .01); dynamic balance increased 48%, from 22.3 to 33.1 cm (*p* < .01; functional reach); and functional mobility increased 12%, from 7.46 to 6.54 (*p* < .05; timed walk)  CG showed no significant change | This community-based exercise program led to large improvements in leg-extensor power, dynamic balance, and functional mobility |
| Skelton and McLaughlin, 1996 [73]  UK  RCT | To determine the feasibility and acceptability of an exercise class and whether an 8 week period of moderate intensity exercise could improve strength, flexibility, balance and functional ability | - n=20 - Sex: Female - Age: >74 y (mean 81 y range 74-89 y) - Characteristics: Healthy elderly females | Pre and post, 8 weeks  3 groups:  **Training (1TG)**  1 class/wk and 2 home sessions (support book)  10 min warm-up and stretch, 30-40 min of strengthening (major muscle groups) 1-3 sets of 4-8 reps, 10 min warm-down  **Control (1CG)**  Maintained usual performance for 8 weeks then underwent training  **Training 2 (2TG)** as 1TG  Measurements:  Strength, flexibility, anthropometry, balance and functional ability, human activity profile (HAP), Philadelphia Geriatric Centre moral scale (PGCMS), The Geriatric Depression Scale (GDS)  ANOVA, t-test | Training improvements in strength, flexibility, balance and functional ability | Repeated moderate exercise which involves functional tasks and mobility can produce increases in strength, balance, flexibility and functional ability |
| Skelton 1995 [130]  UK  RCT | To determine if 12 weeks of progressive resistance strength training can produce an increase in strength and power in healthy women aged 75 and older and whether these increases improve functional ability | n=40  Sex: Female  Age: >75 y  TG: median 79.5 y (range 76-93 y)  CG: median 79.5 y (range 75-90 y)  Characteristics: Healthy | Pre and post, 12 weeks  2 groups:  **Training (TG)**  1 supervised class/week: 10min warm up and stretch; 30-40min upper and lower body progressive resistance exercises; 3 sets of 4-8 repetitions with body weight, rice bags, or Therbands; 10 minute warm down  2 home sessions/week with aid of booklet and audiocassette  Control Group (CG)  Asked to maintain current activity and complete exercise diary  Measurements:  Strength (knee extensor, elbow extensor, hand grip), leg extensor power, and functional ability (functional reach, chair rise, lifting bag onto a surface, box stepping, kneel rise, corridor walk, stair walking, floor rise, human activity profile (HAP)  Analysis:  Correlations, ANOVA, ANCOVA | No change in functional reach, stair climbing speed, walking rate, step rate, bag lifting, chair rising, or floor rising  Stepping up (p=0.005) and rising from the kneeling position on the floor (p=0.021) improved with strength training  Increased IKES (p=0.03), HGS (p=0.05), LEP (p=0.05) with training  *Control group had significantly greater body mass pre-testing  *Control group had significantly greater IKES and IEFS pre-training.  Not significant with weight as a covariate for all. | Isolated improvements of strength and power standardized for body weight may not be sufficient to improve functional ability in the study population |
| Taaffe 1999 [74]  USA  RCT | To examine the effect of frequency of progressive resistive training on muscle strength gain in healthy older adults in terms of absolute gains as well as the time course for improvement | n=46  Sex: Male (n=29), Female n=(17)  Age: 65 to 79 y  EXI (n=11, 68.5 y)  EX2 (n=12, 69.4 y)  EX3 (n=11, 71.0 y)  CO (n=12, 68.9 y)  Characteristics: Healthy | Pre , every 4 weeks, 24 weeks  3 groups:  Training groups:  High-intensity resistance training 1 time/week (EX1);  2 times/week (EX2);  or 3times/week (EX3)  Warm up with stretching and one set of bench press and leg press (40%1RM); 3 sets of 8 exercises at 80%1RM (bench press, military press, latissimus pull down, biceps curl, leg press, knee extension/flexion, back extension  Control (CO)  **Measurements**:  Upper and lower body muscle strength, 1RM  Chair rise test  6m backward walk  **Analysis:**  ANOVA, ANCOVA, t-tests, Pearson correlation | For each of the eight exercises, muscle strength increased in the exercise groups relative to control (p<0.01), with no difference among EX1, EX2, and EX3 groups at any measurement interval  Time to rise successfully from a chair 5 times decreased significantly (p<0.01) and was correlated to changes in quadriceps strength (r=-0.40, p<0.01) and lean mass (r=-0.40, p<0.01)  6m backwards walk time was reduced but not significant (p=0.10). | A program of one or twice weekly resistance exercise achieves muscle strength gains similar to 3 days per week and is associated with improved neuromuscular performance |
| Vincent 1995 [75]  USA  RCT | To examine the strength and physical function responses to 6 months of high- or low-intensity resistance training in older adults | n=62  Age: 60 to 83 y  Sex: Male and Female  CON: n=16, mean age 71.0 y  LEX: n=24, mean age 67.6 y  HEX: n=22, mean age 66.6 y  Characteristics: Healthy, not engaged in regular resistance training | Baseline, 6 months  3 Groups:  Low-intensity training (LEX)  High-intensity training (HEX)  Control Group (CON)  3 times/week  Abdominal crunches, leg press, leg extension, leg curl, calf press, seated row, chest press, overhead press, biceps curl, seated dip, leg abduction and adduction, lumbar extensions (1 time/wk)  LEX: 1 set, 8 reps, 50%1RM  HEX: 1 set, 13 reps, 80%1RM  Measurements:  Muscular strength: 1RM for leg press and curl, knee extension, chest and overhead press, seated row, triceps dip, and biceps curl  Isometric lumbar extension force curve  Muscular endurance: max reps with 60% 1RM  Stair climbing time  Analysis:  3x2 repeated measures ANOVA; Scheffe post hoc; ANCOVA | 1RM in all test exercises increased significantly in both LEX and HEX (p<0.05) with no difference between groups  Muscle endurance increased significantly for leg press and chest press in both LEX and HEX (p<0.05)  Stair climbing time decreased significantly for both LEX and HEX (p<0.05); change associated with leg press, leg curl, and leg extension but not with endurance | High and low resistance exercise yielded significant and similar improvements in muscular strength and endurance and stair climbing ability |

ADL – Activities of Daily Living; ANOVA (ANCOVA) – Analysis of variance (covariance); m - metres; MANOVA - Multivariate analysis of variance; MET – Resting metabolic equivalent; reps - repetitions; RM - Repetition maximum; SPPB - Short physical performance battery; VO_2peak_ - Peak oxygen uptake; W - Watt
