# Supplementary material for: Physical activity and functional limitations in older adults: a systematic review related to Canada's Physical Activity Guidelines
Source: Int J Behav Nutr Phys Act. 2010 May 11;7:38. doi: 10.1186/1479-5868-7-38 (PMC2882898; doi:10.1186/1479-5868-7-38)
Supplement: Additional file 7 — Supplemental table 9. Table s9: Strength studies assessed with the modified Downs and Black Quality Assessment Tool. [file 1479-5868-7-38-S7.DOC]

| **Publication**  **Country** | **Reporting**  **(11)** | **External Validity**  **(2)** | **Internal Validity**  **-Bias (6)** | **Internal Validity –Confounding (5)** | **TOTAL (24)** |
| --- | --- | --- | --- | --- | --- |
| Ades et al., 1996 | 7 | 0 | 5 | 5 | 17 |
| Bean et al., 2004 | 9 | 0 | 4 | 4 | 17 |
| Buchner ,1997  See Table 7. | - | - | - | - | - |
| Brandon, 2000 | 7 | 0 | 5 | 5 | 17 |
| Earles, 2001 | 9 | 0 | 5 | 5 | 19 |
| Kerschan et al., 1998 | 7 | 0 | 4 | 5 | 16 |
| Miszko et al., 2003 | 8 | 0 | 5 | 4 | 17 |
| Nichols et al., 2005 | 8 | 0 | 5 | 4 | 15 |
| Ramsbottom et al., 2004 | 9 | 0 | 5 | 5 | 20 |
| Skelton and McLaughlin, 1996 | 9 | 0 | 5 | 5 | 19 |
| Skelton, 1995 | 8 | 0 | 5 | 5 | 18 |
| Taaffe, 1999 | 8 | 0 | 5 | 5 | 18 |
| Vincent, 1995 | 8 | 0 | 5 | 5 | 18 |

| **Publication**  **Country** | **Reporting**  **(11)** | **External Validity**  **(2)** | **Internal Validity**  **-Bias (5)** | **Internal Validity**  **–Confounding (5)** | **TOTAL (23)** |
| --- | --- | --- | --- | --- | --- |
| Ashmead and Bocksnick, 2002 | 6 | 0 | 5 | 3 | 14 |
| Bunout et al., 2001 | 9 | 0 | 5 | 5 | 19 |
| Capodaglio and Capodaglio-Edda ,2007 | 8 | 0 | 5 | 2 | 15 |
| Cavani et al., 2002 | 8 | 0 | 5 | 2 | 15 |
| Kolbe-Alexander and Charlton, 2006 | 10 | 0 | 5 | 4 | 19 |
