# Supplementary material for: Physical activity and functional limitations in older adults: a systematic review related to Canada's Physical Activity Guidelines
Source: Int J Behav Nutr Phys Act. 2010 May 11;7:38. doi: 10.1186/1479-5868-7-38 (PMC2882898; doi:10.1186/1479-5868-7-38)
Supplement: Additional file 8 — Supplemental table 10. Table s10: Studies examining the relationship between physical activity and cognitive function in older adults [131-148]. [file 1479-5868-7-38-S8.DOC]

| **Publication**  **Country**  **Study Design** | **Objective** | **Population** | **Methods** | **Outcome** | **Comments and Conclusions** |
| --- | --- | --- | --- | --- | --- |
| Abbott et al., 2004 [83]  United States  Prospective cohort | To examine the association between walking and future risk of dementia in older men | - N = 2257 - Sex: Male - Age: 71-93 y - Characteristics: enrolled in the Honolulu-Asia Aging Study (Japanese ancestry living in Hawaii); healthy, no cognitive impairment, non-smoking, physically capable of walking, not working | 3 y follow-up  Data were collected at baseline (1991-1993) and follow up (1994-1996).  PA was reported as the average amount of distance walked per day  CASI for initial screening of dementia. Those identified by the CASI questionnaire underwent a full examination and diagnosis was made by a consensus panel (based on the Diagnostic and Statistical Manual of Mental Disorders, Revised 3^rd^ Edition).  ANCOVA, logistic regression and proportional hazards regression models | Age adjusted Incidence (per 1000 person-years):  Walking <0.25 miles/day: 17.8  Walking 0.25-1 miles/day: 17.6  Walking >1 -2 miles/day: 14.1  Walking > 2 miles/day: 10.3  Incidence was significantly higher in the <0.25 miles/day group and in the 0.25-1 miles/day group.  Relative Hazard (vs. >2 miles/day group; 95% CI):  Walking <0.25 miles/day: 1.77 (1.04-3.01)  Walking 0.25-1miles/day: 1.71 (1.02-2.86) | Physically capable elderly men who walk more are less likely to develop dementia. |
| Aley et al., 2007 [131]  United States  Cross-sectional | To investigate the effects of regular exercise on reaction times of elderly women during ambulation tasks | - n=31 (15 exercisers, 16 non-exercisers) - Sex: Female - Age: 65-82 y - Characteristics: no orthopaedic, neurological, cardiac or hearing impairments | Reaction time was measured using a portable computer and radio system; with 30 trials of 3 conditions (sitting, walking on tile and walking on carpet).  Exercise habits determined via questionnaire.  Mixed design ANOVA with paired t-tests | There was a significant effect of condition (sitting, walking on tile, walking on carpet) on reaction time  Exercisers and non-exercisers had similar reaction times. | Women who exercise regularly did not appear to have faster reaction times than non-exercising women |
| Ari et al., 2004 [132]  Turkey  Cross-sectional | To investigate the effect of regular exercise on maximum oxygen uptake capacity, reaction time, testosterone, growth hormone and insulin-like growth factor in athletes compared to sedentary control | - n=21 (10 athletes, 11 sedentary controls) - Sex: Male - Age: 68 y (athletes); 65 y (sedentary controls) - Characteristics: Athlete group were masters athletes exercising regularly | Physical fitness was assessed via Astrand cycle ergometer test.  Reaction time was assessed via general vocational aptitude test  Between-group differences evaluated by Mann-Whitney U test. Spearman’s rho correlation analysis for relationships | Physical fitness (ml/min/kg)  Athletes: 31.2 ± 6.2  Control: 18.8 ± 5.1  Reaction time (sec)  Athletes: 106.7 ± 23.2  Control: 148.3 ± 39.3  Fitness was significantly higher and reaction times were significantly faster in the athletes vs. controls  Spearman’s rho correlation: (Reaction time and physical fitness)  Athletes: 0.394 (not significant)  Controls:-0.141 (not significant) | Long term exercise is associated with decreased event-related reaction time |
| Barnes et al., 2003 [88]  United States  Prospective cohort | To determine whether cardiorespiratory fitness at baseline is associated with maintenance of cognitive function over 6 years in healthy older people | - n=349 - Sex: Male and female - Age: 59-88 y (mean 69 y) - Ethnicity: 97% White - Characteristics: Were participating in an ongoing longitudinal study, no evidence of cognitive impairment at baseline | Cardiorespiratory fitness via standardized treadmill protocol.  Cognitive function with mMMSE (3 items removed)  Unadjusted and adjusted linear regression to examine change in mMMSE score ANOVA adjusted for sex to examine effect of baseline physical fitness tertile | Change in mMMSE from baseline to year 6 (by sex-specific tertile of peak VO_2_ (95% CI):  Lowest tertile: -0.5 (-0.8 to 0.3)  Middle tertile: -0.2 (-0.5 to 0.0)  Highest tertile: 0.0 (-0.3 to 0.2)  Trend was significant | Baseline measures of cardiorespiratory fitness were positively associated with preservation of cognitive function over a 6 year period |
| Benloucif et al., 2004 [133]  United States  Crossover  design | To determine whether a single daily session of social and moderate physical activity would be sufficient to improve sleep and day-time neuropsychological performance in older adults and to access whether the time of participation would affect the intervention | - n=14 (2 dropouts) - Sex: Male and female - Age: 67-86 y (mean 74.6 y) - Characteristics: living in independent-living retirement facilities, no or mild dementia, healthy or with stable chronic conditions, independent in ADL, no psychotropic or hypnotic medication use | Counterbalanced crossover study design with exercise in either the morning or evening with a 1 month washout period. Testing before and after each phase of the intervention.  Intervention was administered in the living facility in small groups. Sessions were 1.5 hr and included 30 min mild exercise, 30 min social interaction and 30 min of mild to moderate exercise  Physical activity via wrist-worn activity monitors  Cognitive function was with paper and pencil tests and the Automated Neuropsychological Assessment Metrics test battery.  rmANOVA for treatment effects | Morning activity:  4/8 performance tasks significantly improved (mathematical processing, digit symbol, visual search, 2-letter logical reasoning test) with effect sizes of 0.858, 0.801, 1.235 and 0.720 respectively  Evening activity  7/8 performance tasks improved (Sternberg memory of 4 letters, mathematical processing, running memory, symbol copy, digit symbol, visual search, 2-letter logical reasoning test) with effect sizes of 0.753, 0.719, 0.934, 0.817, 1.075, 0.982, and 0.598 respectively. | The 14 day social/physical activity intervention improved neuropsychological performance |
| Bixby et al., 2007 [134]  United States  Cross-sectional | To determine whether physical activity participation was specifically and positively associated with executive function in older individuals after accounting for age, education and IQ | - n=122 (2 outliers) - Sex: Male and female - Age: 65-92 y (mean 78.9 y) - Characteristics: no depression, psychotropic medication, stable physical activity patterns for 3-5 years | Physical activity was assessed using the YPAS  Executive function was assessed using the Stroop colour and word test (yields 4 scores: Stroop word, Stroop colour, Stroop colour and word and Stroop interference)  Hierarchical regression (accounting for age and IQ) | YPAS score did not explain a significant amount of the variance in Stroop word or Stroop colour scores  YPAS score explained a significant amount of the variance in Stroop colour and word score (∆R^2^ = 0.02) and in Stroop interference (∆R^2^ = 0.04). | Chronic physical activity explained a small but significant amount of variance in Stroop executive task performance (beyond age and cognitive stimulation) across the age range |
| Blumenthal et al., 1989 [90]  United States  RCT | To assess the cardiovascular, psychological and behavioural effects of aerobic exercise training in a group of healthy older men and women | - n=101 (33 aerobic, 34 yoga, 34 control); 4 dropouts by 16 weeks (4%) - Sex: Male and female - Age: >60 y (range 60-83 y; mean = 67 y) - Ethnicity: 96% white - Characteristics: Healthy | Aerobic exercise group: 30 min cycling 3 times/wk for 16 weeks at 70% HRR.; followed by 15 min of walking, jogging and arm ergometry;. ten min warm up and 5 min cool down  Yoga group:  Yoga exercises at least 2 times/wk for 16 weeks for 60 min per session (non-aerobic)  Wait list:  Instructed not to change PA habits.  Memory was assessed using the Digit Span Subtest of the WAIS-R, Benton Visual Retention Test, the Randt Short Story Subtest and the Selective Reminding Test  Other tests included the Stroop Test and the Nonverbal Fluency and Verbal Fluency tests.  MANOVA with univariate ANOVA when significant multivariate effects were observed | A significant effect of sex and/or time emerged in many of the measures but there was no effect of group (aerobic exercise vs. yoga vs. wait list). | Improved performance on tests was not unique to a particular group and was attributed to practice and familiarity |
| Broe et al .,1998 [80]  Australia  Prospective cohort | To examine the effects of exercise, smoking and alcohol use on cognitive test performance, dementia and Alzheimer’s disease | - n=327 - Sex: Male and female - Age: 78-99 y (mean 83.4 y) - Characteristics: involved in the Sydney Older Persons Study, non-demented at baseline | Physical activity (gardening, active sports and walking) assessed at baseline via questionnaire (reported in times/month).  Cognitive function assessed 3 years later at follow up (Logical Memory I and II, Visual Reproduction I and II, Verbal Fluency-FAS, Verbal Fluency-Animals, National Adult Reading Test, Cube Copying, Clock Drawing, MMSE)  Partial correlations (controlling for age, sex and education) for relationship between health habits and tests | No significant correlations between gardening and cognitive test performance  No significant correlations between active sports and cognitive test performance  One significant correlation (negative) between walks and cognitive test performance:  Cube Copying: -0.17 | There was little association between physical activity and cognitive test performance |
| Bryan and Ward, 2002 [135]  Australia  Prospective cohort | To examine cross-sectional and longitudinal effects of history of smoking, alcohol use and engagement in exercise on cognitive performance | - n=945 - Sex: Male and female - Age: 65-98 y (mean 76.9 y) - Characteristics: Participant in the Australian Longitudinal Study of Ageing, data at baseline and follow-up | Exercise assessed at baseline and follow-up via 3 questions about activity during the previous 2 weeks  Cognitive performance and memory were assessed at baseline and follow up using BNT, DSST, MMSE.  Pearson correlation for relationship between exercise and cognitive performance variables. RM ANOVA ( RM - repeated measures) between baseline and follow- up with age, schooling, self-rated health, number of medical conditions, number of medications and NART errors as covariates | Exercise was significantly correlated with:  Immediate picture recall at follow up: 0.07  DSST at baseline and follow up: r = 0.14 at both time points  Exercise was not significantly correlated with any other cognitive performance measures  There was no association (using RM ANOVA) between exercise and cognitive function after controlling for covariates. | After controlling for covariates, exercise was not related to cognitive performance |
| Caplan et al., 1993 [91]  Australia  Non-randomized intervention | To assess the effects of a twice-weekly aerobic weight-bearing exercise program on bone density, risk factors for cardiovascular disease, wellbeing and cognitive function | - n=30 (19 exercisers, 11 controls) - Sex: Female - Age: Mean 66.4 y - Characteristics: Postmenopausal, had been referred for bone densitometry and solicited to join the Fitness for Seniors program, not using estrogens, progestogens, vitamin D, anabolic steroids, bisphophonates or fluoride, no diseases that aggravate osteoporosis | Physical activity group:  60 min 2 times/wk for 40 wk each year (2 years total; 4 terms of 10 wk each y). Sessions included warm up, 20-25 min low impact aerobic exercise, 10 min of ball games, strength and flexibility exercises, 10 min relaxation; instructed to complete 1 session/wk where heart rate was elevated for 20-30 min  Control group:  Consisted of individuals from the Fitness for Seniors program who did not want to participate in the exercise group. Were given no instructions about exercise  Cognitive function was assessed using the MMSE.  Unpaired t-tests for differences between groups | Exercise group:  MMSE at end: 28.6 ± 0.5  Control group:  MMSE at end: 28.5 ± 0.3    Groups were not significantly different | There was no difference between the exercise and the control group in cognitive function after 2 years |
| Cassidy et al., 2004 [136]  Australia  Cross-sectional | To investigate the association between potentially modifiable lifestyle factors and cognitive abilities/depressive symptoms in community-dwelling women aged 70 plus | - n=278 - Sex: Female - Age: >70 y (mean=74.6 y) - Characteristics: Community-dwelling postmenopausal women | Questionnaire assessing current levels of Physical activity; participants were then classified as physically inactive or active (30 min exercise on most days of the week).  CAMCOG instrument to assess cognitive function.  Mann-Whitney *U*-tests for ordinal data and Student’s t-tests for parametric variables | Cognitive Function (Physically Inactive)  CAMCOG Score: 94.6 ± 5.3  Cognitive Function (Physically Active)  CAMCOG Score: 94.8 ± 4.4  There was no difference is CAMCOG scores between the groups | There was no association between physical activity and cognition |
| Cassilhas et al., 2007 [89]  Brazil  RCT | To assess the impact of 24 weeks of resistance training at two different intensities on cognitive functions in the elderly | - n=62 (23 control, 19 moderate exercise, 20 high exercise) - Sex: Male - Age: 65-75 y - Characteristics: healthy, sedentary, more than 8 y of schooling, no dementia | Moderate and high intensity groups: Resistance training 3 times/wk, 1 hr/session for 24 weeks; cycling 5-10 min for warm-up with stretches  **Moderate intensity**  50% 1RM  **High intensity**  80% 1RM  **Control group**  Warm-up and stretching 1 time/wk  WAIS-III (digit span forward and backward), WSM-R (Corsi’s block tapping forward, backward, similarities), Toulouse-Pieron’s concentration attention test (cancellations numbers, errors), Rey-Osterrieth complex figure (copy, immediate recall) to evaluate cognitive function before and after the intervention.  ANCOVA with pre-intervention value using Duncan’s post hoc test where necessary | After the intervention both exercise groups showed significant changes from the control group in:  WAIS-III (Digit span forward)  WSM-R (Corsis block tapping backward, Corsis block tapping similarities)  Rey Osterrieth figure (immediate recall)  After the intervention there was no difference between the groups for:  WAIS-III (Digit span backward)  WSM-R (Corsis block tapping backward)  Toulouse-Pieron (cancellations numbers)  Rey Osterrieth figure (copy)  After the intervention the high exercise group was significantly different from the control group for:  Toulouse-Pieron (errors)  There were no differences between the moderate and high exercise groups after the intervention | The moderate and high-intensity resistance training programs had equally beneficial effects on cognitive functioning |
| Deary et al., 2006 [137]  Scotland  Prospective Cohort | To test the hypothesis that physical fitness is associated with more successful cognitive aging | - n=460 - Sex: Male and female - Age: 79 y - Characteristics: Surviving participants of the Scottish Mental Survey of 1932 (born in 1921), healthy, no cognitive impairment | General Mental ability assessed with the MHT at age 11 y and age 79 y.  Physical fitness assessed with a 6-meter walk test and by measuring grip strength.  Fitness data were adjusted for sex and height. Principal components analysis (for 6 meter walk time, grip strength and FEV_1_) was used to create a summary “fitness” variable. Pearson correlations and multivariable linear regression for contributions | MHT IQ at age 79 y was correlated to the summary fitness variable at age 79 y (0.22)  In the multivariable linear regression fitness accounted for 3.3% of the variance in MHT IQ at age 79 y | Physical fitness is associated with cognitive reserve |
| Fabre et al., 2002 [96]  France  RCT | To compare the effects of aerobic and mental training on cognitive function and to determine if the association of the two techniques shows better results | - n=32 - Sex: Male and female - Age: 60-76 y - Characteristics: Recruited from clubs, normal ECG during exercise, not depressed, able to breathe through mouth during exercise, not engaged in regular physical activity | Random assignment to  4 groups: aerobic training only, mental training only, combined aerobic and mental training, control.  Aerobic training:  2times/wk, 1 hr/session for 2 months (walking/jogging intervals at a heart rate equivalent to ventilatory threshold)  Mental training:  1 time/wk, 90 min/session for 2 months (8 themes: perceptive activities, attention, intellectual structuration, association and imagination, language, spatial marks, temporal marks, associated recruiting).  Control group:  Met the same number of times as the other groups for leisure activities such as painting and choral singing.  BEC questionnaire for cognitive problems and Wechsler memory scale administered before and after the intervention  One-way ANOVA to compare descriptive values between groups. Two way RM -ANOVA for effect of training with pair-wise Newman-Keuls tests when F ratio was significant | BEC questionnaire:  No change in BEC scores in any group after the intervention  Wechsler memory scale -  Memory quotient: All 3 training groups improved; combined group increased more than aerobic or mental training only; no change in control group  Paired associated learning: All 3 training groups improved; no change in control group.  Logical memory immediate recall: All 3 training groups improved; no change in control group.  No significant change in any group for digit span forward, orientation, general information, mental control or visual reproduction portions of the test | The aerobic training program led to an improvement in cognitive function. Combined mental and aerobic training led to greater improvements. |
| Hagen et al., 2003 [138]  United Kingdom  Non-randomized intervention | To examine the physical, emotional, behavioural and cognitive effects of a 10-week program of musical exercise therapy immediately after and 10 weeks after a 10 week intervention | - n=60 (20 exercise therapy, 20 occupational therapy, 20 control) - Sex: Male and female - Age: Mean 78.3 y - Characteristics: Recruited from a long term care facility, medically stable, no cognitive impairment | **Exercise therapy group**  3 times/wk, 40 min/session for 10 weeks (stretching, flexibility, mild aerobic).  **Occupational therapy group**  3 times/wk, 1 hr/session for 10 weeks (crafts, social activities, ADL)  **Control group**  Routine nursing care  CAS for mental functioning before, immediately after and 10 weeks after the end of the intervention  ANOVA for effect of intervention | CAS Scores:  Exercise therapy: Scores were increased at the end of the 10 weeks and declined slightly by 10 weeks post-intervention  Occupational therapy: Scores were increased at the end of the 10 weeks and remained elevated 10 weeks post-intervention  Control: Scores decreased over the course of the study  There was a significant interaction between group and time of measurement | Exercise therapy was associated with an increase in cognitive function at the end of the 10 week intervention |
| Hassmen et al., 1992 [139]  Sweden  Non-randomized intervention | To study whether the physical activity intervention results in improved cognitive performance after 3 months. | - n=32 (2 dropouts) - Sex: Female - Age: 66-75 y (there was also a younger age group) - Characteristics: Healthy | **Exercise group**  2.4 km walking at specified RPE level 4 times in between pre and post testing. Walking, at least 3 times/wk for 20 min outside of lab sessions  **Non-exercise group**  Training in intellectual functions (mental math, problem solving, logical thinking tasks) for same amount of time as exercise group.  Face recognition task, simple reaction time, complex reaction time and digit span tests for cognitive performance  2 x 2 mixed RM-ANOVA for the effect of training | Face recognition task:  No significant difference between groups or after intervention  Simple reaction time:  No significant difference between groups or after intervention  Complex reaction time:  Both groups decreased complex reaction time at the end of the study; exercise group decreased time more than the non-exercise group  Digit span:  Digit span increased in the exercise group after the intervention; no change in the non-exercise group. | The physical activity intervention was associated with an improvement in digit span test scores |
| Hatta et al ., 2005 [140]  Japan  Cross-sectional | To examine the effects of habitual moderate exercise on response processing and cognitive processing in older participants | - N=40 (20 active, 20 inactive) - Sex: Male and female - Age: Mean 69.2 y and 66.9 y for active and inactive respectively - Characteristics: Healthy, right handed, free of neurological and psychiatric disorders | Active group consisted of individuals who had been taking part in a 90 min session 1 time/week for more than 3 years as well as walking at least 60 min almost every day  Inactive group had no history of exercise  Reaction time to evaluate response processing. EEG recordings (P3 latency and amplitude) for cognitive processing.  ANOVA with Fisher PLSD post hoc test for reaction time. RM-ANOVA (with Greenhouse and Geisser adjustment) for EEG recordings | Reaction time (ms):  Active: 379.98 ± 14.3  Inactive: 432.49 ± 15.9  P3 latency (ms):  Active: 372.57 ± 5.4  Inactive: 364.97 ± 4.7  P3 amplitude (µV):  Active: 11.11 ± 0.6  Inactive  Active group had significantly faster reaction time and higher P3 amplitude. | Habitual physical activity appears to have a positive influence on response and cognitive processing |
| Hill et al., 1993 [92]  United States  Non-randomized intervention | To examine the relationship between indices of fitness and cognitive function and to evaluate the effect of a 1 year exercise training program on cognitive measures | - N=229 (108 dropouts, 47%) - Sex: Male and female - Age: 60-73 y - Ethnicity: Caucasian - Characteristics: Healthy, non smokers, normally physically active with no exercise training for at least 2 years | Exercise group:  Individualized training based on VO_2max_ results. Flexibility training for 2 months. Progressive aerobic exercise training (3-5 times/wk, 50 min/session for 9-12 months) with intensity monitored by heart rate  WAIS-R digit symbol subtest, WMS logical memory subtest and crossing-off task measures before and after the intervention  Analysis of partial variance and MANOVA to assess between group differences with univariate ANOVAs to locate differences | WAIS-R digit symbol:  Exercise pre: 55.42 ± 11.58  Exercise post: 57.60 ± 11.51  Control pre: 55.32 ± 9.84  Control post: 56.03 ± 10.83  WMS Logical memory:  Exercise pre: 11.51 ± 3.12  Exercise post: 11.57 ± 3.04  Control pre: 11.08 ± 2.98  Control post: 9.41 ± 2.56  Crossing-off task:  Exercise pre: 184.89 ± 33.00  Exercise post: 186.58 ± 31.36  Control pre: 183.00 ± 29.12  Control post: 181.60 ± 28.51  Significant effect of group was caused by a decrease in WMS logical memory score in the control group at the post session | The long term exercise training program had little effect on improving cognitive function |
| Hillman et al ., 2002 [141]  United States  Cros- sectional | To determine the influence of cardiovascular fitness on cognitive and motor processes. | - n=48 - Sex: Male and female - Age: 18-28 y and 60-70 y (only older group presented here) - Characteristics: healthy, no medications that influence CNS function, normal (or corrected to normal) vision | Participants belonged to 4 groups (older aerobically trained, older sedentary, younger aerobically trained, younger sedentary).  Physical fitness from graded exercise test for VO_2max_. Physical activity from YPAS.  Cognitive processing accessed via reaction time task (easy and hard conditions). Event related potential data also collected (not presented here).  Mixed model multivariate tests with repeated measures. Univariate ANOVAs and paired t tests for follow up where needed | Reaction time latency (ms)  Older fit (easy): 338.1 ± 97.2  Older fit (hard): 335.1 ± 66.9  Older sedentary (easy): 352.3 ± 149.3  Older sedentary (hard): 354.4 ± 162.3 | Reaction time did not differ by fitness group. |
| Hillman et al 2006 [142]  United States  Cross sectional | To examine the relationship of physical activity to cognition with a task requiring variable amounts of executive control | - N=241 - Sex: Men and women - Age: 15-71 y - Characteristics: Community dwelling individuals | Measurements:  WAIS-III  Eriksen Flanker Task  Physical activity questionnaire (days per week)  Regression analysis | Reaction time during congruent trials and PA: (partial correlation) = -.14 t(235) = 2.4, *p*<0.02, β = -.14  Reaction time during incongruent trials and PA: pr = -.17 t(235) = 2.8, *p*=0.005, β = -.17  Response accuracy during incongruent trials and PA: pr = -.20 t(235) = 3.2, *p*=0.002, β = -.18  Results indicate that greater PA participation was associated with faster reaction time and greater task performance | Physical activity may be beneficial to both general and selective aspects of cognition, particularly among older adults |
| Inzitari et al 2007 [143]  United States  Prospective | To examine if gait speed predicts decline in attention and psychomotor speed in older adults | - N=2,776 - Sex: Men and women - Age: 73.5 ± 2.8 y - Characteristics: Older community dwellers; The Health Aging and Body Composition Study | Measurements:  Psychomotor speed:  DSST  Physical performance:  Gait speed (m/s) over a 6m course    Quartiles:  Q1 = <1.05 m/s  Q2 = 1.06-1.19 m/s  Q3 = 1.20-1.34 m/s  Q4 = >1.35 m/s  3MMNSE | Adjusted odds ratio for risk of decline in DSST over 5 years across quartiles of usual gait speed (m/s):  Q1 = 1.849  Q2 = 1.413  Q3 = 1.180  Q4 = 1 (referent) | In this study of older community dwellers, gait speed independently predicted a decline in DSST after 5 years |
| Kalapotharakos et al., 2006 [115]  Greece  RCT | To determine the effects of a 12 week aerobic exercise program on functional and neuromotor performance in inactive healthy older adults | - N=23 (12 exercise, 10 control); 1 dropout - Sex: Men and women - Age: 60-75 y - Characteristics: Inactive, no cognitive impairment, no depression, healthy. | **Exercise Group**  Treadmill walking 3 times/wk, for 12 wk on non-consecutive days. Sessions progressively increased duration and intensity of exercise  **Control Group**  Did not exercise  Neuromotor performance from whole body reaction time test  MANOVA for differences in main effects and time by group interaction | Whole body reaction time (seconds)  Aerobic pre: 1.075 ± 0.286  Aerobic post: 0.877 ± 0.204  Control pre: 1.065 ± 0.15  Control post: 1062 ± 0.169 | Whole body reaction time decreased significantly in the exercise group after 12 weeks of training |
| Landi et al., 2007 [87]  Italy  Prospective cohort | To evaluate the relationship of lifetime physical activity with cognitive performance  Data from the ilSIRENTE Study | - N=363 - Sex: Male and female (67%) - Age: Mean 85.9 y - Characteristics: Community dwelling | Baseline 2003-2004  Measurements:  -Questionnaire on physical activity frequency of light and high PA (4 point scale) during 3 different periods:  20-40 y  41-60 y  Year before assessment  -Light Activity i.e. walking  fishing, dancing <3 times/wk  -Moderate activity i.e. swimming, jogging 1 or 2 times/wk or light activity >4 time/wk  -Moderate activity >3 times/wk  - High activity i.e. sports, aerobic running, cycling >2 times/wk  -Cognitive Performance Scale (CPS) 6 item, 7-category scale  Impairment score >1  ANCOVA, regression | Adjusted for age, gender and depression the risk of cognitive impairment is:  20-40 yrs  High intensity OR 0.49 (95% CI 0.28-0.86)  40-60 yrs  High intensity OR 0.50 (95% CI  0.28-0.91)  Year before assessment  High intensity OR 0.10 (95% CI  0.02-0.34)  Using low-moderate activity as a referent | The study suggests a history of high physical activity is associated with better cognitive performance |
| Larson et al., 2006 [144]  USA  Longitudinal | To determine whether regular exercise is associated with a reduced risk for dementia and Alzheimer disease  The Adult Changes in Thought (ACT) study | - N=1,740 - Sex: Male and female - Age: >65 y in 1994-1996 - Characteristics: Without cognitive impairment who scored >25^th^ percentile on the CASI | Baseline and a mean of 6.2 ± 2.0 follow-up  Measurements:  -Self reported exercise frequency  Questionnaire, number of days/wk for >15 min of the following: walking, biking, hiking, aerobics or callisthenics, swimming, water aerobics, weight training or stretching, or other exercise during the previous year  -Cognitive function, CASI  Cox proportional hazard | 158 developed dementia (107 Alzheimer’s)  Incidence rate of dementia 13.0 per/1000 person-year for exercisers >3 times/wk vs. 19.7 per/1000 person-year for exercisers <3 times/wk  The age and sex adjusted HR of dementia for regular exercise 0.62 (95% CI, 0.44-0.86; *p*=0.004) | The results suggest that regular exercise is associated with a delay in the onset of dementia and Alzheimer’s disease |
| Lindsay et al., 2002 [82]  Canada  Prospective  CSHA-2 case-control | To analyse the risk factors for Alzheimer’s disease  The Canadian Study of Health and Aging (CSHA) phase 1, and 5 years later phase 2 | - N=4,615 - Sex: Male and female - Age: >65 y - Characteristics: Cognitively normal at baseline | Baseline (CSHA-1), 5 year follow-up (CSHA-2)  Measurements:  **CSHA-1 (1991-1992)**  -ADL  -Modified Mini-Mental State Examination (3MS)  Criteria for dementia, the National Institute of Neurological and Communicative Disorders and Stroke-Alzheimer’s Disease and Related Disorders Association (NINCDS-ADRDA)  -Question on participation in regular exercise yes/no  **CSHA-2 (1996-1997)**  -As CSHA-1 plus:  National Institute of Neurological Disorders and Stroke (NINDS-AIREN)  t-test, chi-square, logistic regression | n=194 case, n=3,894 controls  After adjusting for age, sex and education regular physical activity was associated with a lower risk of Alzheimer’s disease OR 0.69 (95% CI, 0.50-0.96) | Regular physical activity could be an important component of a preventive strategy against Alzheimer’s disease and many other conditions |
| Newson and Kemps, 2006 [145]  Australia  Cross-sectional | To examine whether cardio-respiratory fitness influences cognitive aging and whether this influence is domain specific | - N= 25 young old, 25 middle-aged, 25 old-old - Sex: Male and female - Age: 65-74 y young old, 75-84 y middle-aged, 85-92 y old-old - Characteristics: Community dwelling adults with no neuropsychological disorders | 3 groups:  Young old  Middle-aged  Old-old  Measurements:  -Estimate model of VO_2max_  (Jackson et al .,1990) formula from age, BMI, gender ranking of physical activity level  -BMI  -Physical Activity Rating questionnaire  **-Attention**  Stroop task and Map Search Test  **-Working memory**  WAIS-III and Corsi Blocks Backward task  **-Speed of processing** DSST, Boxes Test  **-Executive function**  Zoo Map Test, Six Element Test  **-Memory**  The People Test and Name Test  **-Biological aging**  Auditory acuity, grip strength, systolic BP, FEV_1_  ANOVA, regression | Cardio-respiratory fitness accounted for:  53% of total variance in attention and 84% of the age-related variance  21% of total variance in working memory and 61% of the age related variance  36% of total variance in speed of processing and 67% of the age related variance  14% of total variance in executive function and 51% of age related variance  13% of total variance in memory and 59% of age related variance | The results suggest that cardio-respiratory fitness may have a selective protective effect against age-associated cognitive decline |
| Ojofeitimi et al., 2002 [146]  Nigeria | To examine the relationship between nutritional status, physical activity levels, and cognitive performance | - N=84 - Sex: Male and female (77%) - Age: 55-94 y - Characteristics: Healthy, non-institutionalized elders | Measurements:  Structured interview  -MMSE modified  -Nutritional assessment  -Physical activity assessment questionnaire/scale, frequency and duration responses were weighted from 0-19 points  Divided into:  **Very active**, >14 points  **Moderately active**, 8-13 points  **Non active**, <8 points  Chi-square, correlation | Cognitive score decreased with age (r=.037, *p*=0.03)  A relationship with cognitive function and physical activity levels (r=0.44, *p*=0.000)    Participants with better cognitive function score (>20) were very active (69%) or moderately active (31%)  75% of participants with low cognitive function score (<17) were inactive | Based on the findings regular intake of supplements and moderate physical activity will not only produce a healthier elderly population, but will also improve their cognitive function |
| Oken et al., 2006 [93]  USA  RCT | To determine the effects of yoga on cognitive function | - N=135 - Sex: Male and female - Age: 65-85 y - Characteristics: Healthy | Pre and post 6 months  3 groups:  **Hatha yoga class**  1 class/wk for 90 min Home practice  Total 18 poses with average 7-8 poses /class  **Walking exercise**  1 class/wk of 1 hour  Warm-up, exercise at 70% HR_max_ and 6-7 on Borg scale  Home exercise encouraged >5 times/wk  **Wait list control**  No intervention  Measurements:  Stroop colour and word Test  Electroencephalogram (EEG)  SF-36  Profile of Mood States (POM)  Multi-Dimensional Fatigue Inventory  WAIS-III  Stanford Sleepiness Scale (SSS)  ANCOVA | No effects of yoga or exercise on any cognitive function and alertness measures | There were no relative improvements of cognitive function in the yoga or exercise group compared to wait-list control |
| Podewils et al., 2005 [84]  USA  Prospective | To determine the association between physical activity and risk of dementia, Alzheimer’s disease, and vascular dementia | - N=3,375 - Sex: Male and female Age:>65 y - Characteristics: Healthy, free from dementia at baseline   Participants in the  Cardiovascular Health  Cognition Study 1992-2000 | Baseline and average 5.4 year follow-up  Measurements:  Interviews on energy expenditure and activity  - Modified Minnesota Leisure Time Activity Questionnaire (LTAQ)  -IADL  -3MS  -Cerebral MRI’s  -NINCDS-ADRDA  Cox proportional hazard models | 480 developed dementia over 5.4 y follow-up  The highest quartile of energy expenditure had RR 0.85 (95% CI, 0.61-1.19) of dementia vs. lowest quartile  Leisure time PA was on average 1,213 kcalwk  304 participants were sedentary  Participants in >4 activities had RR 0.51 (95% CI, 0.33-0.79) of dementia vs. 0-1 activity  Leisure time energy expenditure suggested an inverse relationship with Alzheimer’s disease and vascular dementia (not significant) | The study provides support for the hypothesis that engaging in a number of different physical activities protects against subsequent risk of all-cause dementia, Alzheimer’s disease, and vascular dementia over an average 5.4 year follow-up |
| Stevenson and Topp, 1990 [147]  USA | To determine the existence of differential effects of long term moderate or low intensity exercise on selected bio-behavioural variables | - N=72 - Sex: Male and female - Age: >60 y - Characteristics: Community dwellers | Baseline, 4.5 month and post 9 month intervention  **Moderate intensity**  60-70% HRR  3 times/wk  15 min warm-up, 30 min of stationary cycling, 10-15 min cool-down  **Low intensity**  30-40% HRR  3 times/wk  15 min warm-up, 30 min of stationary cycling, 10-15 min cool-down  Measurements:  -Life Satisfaction Index  -Health perception, 36 item tool  -Sleep patterns, self reported Likert-type instrument  -Mental status, Strub and Black mental status test  -CV fitness, stress test  ANOVA with RM | Most measures of CV functioning improved in both groups  Both groups improved equally in:  Attention/concentration [F(2,68) = 11.8, *p*<.01]  Short term memory  [F(2,68) = 20.22, *p*<.01]  Higher cognitive function  [F(2,68) = 5.38, *p*<.01] | The results of the study support the link between exercise and improved affective and cognitive functioning  The results may mean that the moderate exercise protocol was too conservative. Conversely, the findings may indicate that lower levels of exercise, which may be safer and more feasible over time, do improve fitness levels and prolong independent functioning |
| van Boxtel et al., 1997 [148]  Netherlands  Cross-sectional | To assess the relationship between aerobic capacity and cognitive performance | - N=132 (Age 65 y n=21, age 75 y n= 11) - Sex: Male and female - Age: 65 y, 75 y - Characteristics: Healthy with no morbidity related to brain health | Measurements:  -VO_2max_ estimation Siconolfi et al ., 1982  **Cognitive assessment:**  Groningen Intelligence Test (GIT)  Visual Verbal Learning Test (VVLT)  Verbal fluency  Simple psychomotor speed  Concept Shifting Test (CST)  Motor Choice Reaction Test (MCRT)  Letter Digit Substitution Test (LDST)  Stroop colour word test (SCWT)  ANCOVA, regression | VO_2max_ was strongly correlated with cognitive measures except word fluency | The findings add to the notion that aerobic fitness may selectively and age dependently act on cognitive processes, in particular those that require relatively large attentional resources |
| Van Gelder et al., 2004 [86]  Finland, Netherland, Italy | To investigate whether change in duration and intensity of physical activity is associated with 10 year cognitive decline | - N=295 - Sex: Men - Age: Born between 1900 and 1920 - Characteristics: Healthy survivors from the Finland, Italy and the Netherlands Elderly (FINE) Study | Baseline, 10 year follow-up  Measurements:  MMSE  PA, self administered questionnaire  Duration categorized into 4 groups:  <30 min/day  31-60 min/day  61-120 min/day  >120 min/day  Chi-square, linear regression | No difference in rate of cognitive decline between high or low duration activity  A decrease in activity duration of >60 min/day (half a SD) over 10 years caused a decline of 1.7 points (*p*<0.0001)  A decrease in intensity of more than half a SD (0.8 point) had a 2.3 point cognitive decline, 3.6 times stronger than those who maintained intensity | Even in old age, participants in activities of at least a medium-low intensity may postpone cognitive decline. A decrease in duration or intensity of PA results in a stronger cognitive decline than maintaining duration or intensity |
| Verghese et al., 2006 [81]  USA  Prospective cohort | To study the influence of leisure activity participation on risk of development of amnestic mild cognitive impairment (aMCI) | - N=437 - Sex: Men and women - Age: 75-85 y - Characteristics: Community residents initially free of dementia or aMCI | Baseline and mean 5.6±4.1 follow-up  Measurements:  IADL  Interview on 6 cognitive activities and 10 physical activities, scales were generated:  Cognitive Activity (0 to 42) Physical Activity (0 to 70)  aMCI assessment  Cox proportional-hazard regression | A 1 point increase on the Cognitive Activities Scale (HR 0.95, 95% CI 0.91-0.99) but not Physical Activities Scale (HR 0.97, 95% CI, 0.93-1.01) was associated with a lower risk of aMCI | Cognitive activity participation is associated with lower risk of developing aMCI |
| Williams and Lord, 1997 [97]  Australia  RCT | To determine whether a 12 month program of group exercise had beneficial effects on physiological and cognitive functioning and mood | - N=187 at baseline (n=149 post intervention) - Sex: Female - Age: Exerciser group mean 71.8 y, control group mean 71.6 y - Characteristics: Older community dwelling females | Pre and post 12 month intervention  2 groups:  **Exercise group (EG)**  2 times/wk for 1 hour for four 10-12 week periods  5 min warm-up, 35 min conditioning period, 15 stretching and 5-10 min relaxation/ cool-down    **Control group (CG)**  No organized activity  Measurements:  Medical examination  Physiological assessment:  Strength, reaction time,  Cognitive function, WAIS-R  Depression Anxiety and Stress Scale (DASS)  Well being - 5point scale  Chi-square, t-test, MANOVA | The EG showed a significant change from pre to post in:  (Pre post, mean ± SD)  Reaction time (ms)  275±35 vs. 271±32 (*p*<.05)  Muscle strength (kg)  88.8±23.6 vs. 103.9±28.0 (*p*<.01)  Digit span  15.21±3.45 vs. 16.25±3.8 (*p*<.01)  Anxiety  3.45±4.61 vs. 2.41±4.20 (*p*<.05) | The findings suggest that group exercise has beneficial effects on physiological and cognitive functioning and well being |

Age reported in years; RCT, Randomized Controlled Trial; HRR, heart rate reserve; BMI, body mass index (kg/m^2^); PA, physical activity; PAEE, physical activity energy expenditure; LPA, light physical activity; MVPA, moderate-vigorous physical activity; Sed, sedentary time; METs, Metabolic equivalent; HRR, heart rate reserve; MANOVA, multivariate analysis of variance; ANCOVA, Analysis of Covariance; CI, confidence interval; rmANOVA, repeated measures analysis of variance; yrs, years; BP, blood pressure; FEV_1_, forced expiratory volume; EEG, Electroencephalogram; p/w, per week; HR_max_, maximum heart rate; ANCOVA, analysis of covariance; MRI, Magnetic Resonance Imaging. CASI, Cognitive Abilities Screening Instrument; MMSE, Mini-Mental State Examination; mMMSE, modified Mini-Mental State Examination; ADL, activities of daily living; YPAS, Yale physical activity survey; WAIS-R, Wechsler Adult Intelligence Scale-Revised; BNT, Boston Naming Test; DSST, Digit Symbol Substitution Test; NART, National Adult Reading Test; CAMCOG, Cambridge Cognitive Examination for Mental Disorders of the Elderly; 1RM, 1 repetition maximum; WAIS-III, Wechsler Adult Intelligence Scale; WSM-R, Wechsler Memory Scale Revised; MHT, Moray House Test; BEC, not defined in manuscript; ADL, activities of daily living; CAS, Cognitive assessment scale; RPE, rate of perceived exertion; WMS, Wechsler Memory Scale; CPS, Cognitive Performance Scale; ACT, The Adult Changes in Thought study; CSHA, Canadian Study of Health and Aging; 3MS, Modified Mini-Mental State Examination; NINCDS-ADRDA, the National Institute of Neurological and Communicative Disorders and Stroke-Alzheimer’s Disease and Related Disorders Association; NINDS-AIREN, National Institute of Neurological Disorders and Stroke-Association Internationale pour la Recherche et l’Enseignement en Neurosciences; SSS, Stanford Sleepiness Scale; LTAQ, Leisure Time Activity Questionnaire; IADL, instrument for assessing basic activities of daily living; GIT, Groningen Intelligence Test; VVLT, Visual Verbal Learning Test; CST, Concept Shifting Test; MCRT, Motor Choice Reaction Test; LDST, Letter Digit Substitution Test; SCWT, Stroop colour word test.
