# Supplementary material for: Physical activity and functional limitations in older adults: a systematic review related to Canada's Physical Activity Guidelines
Source: Int J Behav Nutr Phys Act. 2010 May 11;7:38. doi: 10.1186/1479-5868-7-38 (PMC2882898; doi:10.1186/1479-5868-7-38)
Supplement: Additional file 9 — Supplemental table 11. Table s11: Cognitive studies assessed with the modified Downs and Black Quality Assessment Tool. [file 1479-5868-7-38-S9.DOC]

| Publication | **Reporting Score (11)** | **External Validity (2)** | **Internal Validity**  **- Bias (6)** | **Internal Validity**  **-Confounding (4)** | **Total (23)** |
| --- | --- | --- | --- | --- | --- |
| Aley et al., 2007 | 6 | 2 | 5 | 4 | 17 |
| Ari et al., 2004 | 5 | 2 | 4 | 4 | 15 |
| Benloucif et al., 2004 | 6 | 2 | 4 | 3 | 15 |
| Bixby et al., 2007 | 7 | 2 | 4 | 4 | 17 |
| Caplan and Ward, 1993 | 6 | 2 | 4 | 3 | 15 |
| Cassidy et al., 2004 | 6 | 2 | 4 | 3 | 15 |
| Hagen et al., 2003 | 6 | 2 | 4 | 3 | 15 |
| Hassmen et al., 1992 | 6 | 2 | 4 | 3 | 15 |
| Hatta et al., 2005 | 5 | 2 | 4 | 3 | 14 |
| Hill et al., 1993 | 6 | 2 | 4 | 4 | 16 |
| Hillman et al., 2002 | 7 | 2 | 4 | 4 | 17 |
| Hillman et al., 2006 | 7 | 2 | 4 | 4 | 17 |
| Newson et al., 2006 | 5 | 2 | 4 | 4 | 15 |
| Ojofeitimi et al., 2002 | 7 | 2 | 4 | 4 | 17 |
| Stevenson and Topp, 1990 | 6 | 2 | 4 | 3 | 15 |
| van Boxtel et al.,1997 | 7 | 2 | 4 | 4 | 17 |
| van Gelder et al., 2004 | 7 | 2 | 4 | 3 | 16 |

| **Publication** | Reporting (11) | **External Validity (2)** | **Internal Validity**  **-Bias (6)** | **Internal Validity**  **-Confounding (5)** | **Total (24)** |
| --- | --- | --- | --- | --- | --- |
| Blumenthal et al., 1989 | 8 | 2 | 4 | 5 | 19 |
| Cassilhas et al., 2007 | 8 | 2 | 4 | 4 | 18 |
| Fabre et al ., 2002 | 7 | 2 | 4 | 4 | 17 |
| Kalapotharakos et al., 2006 | 5 | 2 | 4 | 4 | 15 |
| Oken et al., 2006 | 8 | 2 | 4 | 5 | 19 |
| Williams and Lord,1997 | 6 | 2 | 4 | 4 | 16 |

| **Publication** | **Reporting Score (7)** | **External Validity (1)** | **Internal Validity**  **-Bias (4)** | **Total (12)** |
| --- | --- | --- | --- | --- |
| Abbott et al., 2004 | 6 | 1 | 3 | 10 |
| Barnes et al., 2003 | 6 | 1 | 3 | 10 |
| Broe et al ., 1998 | 5 | 1 | 3 | 9 |
| Bryan and Ward, 2002 | 4 | 1 | 3 | 8 |
| Deary et al., 2006 | 5 | 1 | 3 | 9 |
| Inzitari et al., 2007 | 7 | 1 | 3 | 11 |
| Landi et al., 2007 | 5 | 1 | 3 | 9 |
| Larson et al., 2006 | 6 | 1 | 3 | 10 |
| Lindsay et al., 2002 | 5 | 1 | 3 | 9 |
| Podewils et al.,2005 | 7 | 1 | 3 | 11 |
| Verghese et al ., 2006 | 6 | 1 | 3 | 10 |
